# Continuous Flow Generation of Acylketene Intermediates via Nitrogen Extrusion

**DOI:** 10.1021/acs.joc.2c01486

**Published:** 2022-09-01

**Authors:** Harry R. Smallman, Guilherme A. Brancaglion, Julio C. Pastre, Duncan L. Browne

**Affiliations:** † School of Pharmacy, University College London, 29-39 Brunswick Square, Bloomsbury, London WC1N 1AX, U.K.; ‡ Institute of Chemistry, University of Campinas−UNICAMP, Rua Monteiro Lobato 270, Campinas, São Paulo 13083-970, Brazil

## Abstract

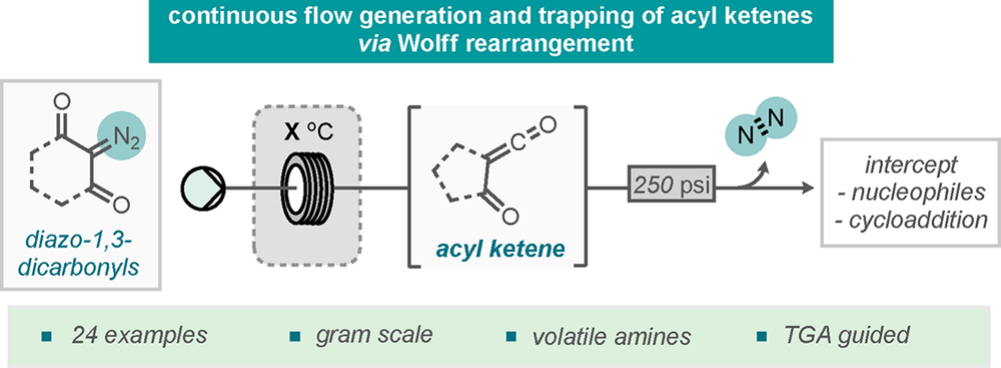

A flow chemistry process for the generation and use of acylketene precursors through extrusion of nitrogen gas is reported. Key to the development of a suitable continuous protocol is the balance of reaction concentration against pressure in the flow reactor. The resulting process enables access to intercepted acylketene scaffolds using volatile amine nucleophiles and has been demonstrated on the gram scale. Thermal gravimetric analysis was used to guide the temperature set point of the reactor coils for a variety of acyl ketene precursors. The simultaneous generation and reaction of two reactive intermediates (both derived from nitrogen extrusion) is demonstrated.

## Introduction

**Scheme 1:**
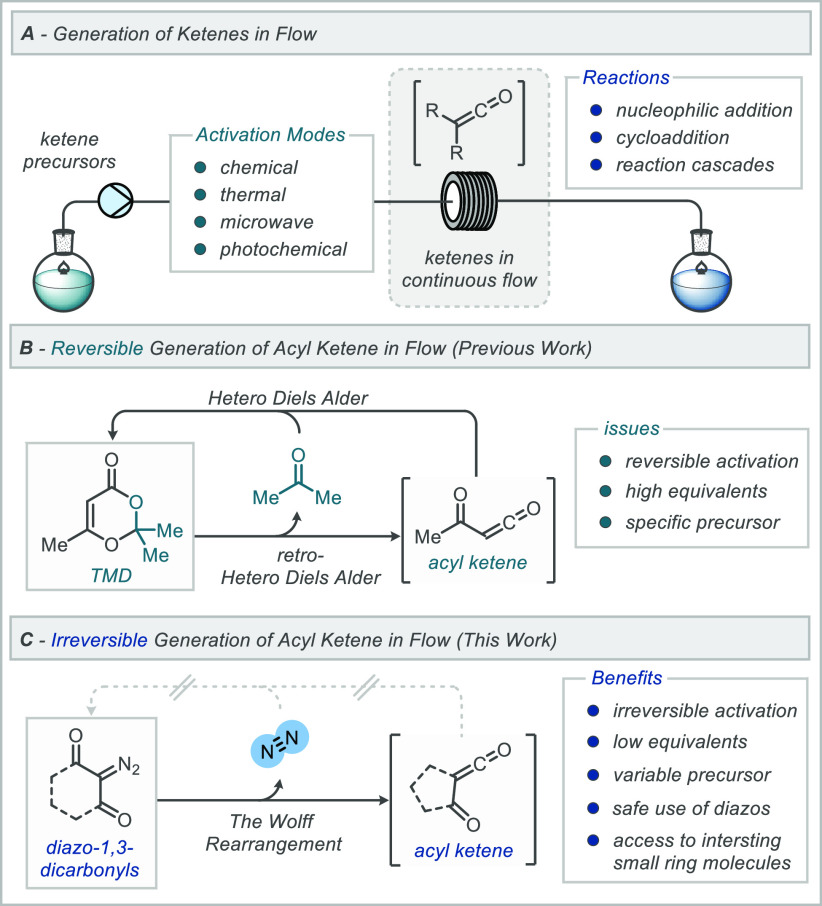
Generation and Use of Acyl Ketenes

Assessing the ability to generate and use reactive intermediates with developing and emerging chemical reactor advances represents a key strategy for the further improvement and understanding of those technologies.^1^ Reactive intermediates are an attractive testbed for reactor development processes, as often the required conditions or protocols can be “forbidden” by standard techniques owing to high temperatures, the liberation of gas, instability of intermediates or difficulty in scale-up, and often including a combination of these considerations.^2^ One class of these reactive intermediates that has received particular attention as a benchmarking tool for new or repurposed reactor types are ketenes.^3^ First postulated over a century ago by Wedekind and isolated by Staudinger in 1905, ketenes have been extensively studied and still feature as a powerful synthetic building block in organic synthesis.^4^ More recently, over the past decade, flow chemistry has proved to be a useful technique for generating and manipulating this group of reactive intermediates through chemical, thermal, microwave, and photochemical activation of ketene precursors.^5^ Ketene precursors bearing an α-carbonyl group lead to the generation of acyl ketenes where the α-carbonyl can be differentiated to access a range of ketene functionalities.^6^ Many ways to generate acyl ketenes have been demonstrated in traditional batch chemistry including thermolysis, photolysis, or treatment under basic or chemical conditions (Scheme 1A).^6^ The most common method for generating acyl ketenes is thermolysis; however, these methods require high temperatures and often generate volatile byproducts.^7^ Acyl ketenes can undergo a range of reactions including [3 + 2], [2 + 2], and [4 + 2] cycloadditions, nucleophilic additions to generate β-ketoproducts, and Friedel–Crafts acylation reactions.^8^

Previous work within the group has explored the use of flow chemistry for the generation and use of acyl ketenes. Here a robust flow system was developed and optimized for the generation of acyl ketene from commercially available 2,2,6-trimethyl-4*H*-1,3-dioxin-4-one (TMD, Scheme 1B).^9^ A wide range of applications of these acyl ketene intermediates was explored including dioxinone synthesis, β-keto esters, amides, and thioates as well as 1,3-oxazine-2,4-dione synthesis.

However, this method proved to have limitations. For example, the release of stoichiometric acetone into the reaction without the ability for it to be removed from the reaction stream readily permitted the reverse hetero Diels–Alder reaction to regenerate the TMD starting material. This was only overcome by the use of high equivalents of the ketene trapping reactant. The unique precursor also requires further functionalization, as there are no similar substructures commercially available.^9^ Accordingly, it was hypothesized that removing the reversible pathway would permit cleaner conversion to the acyl ketene intermediate.

To explore this notion, 2-diazo-1,3-carbonyls were chosen where, upon heating, this class of precursor releases molecular nitrogen and undergoes a Wolff rearrangement to form the acyl ketene (Scheme 1C).^10^ In traditional batch methods, the sudden release of nitrogen gas would pose significant safety considerations, especially when performed on a large scale. However, it was envisioned that flow chemistry would allow for the safe and controlled release of nitrogen and generation of the reactive intermediate.^11^ The use of a pressurized flow system would also allow for heating solvents above atmospheric boiling points, giving access to the higher temperatures needed to undergo nitrogen extrusion and rearrangement.^12^

## Results and Discussion

Our study began with exploring a suitable flow reactor setup and parameters for the generation and interception of an acyl ketene derived from diazodimedone precursor (**1a**). Initially, this material was prepared by treatment of the respective dicarbonyl compound with sodium azide using known procedures.^13^ Thermal gravimetric analysis (TGA) of this precursor was obtained to establish a safe operating protocol for handling and storage of this diazo compound, but also, the onset temperature that TGA provides gives a good representation of the temperature at which nitrogen is extruded from the molecule to form the acyl ketene.^14^ The onset temperature of decomposition of diazodimedone **1a** is 127 °C, and this dictated reactor temperatures for our initial flow system for the generation and trapping of the acyl ketene.

**Scheme 2:**
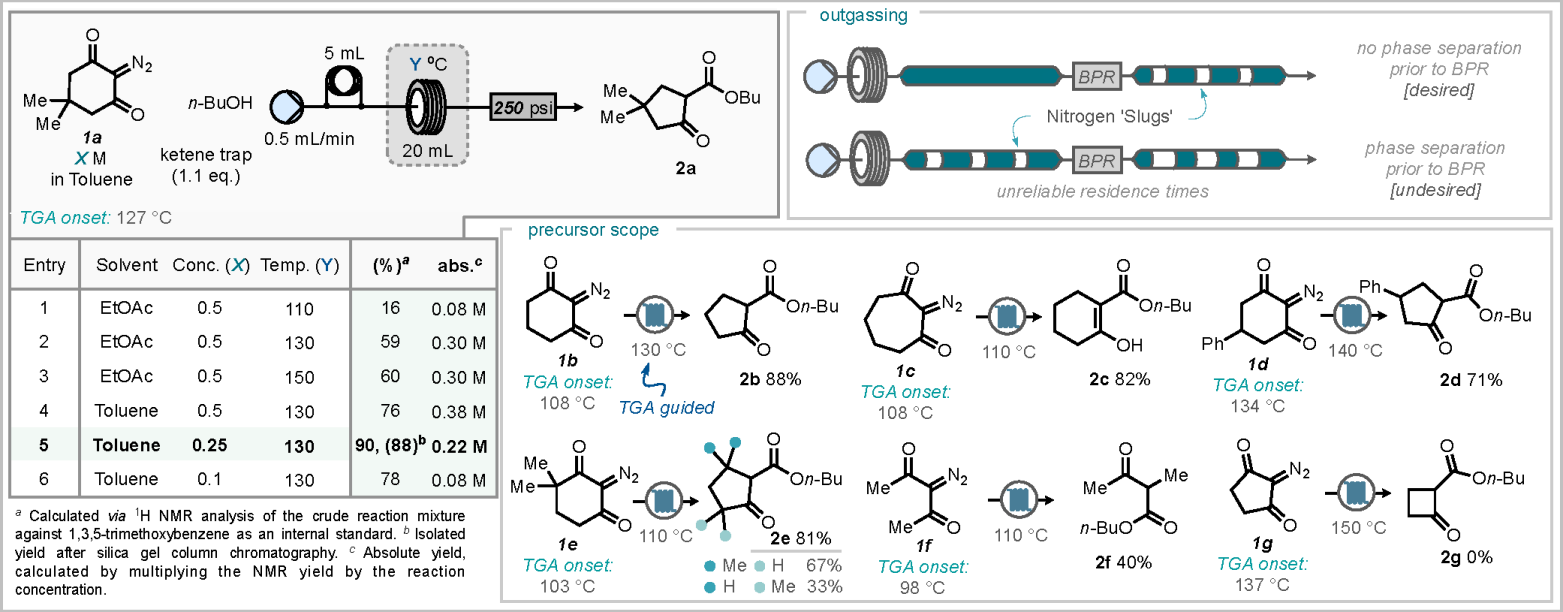
Optimization of Continuous Flow Generation and Trapping of Acylketene Intermediates

For the optimization, acyl ketene precursor **1a** and 1.1 equiv of *n*-butanol were loaded into a 1 mL loading loop and injected into a continuous stream of ethyl acetate pumping at 0.5 mL/min. The reaction slug was passed through a 20 mL heated reactor coil at a range of temperatures. At 110 °C, the reaction proceeded slowly with 16% of the desired product **2a** observed (entry 1, Scheme 2), whereas heating the reactor above the onset temperature (127 °C) to 130 °C afforded the trapped ketene in 59% yield (entry 2, Scheme 2). Heating beyond this temperature (to 150 °C) gave a minimal increase in yield (entry 3, Scheme 2).^15^ Changing the solvent to toluene afforded an increase in yield (76% yield, entry 4, Scheme 2). At this point, inspection of the flow reactor identified that significant outgassing could be observed before the back pressure regulator (BPR), leading to irregularities in the residence time (Scheme 2). The quantity of nitrogen released in combination with heating toluene above its atmospheric boiling point led to the formation of nitrogen “slugs”. To address this, the concentration of the starting material (**1a**) was decreased, where at a concentration of 0.25 M, an isolated yield of 88% of the desired product could be achieved (entry 5). Notably, under these conditions no outgassing was observed, highlighting a reaction process with the potential to be run continuously. Decreasing the concentration further to 0.1 M afforded lower yields. The absolute yields are also provided in Scheme 2, whereby the concentration of product in the output stream is identified; conversions should be compared on the same basis and run with the same concentration of limiting reagent.

Once optimal conditions had been identified, a range of other acyl ketene diazo precursors **1b**–**1g** were synthesized and analyzed using TGA. The precursors were then subjected to the same flow conditions, where the temperature was varied depending on the respective TGA onset temperature (Scheme 2). Good yields were achieved for all cyclohexanedione precursors **1b**, **1d**, **1e**, as well as the larger cycloheptanedione **1c**. It is worth noting that a higher temperature was required for diazocyclohexanedione **1b** to achieve full conversion. The acyclic precursor, diazoacetylacetone **1f**, gave a comparably lower yield of 40%, which could be attributable to the stability of the acyl ketene without a cyclic backbone. The final precursor, diazocyclopentanedione **1g**, gave none of the desired cyclobutanone product **2g** and just remaining starting material even when heating to 150 °C. Closer inspection of the TGA trace for **1g** highlights that the loss of nitrogen does not coincide with the onset at 137 °C, and we attribute this instead to a boiling of the sample, which would explain recovery of starting material from flow experiments under pressure regulation—see SI for TGA traces.

**Scheme 3:**
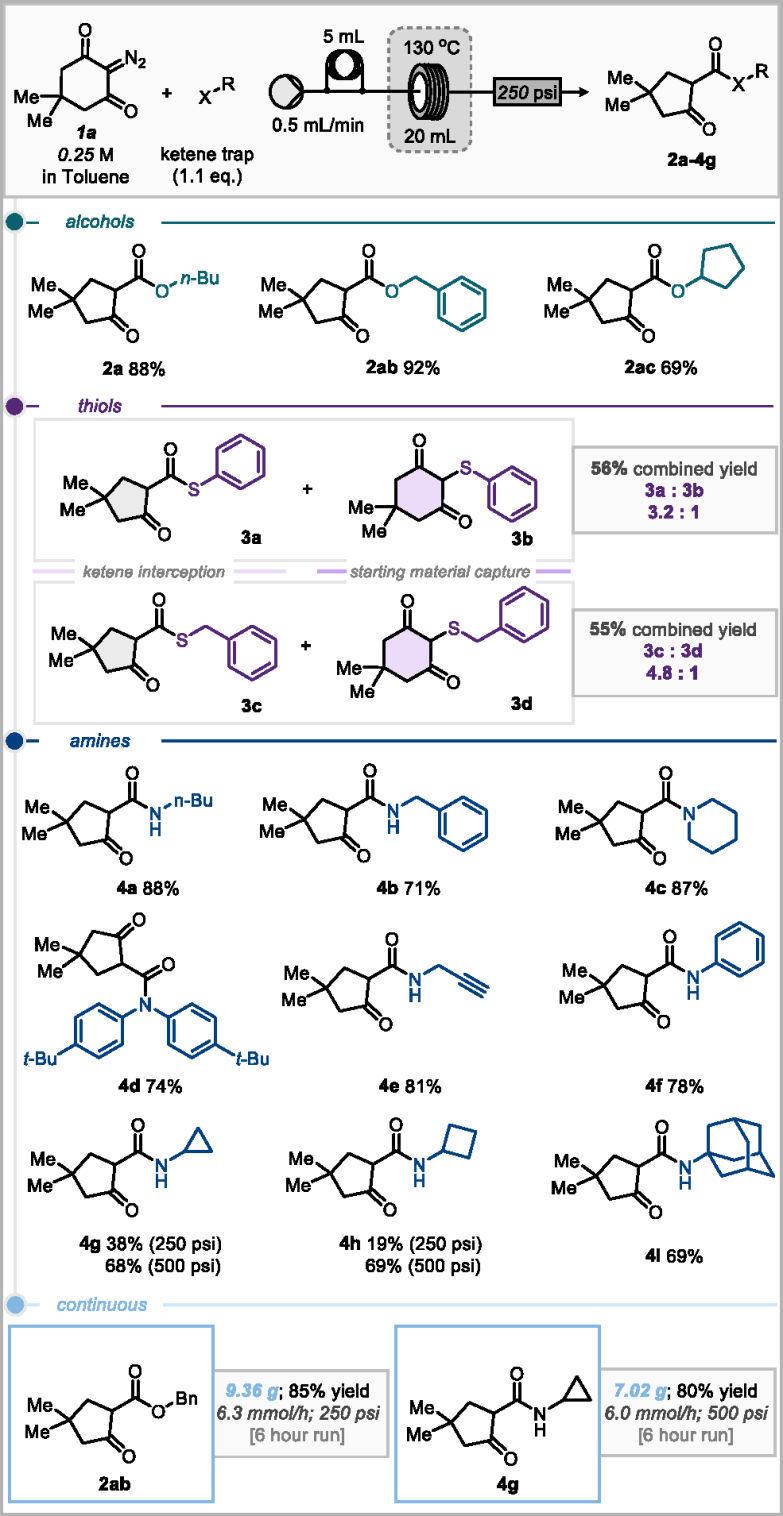
Scope of Nucleophiles and Scale-up Examples

The optimized flow conditions were then used to explore the scope of the reaction between diazodimedone **1a** and a range of different nucleophiles (Scheme 3). Good yields were demonstrated using primary (**2a**), secondary (**2ac**), and benzylic alcohols (**2ab**) with the greatest yield of 92% observed with benzyl alcohol. Thiol nucleophiles were also explored. However, a mixture of inseparable products were found deriving from generation and interception of the desired acyl ketene but also direct substitution of the diazo group in the starting material. In both thiophenol and benzyl mercaptan reactions, the product arising from acyl ketene formation was the major product (**3a** and **3c**, respectively). Amine nucleophiles performed well under the developed flow conditions, with a range of different amines including primary (**4a**, **4e**, and **4g**–**i**), secondary (**4c**), benzylic (**4b**), anilines (**4f**), and bulky bis-*t*-Bu dipheynylamine (**4d**) proceeding well. Notably, volatile amines such as cyclopropyl amine (**4g**) (bp 49–50 °C) and cyclobutylamine (**4h**) (bp 81–82 °C), those that would not work in a typical open-flask batch reactor, did work under these flow conditions. Although in these cases minor outgassing was observed, leading to lower yields, improved yields from these amines could be achieved by increasing the pressure tolerance of the system by using a 500 rather than a 250 psi BPR.

The designed flow process for the generation and use of acyl ketene from diazodimedone **1a** was also demonstrated on an increased scale without the need for reoptimization. In this case, the premixed reagents were directly pumped into the reactor system (rather than using the loading loops for smaller injection segments) using benzyl alcohol and cyclopropylamine as the respective nucleophiles. In both cases, the reaction was continuously run for 6 h affording over 9 g of the alcohol trapped product **2b** and 7 g of volatile cyclopropylamine trapped product **4g** (Scheme 3). This gave an overall productivity of 6.3 mmol/h when using benzyl alcohol and 6.0 mmol/h when cyclopropylamine was employed as the nucleophile.

**Scheme 4:**
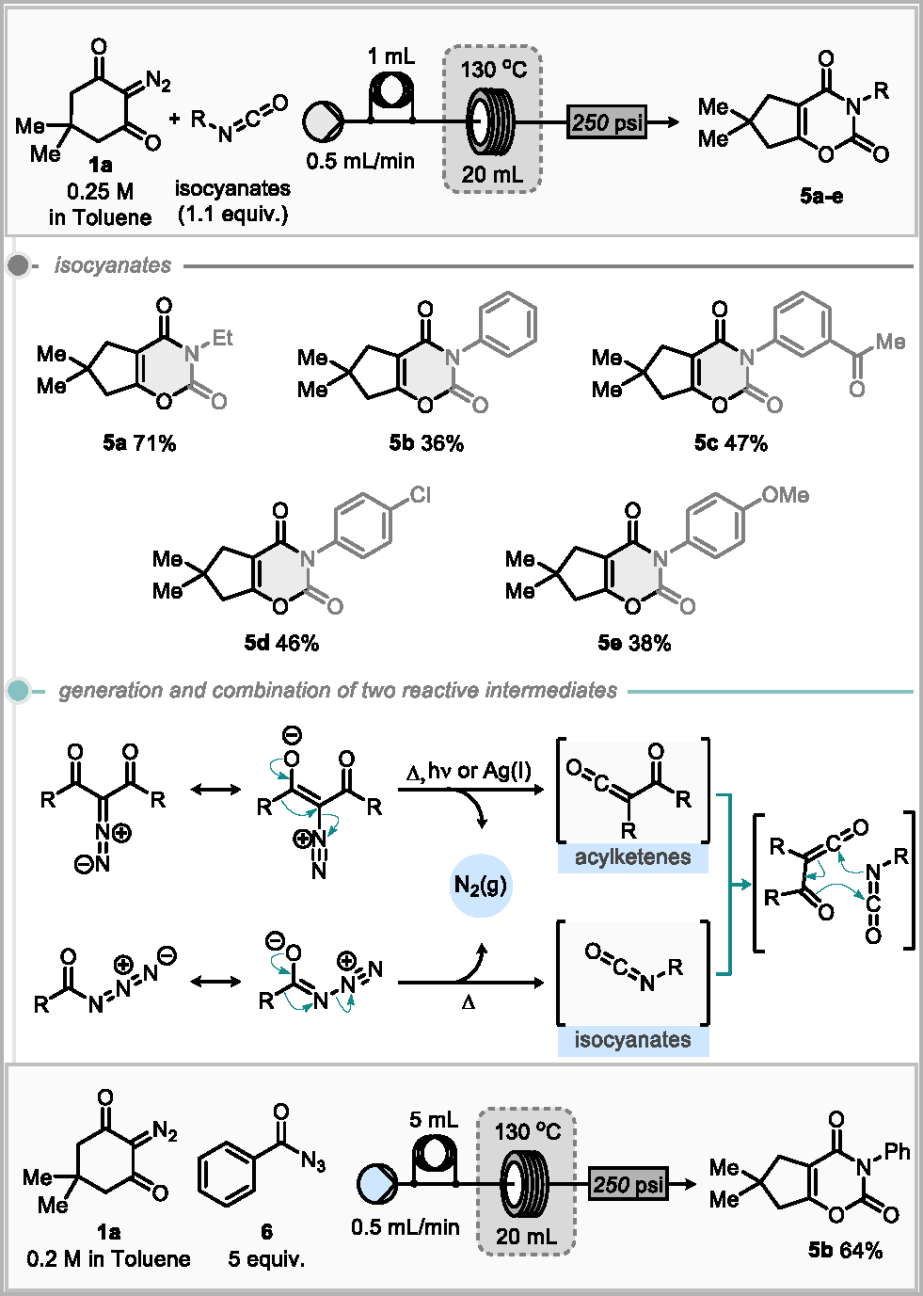
Isocyanate Cycloadditions and Generation and Combination of Two Reactive Intermediates

The reaction conditions were then applied to a cycloaddition reaction of the acylketene with isocyanates to form 1,3-oxazine-2,4-dione motifs.^16^ Ethyl isocyanate provided the greatest yield of 71% (**5a**, Scheme 4). However, diminished yields of 36–47% were observed when using aryl isocyanates (**5b**–**e**). As a final challenge to the reactor design, we investigated the simultaneous generation and combination of two reactive intermediates, namely, unveiling of the acylketene at the same time as generating an isocyanate in situ via the Curtius rearrangement of an acyl azide precursor (Scheme 4).^17^ Such a reaction would generate 2 equiv of nitrogen gas and require high reaction temperatures to initiate formation of the reactive intermediates. Thus, such a process may be considered too high risk to approach in a typical batch reactor vessel. Phenyl acyl azide **6** was chosen as the precursor for the generation of isocyanate. Early efforts, using the previously optimal conditions, identified issues with outgassing of nitrogen prior to the pressure regulator, and reoptimization was required of both concentration and stoichiometry. Lowering the concentration to 0.05 M showed no outgassing and gave a promising yield of 46% of **5b**. Incremental increases in the concentration gave improvements in yields up to a maximum of 64% of **5b** at 0.2 M. Running the reaction with a 500 psi back pressure regulator allowed greater concentrations to be used with no outgassing; however, this gave no increase in yield. While we could demonstrate this interesting concept, it is clear that practical implementation of this process for substrate scope would require the synthesis of a range of acyl azides, many of which could be unknown and present potential hazards, so we opted not to further explore this line of enquiry. Notably, Watts and co-workers have reported a continuous preparation and purification process for acyl azides, but we might caution against a more general practice of isolating unknown acyl azides at appreciable quantities.^18^

## Conclusion

In conclusion, the use of continuous flow processing was demonstrated for the generation of acyl ketene species via nitrogen extrusion and their application for the synthesis of β-ketoesters, β-ketoamides, 1,3-oxazine-2,4-diones, and less successfully to β-ketothioates. Fine control over the quantity of nitrogen gas generated through variables such as reaction concentration and reactor back-pressure is imperative to delivering a robust and scalable process, which has been demonstrated on two 6 h continuous runs. Finally, the simultaneous generation and combination of two reactive intermediates via nitrogen extrusion has also been demonstrated.

## Experimental Section

### Reagents

Reagents were purchased from Fluorochem Ltd., Sigma-Aldrich (Merck), or Fisher Scientific and used as received.

### Flow Chemistry Equipment

The flow setup consisted of PTFE tubing of an 0.8 mm internal diameter and one HPLC pump. Sample loops of 1 or 5 mL (PTFE) were used to load the reagents. A 20 mL stainless steel residence coil was used. The temperature of the flow residence coil was controlled using a CRD Polar Bear Plus device. Back pressure regulators of 250 or 500 psi were used. See the Supporting Information for further details.

### Analytical Equipment

Proton and carbon NMR spectra were recorded on a Bruker Avance 400 MHz (^1^H NMR at 400 MHz, ^13^C NMR at 101 MHz) spectrometer equipped with broadband and selective (^1^H and ^13^C) inverse probes or a Bruker Avance 500 MHz (^1^H NMR at 500 MHz, ^13^C NMR at 126 MHz) spectrometer equipped with a QNP (31P, ^13^C, ^15^N, ^1^H) cryoprobe. Chemical shifts for protons are reported in parts per million downfield from Si(CH_3_)_4_ and are referenced to residual protium in the deuterated solvent (CHCl_3_ at 7.26 ppm, DMSO at 3.31 (H_2_O), 2.50, acetone-*d*_6_ depending on solvent used). NMR data are presented in the following format: chemical shift (multiplicity [app = apparent, br = broad, d = doublet, t = triplet, q = quartet, quint = quintet, sext = sextet, dd = doublet of doublets, dt = doublet of triplets, ddd = doublet of doublet of doublets, m = multiplet], coupling constant [in Hz], number of equivalent nuclei by integration). Analytical thin-layer chromatography was performed on Merck silica gel 60zf F254 plates and visualized with UV light (254 or 365 nm). Flash chromatography was performed on a Biotage Selekt. Samples were dried onto silica gel prior to addition to column. Solvents were removed under reduced pressure using Heidolph Rotavapor apparatus. Thermal Gravimetric Analysis (TGA) was performed on a TA Instrument TGA 550 using aluminum pans and a temperature ramp of 10 °C min^–1^. The data were processed using TA Instruments Trios V4.5.1.42498 to yield the onset temperatures. High resolution mass spectral (HRMS) data were obtained on a Micromass Q-TOF Premier Tandem Mass Spectrometer coupled to Nano Acquity LC.

### Synthetic Procedures and Characterization Data

#### General Procedure A for the Synthesis of Diazo-1,3-dicarbonyls

To a round-bottom flask equipped with an appropriate stirring bar were added 1,3-carbonyl (1 equiv) and MeCN (50 mL). Tosyl azide (1 equiv) and K_2_CO_3_ (1.1 equiv) were successively added, and the mixture was stirred for 13 h at room temperature. The mixture was filtered through a pad of silica gel, rinsed with CH_2_Cl_2_, and concentrated under a vacuum to give the crude product. The crude residue was then purified via silica gel chromatography (Hexane:EtOAc, 100:0–70:30 v:v) to afford the pure diazo-1,3-dicarbonyl.

#### Synthesis of Diazodimedone (**1a**)

General procedure A was followed using dimedone (0.911 g, 6.5 mmol). Silica gel chromatography (Hexane:EtOAc, 100:0–70:30 v:v) gave **1a** as a pale green solid, 84% (0.905 g, 5.45 mmol). TGA Onset 127 °C. ^1^H NMR (400 MHz, CDCl_3_) δ 2.44 (s, 4H), 1.12 (s, 6H). ^13^C{H} NMR (126 MHz, CDCl_3_) δ 190.0, 83.9, 50.7, 31.3, 28.5. IR (neat) ν_max_ 2960, 2892, 2188, 2140, 1632, 1305, 1264, 1137, 1047, 878, 812, 700. Data are consistent with literature precedent.^19^

#### Synthesis of 2-Diazocyclohexane-1,3-dione (**1b**)

General procedure A was followed using cyclohexane-1,3-dione (0.560 g, 5 mmol). Silica gel chromatography (Hexane:EtOAc, 100:0–70:30 v:v) gave **1b** as a light brown solid, 41% (338 mg, 2.05 mmol). TGA Onset 108 °C. ^1^H NMR (400 MHz, CDCl_3_) δ 2.56 (t, *J* = 6.4 Hz, 4H), 2.03 (quint, *J* = 6.4, 2H). ^13^C{H} NMR (100 MHz, CDCl_3_) δ 190.5, 85.1, 36.9, 18.7. IR (neat) ν_max_ 2199, 2132, 1625, 1282, 1174, 995, 861, 685 cm^–1^. Data are consistent with literature precedent.^19^

#### Synthesis of 2-Diazocycloheptane-1,3-dione (**1c**)

General procedure A was followed using cycloheptane-1,3-dione (1.51 g, 12 mmol). Silica gel chromatography (Hexane:EtOAc, 100:0–70:30 v:v) gave **1c** as a yellow oil, 48% (876 mg, 5.76 mmol). TGA Onset 108 °C. ^1^H NMR (400 MHz, CDCl_3_) δ 2.71 (br s, 4H), 1.91 (br s, 4H). ^13^C{H} NMR (100 MHz, CDCl_3_) δ 192.1, 90.8, 40.1, 21.2. IR (neat) ν_max_ 2146, 1625, 1247, 1177, 902 cm^–1^. Data are consistent with literature precedent.^20^

#### Synthesis of 2-Diazo-5-phenylcyclohexane-1,3-dione (**1d**)

General procedure A was followed using 5-phenylcyclohexane-1,3-dione (1.13 g, 6 mmol). Silica gel chromatography (Hexane:EtOAc, 100:0–70:30 v:v) gave **1d** as a white solid, 76% (979 mg, 4.56 mmol). TGA Onset 134 °C. ^1^H NMR (400 MHz, CDCl_3_) δ 7.36 (t, *J* = 7.2 Hz, 2H), 7.29 (d, *J* = 7.2 Hz, 1H), 7.21 (d, *J* = 7.2 Hz, 2H), 3.50–3.31 (m, 1H), 2.81 (ddd, *J* = 28.6, 17.0, 7.9 Hz, 4H). ^13^C{H} NMR (100 MHz, CDCl_3_) δ 189.3, 141.2, 129.1, 127.5, 126.5, 84.8, 44.2, 36.5. IR (neat) ν_max_ 2121, 1632, 1289, 1043, 767, 704 cm^–1^. Data are consistent with literature precedent.^20^

#### Synthesis of 2-Diazo-4,4-dimethylcyclohexane-1,3-dione (**1e**)

General procedure A was followed using 4,4-dimethylcyclohexane-1,3-dione (0.841 g, 6 mmol). Silica gel chromatography (Hexane:EtOAc, 100:0–70:30 v:v) gave **1e** as a yellow solid, 75% (748 mg, 4.5 mmol). TGA Onset 103 °C. ^1^H NMR (400 MHz, CDCl_3_) δ 2.57 (t, *J* = 6.6 Hz, 2H), 1.85 (t, *J* = 6.6 Hz, 2H), 1.21 (s, 6H). ^13^C{H} NMR (100 MHz, CDCl_3_) δ 195.4, 190.4, 84.0, 41.6, 33.6, 32.8, 24.5. IR (neat) ν_max_ 2124, 1650, 1632, 1282, 1233, 1203, 1162, 987 cm^–1^. Data are consistent with literature precedent.^21^

#### Synthesis of 3-Diazopentane-2,4-dione (**1f**)

General procedure A was followed using acetylacetone (2.03 g, 20 mmol). Silica gel chromatography (Hexane:EtOAc, 100:0–70:30 v:v) gave a yellow oil, 83% (2.09 g, 16.6 mmol). TGA Onset 98 °C. ^1^H NMR (500 MHz, CDCl_3_) δ 2.46. ^13^C{H} NMR (126 MHz, CDCl_3_) δ 188.3, 84.1, 28.5. IR (neat) ν_max_ 2922, 2126, 1664, 1658, 1365, 1304, 1237, 1167, 964, 931, 605 cm^–1^. Data are consistent with literature precedent.^19^

#### Synthesis of 2-Diazocyclopentane-1,3-dione (**1g**)

General procedure A was followed using cyclopentane-1,3-dione (0.785 g, 8 mmol). Silica gel chromatography (Hexane:EtOAc, 100:0–70:30 v:v) gave **1g** as a light yellow solid, 52% (521 mg, 4.16 mmol). TGA Onset 137 °C. ^1^H NMR (400 MHz, CDCl_3_) δ 2.69 (br s, 4H). ^13^C{H} NMR (100 MHz, CDCl_3_) δ 193.1, 75.0, 33.9. IR (neat) ν_max_ 2132, 1654, 1312, 1222, 1054, 991. 808 cm^–1^. Data are consistent with literature precedent.^20^

#### General Procedure B for the Synthesis of β-Dicarbonyl Products

The corresponding diazo-1,3-dicarbonyl (1.25 mmol) and the corresponding nucleophile (1.37 mmol, 1.1 equiv) were mixed in toluene (5 mL, 0.25 M) and loaded into a 5 mL loading loop. The loop was injected into a stream of toluene at 0.5 mL min^–1^ and heated to the TGA guided temperature for 40 min using a 20 mL stainless steel coil reactor loaded onto a CRD Polar Bear device. The products were purified by silica gel column chromatography using Hexane/EtOAc (100:0–80:20 v:v) as solvent elution.

#### Synthesis of Butyl 4,4-dimethyl-2-oxocyclopentane-1-carboxylate (**2a**)

General procedure B was followed using diazodimedone (**1a**, 0.208 g, 1.25 mmol), *n*-butanol (0.096g, 1.37 mmol) and a temperature of 130 °C. Silica gel chromatography (Hexane:EtOAc, 100:0–80:20 v:v) gave **2a** as a pale red oil, 88% (233 mg, 1.1 mmol). ^1^H NMR (500 MHz, CDCl_3_) δ 4.18–1.09 (q, *J* = 6.6 Hz, 2H), 3.36 (dd, *J* = 11.0, 8.8 Hz, 1H), 2.23–2.04 (m, 4H), 1.63 (p, *J* = 6.7 Hz 2H), 1.39 (h, *J* = 7.4 Hz, 2H), 1.23 (s, 3H), 1.04 (s, 3H), 0.93 (t, *J* = 7.4 Hz, 3H) ppm. ^13^C{H} NMR (126 MHz, CDCl_3_) δ 211.8, 169.6, 65.3, 54.4, 53.1, 40.9, 34.5, 30.6, 29.0, 27.7, 19.0, 13.7 ppm. IR (neat) ν_max_ 2960, 2874, 1752, 1726, 1465, 1372, 1335, 1308, 1256, 1223, 1167, 1118, 1062, 958 cm^–1^. HRMS (ESI-TOF) *m*/*z* [M + Na^+^] calcd for C_12_H_20_O_3_Na 235.1310, found 235.1315.

#### Synthesis of Butyl 2-oxycyclopentanecarboxylate (**2b**)

General procedure B was followed using 2-diazocyclohexane-1,3-dione (**1c**, 0.173 g, 1.25 mmol), *n*-butanol (0.096g, 1.37 mmol) and a temperature of 130 °C. Silica gel chromatography (Hexane:EtOAc, 100:0–80:20 v:v) gave **2b** as a colorless oil, 88% (202 mg, 1.1 mmol). ^1^H NMR (400 MHz, CDCl_3_) δ 4.16–4.06 (m, 2H), 3.12 (t, *J* = 8.9 Hz, 1H), 2.29–2.24 (m, 4H), 2.11 (tt, *J* = 12.3, 7.1 Hz, 1H), 1.84 (dt, *J* = 12.3, 7.1 Hz, 1H), 1.60 (quint, J = 7.4 Hz, 2H), 1.36 (d, *J* = 7.4 Hz, 2H), 0.91 (t, *J* = 7.4 Hz, 3H). ^13^C{H} NMR (100 MHz, CDCl_3_) δ 212.4, 169.5, 65.3, 54.8, 38.1, 30.6, 27.5, 21.0, 19.1, 13.7 cm^–1^. IR (neat) *v*_max_ 2959, 2877, 1751, 1722, 1248, 1185, 1148, 1110. HRMS (ESI-TOF) *m*/*z* [M + Na^+^] calcd for C_10_H_16_O_3_Na 207.0997, found 207.0988.

#### Synthesis of Butyl 2-hydroxycyclohex-1-enecarboxylate (**2c**)

General procedure B was followed using 2-diazocycloheptane-1,3-dione (**1c**, 0.190 g, 1.25 mmol), *n*-butanol (0.096g, 1.37 mmol) and a temperature of 110 °C. Silica gel chromatography (Hexane:EtOAc, 100:0–80:20 v:v) gave **2c** as a colorless oil, 82% (203 mg, 1.03 mmol). ^1^H NMR (400 MHz, CDCl_3_) δ 12.23 (s, 1H), 4.15 (t, *J* = 6.6 Hz, 2H), 2.24 (dt, *J* = 12.6, 5.6 Hz, 4H), 1,69–1,57 (m, 6H), 1.40 (sex, *J* = 7.4 Hz, 2H), 0.94 (t, *J* = 7.4 Hz, 3H). ^13^C{H} NMR (100 MHz, CDCl_3_) δ 172.9, 172.0, 97.9, 64.1, 30.8, 29.2, 22.5, 22.5, 22.0, 19.3, 13.9. IR (neat) *v*_max_ 2937, 2862, 1714, 1651, 1613, 1297, 1259, 1215, 1174, 1080 cm^–1^. HRMS (ESI-TOF) *m*/*z* [M + Na^+^] calcd for C_11_H_18_O_3_Na 221.1154, found 221.1150.

#### Synthesis of Butyl 2-oxo-4-phenylcyclopentanecarboxylate (**2d**)

General procedure B was followed using 2-diazo-5-phenylcyclohexane-1,3-dione (**1d**, 0.268 g, 1.25 mmol), *n*-butanol (0.096g, 1.37 mmol) and a temperature of 140 °C. Silica gel chromatography (Hexane:EtOAc, 100:0–80:20 v:v) gave **2d** as a white solid, 71% (232 mg, 0.89 mmol). mp 47–51 °C. ^1^H NMR (400 MHz, CDCl_3_) δ 7.36 (q, *J* = 7.0 Hz, 2H), 7.33–7.22 (m, 3H), 4.26–4.08 (m, 2H), 3.46–3.28 (m, 2H), 2.79 (dd, *J* = 18.5, 7.1 Hz, 1H), 2.69 (dt, *J* = 14.2, 7.1 Hz, 1H), 2.57–2.38 (m, 2H), 1.67 (quint, *J* = 7 Hz, 2H), 1.42 (d, *J* = 7 Hz, 2H), 0.96 (t, *J* = 7 Hz, 3H). ^13^C{H} NMR (100 MHz, CDCl_3_) δ 210.0, 169.2, 141.8, 128.9, 126.9, 126.7, 65.4, 56.4, 45.8, 39.9, 35.0, 30.7, 19.1, 13.7. IR (neat) *v*_max_ 2967, 2858, 1751, 1722, 1453, 1338, 1237, 1159, 1107, 760, 693 cm^–1^. HRMS (ESI-TOF) *m*/*z* [M + Na^+^] calcd for C_16_H_20_O_3_Na 283.1310, found 283.1309.

#### Synthesis of Butyl 2,2-dimethyl-5-oxocyclopentanecarboxylate (**2ea**) and Butyl 3,3-dimethyl-2-oxocyclopentanecarboxylate (**2eb**)

General procedure B was followed using 2-diazo-4,4-dimethylcyclohexane-1,3-dione (**1e**, 0.208 g, 1.25 mmol), *n*-butanol (0.096g, 1.37 mmol) and a temperature of 110 °C. Silica gel chromatography (Hexane:EtOAc, 100:0–80:20 v:v) gave an inseparable mixture of **2ea** and **2eb** as a colorless oil, 81% (**2ea**:**2eb** 2:1, 214 mg, 1.01 mmol). ^1^H NMR (400 MHz, CDCl_3_) δ 4.21–4.01 (m, 2H 2ea, 2H 2eb), 3.21(t, *J* = 9.0 Hz, 1H 2eb) 2.86 (s, 1H 2ea), 2.50–2.10 (m, 2H 2ea, 2H 2eb), 2.03–1.88 (m, 2H 2eb), 1.79–1.52 (m, 4H 2ea, 2H 2eb), 1.36 (m, 2H 2ea, 2H 2eb), 1.17 (m, 3H 2ea), 1.10–1.02 (m, 3H 2ea, 6H 2eb), 0.90 (t, *J* = 7.5 Hz, 3H 2ea, 3H 2eb) ppm. ^13^C{H} NMR (126 MHz, CDCl_3_) δ 215.9, 213.0, 169.8, 168.9, 65.8, 65.2, 64.8, 63.5, 54.3, 40.8, 36.8, 36.7, 36.5, 35.9, 30.6, 29.0, 24.2, 24.1, 19.2, 19.1, 13.7 ppm. IR (neat) ν_max_ 2959, 2870, 1751, 1722, 1218, 1159, 1058 cm^–1^. HRMS (ESI-TOF) *m*/*z* [M + Na^+^] calcd for C_12_H_20_O_3_Na 235.1310, found 235.1308.

#### Synthesis of Butyl 2-methyl-3-oxobutanoate (**2f**)

General procedure B was followed using 3-diazopentan-2,4-dione (**1f**, 0.156 g, 1.25 mmol), *n*-butanol (0.096 g, 1.37 mmol) at a temperature of 110 °C. Silica gel chromatography (Hexane:EtOAc, 100:0–80:20 v:v) gave **2f** as a colorless oil, 40% (83 mg, 0.50 mmol). ^1^H NMR (400 MHz, CDCl_3_) δ 4.12 (t, *J* = 6.5 Hz, 2H), 3.49 (q, *J* = 7.4 Hz, 1H), 2.22 (s, 3H), 1.61 (quint, *J* = 7.4 Hz, 2H), 1.36 (sex, *J* = 7.4 Hz, 2H), 1.32 (d, *J* = 7.1 Hz, 3H), 0.92 (t, *J* = 7.4 Hz, 3H). ^13^C{H} NMR (100 MHz, CDCl_3_) δ 203.7, 170.7, 65.3, 53.7, 30.6, 28.5, 19.1, 13.7, 12.8. IR (neat) ν_max_ 2959, 2877, 1720, 1714, 1241, 1200, 1148, 1073 cm^–1^. HRMS (ESI-TOF) *m*/*z* [M + Na^+^] calcd for C_9_H_16_O_3_Na 195.0997, found 195.0990.

#### Synthesis of Benzyl 4,4-dimethyl-2-oxocyclopentane-1-carboxylate (**2ab**)

General procedure B was followed using diazodimedone (**1a**, 0.208 g, 1.25 mmol), benzyl alcohol (0.147 g, 1.37 mmol) and a temperature of 130 °C. Silica gel chromatography (Hexane:EtOAc, 100:0–80:20 v:v) gave **2ab** as a pale yellow oil, 92% (283 mg, 1.15 mmol). ^1^H NMR (500 MHz, CDCl_3_) δ 7.37 (d, *J* = 4.4 Hz, 1H), 7.35–7.30 (m, 1H), 5.18 (s, 1H), 3.47–3.40 (m, 1H), 2.26–2.18 (m, *J* = 10.9, 9.6 Hz, 1H), 2.09 (ddd, *J* = 13.0, 8.8, 1.3 Hz, 1H), 1.22 (s, 1H), 1.05 (s, 1H). ^13^C{H} NMR (126 MHz, CDCl_3_) δ 211.6, 169.5, 135.7, 128.7, 128.4, 128.3, 67.2, 54.4, 53.2, 40.9, 34.7, 29.1, 27.8. IR (neat) ν_max_ 2956, 2870, 1752, 1726, 1454, 1308, 1163, 749, 701 cm^–1^. HRMS (ESI-TOF) *m*/*z* [M + Na^+^] calcd for C_15_H_18_O_3_ 269.1154, found 269.1164.

#### Synthesis of Cyclopentyl 4,4-dimethyl-2-oxocyclopentane-1-carboxylate (**2ac**)

General procedure B was followed using diazodimedone (**1a**, 0.208 g, 1.25 mmol), cyclopentanol (0.118 g, 1.37 mmol) and a temperature of 130 °C. Silica gel chromatography (Hexane:EtOAc, 100:0–80:20 v:v) gave **2ac** as a pale red oil, 69% (192 mg, 0.86 mmol). ^1^H NMR (500 MHz, CDCl_3_) δ 5.19 (ddd, *J* = 8.4, 6.1, 2.6 Hz, 1H), 3.32 (ddd, 1H), 2.21–2.13 (m, 3H), 2.06 (ddd, *J* = 13.0, 8.8, 1.5 Hz, 1H), 1.91–1.80 (m, 2H), 1.78–1.66 (m, 4H), 1.63–1.53 (m, 2H), 1.22 (s, 3H), 1.04 (s, 3H). ^13^C{H} NMR (126 MHz, CDCl_3_) δ 212.0, 169.5, 78.4, 54.6, 53.2, 40.9, 34.6, 32.9, 32.7, 29.1, 27.9, 23.9, 23.8. IR (neat) ν_max_ 2956, 2870, 1752, 1722, 1461, 4371, 1334, 1308, 1260, 1223, 1163, 1182, 1036, 962 cm^–1^. HRMS (ESI-TOF) *m*/*z* [M + Na^+^] calcd for C_13_H_20_O_3_Na 247.1310, found 247.1317.

#### Synthesis of *S*-Phenyl 4,4-dimethyl-2-oxocyclopentane-1-carbothioate (**3a**) and 5,5-Dimethyl-2-(phenylthio)cyclohexane-1,3-dione (**3b**)

General procedure B was followed using diazodimedone (**1a**, 0.208 g, 1.25 mmol), thiophenol (0.152 g, 1.37 mmol) and a temperature of 130 °C. Silica gel chromatography (Hexane:EtOAc, 100:0–80:20 v:v) gave an inseparable mixture of **3a** and **3b** as a pale yellow oil, 56% (169 mg, 0.70 mmol). ^1^H NMR (500 MHz, CDCl_3_) δ 10.98 (s, 1H 3b), 7.49–7.39 (m, 5H 3a), 3.70 (dd, *J* = 10.4, 8.7 Hz, 1H 3a), 2.46 (d, *J* = 1.2 Hz, 2H 3b), 2.39 (d, *J* = 1.3 Hz, 2H 3b), 2.35–2.19 (m, 3H 3a), 2.12 (ddd, *J* = 13.2, 8.7, 1.5 Hz, 1H 3a), 1.23 (s, 3H 3a), 1.19 (s, 6H 3b), 1.07 (s, 3H 3a). ^13^C{H} NMR (101 MHz, CDCl_3_) δ 137.2, 135.6, 132.6, 131.9, 130.8, 129.2, 129.1, 128.9, 128.7, 128.6, 127.7, 127.3, 66.8, 54.9, 53.6, 39.9, 37.5, 37.1, 34.9, 30.6, 29.9, 28.4, 23.1. Data are consistent for **3b** with literature precedent.^22^

#### Synthesis of *S*-Benzyl 4,4-dimethyl-2-oxocyclopentane-1-carbothioate (**3c**) and 2-(Benzylthio)-5,5-dimethylcyclohexane-1,3-dione (**3d**)

General procedure B was followed using diazodimedone (**1a**, 0.208 g, 1.25 mmol), benzyl mercaptan (0.171 g, 1.37 mmol) and a temperature of 130 °C. Silica gel chromatography (Hexane:EtOAc, 100:0–80:20 v:v) gave an inseparable mixture of **3c** and **3d** as a pale yellow oil, 55% (169 mg, 0.69 mmol). ^1^H NMR (500 MHz, CDCl_3_) δ 7.35–7.20 (m, 5H 3c, 5H 3d), 4.21 (d, *J* = 13.8 Hz, 1H 3c), 4.18 (s, 1H 3d), 4.12 (d, *J* = 13.8 Hz, 1H 3c), 3.59 (ddd, *J* = 10.7, 8.6, 0.7 Hz, 1H 3c), 2.96 (s, 1H 3d), 2.88 (s, 1H 3d), 2.37–2.16 (m, 4H 3c), 2.07 (ddd, *J* = 13.1, 8.7, 1.8 Hz, 4H 3d), 1.23 (s, 3H 3c), 1.13 (s, 6H 3d), 1.04 (s, 3H 3c). ^13^C{H} NMR (101 MHz, CDCl_3_) δ 129.6, 129.0, 128.8, 128.6, 127.5, 62.2, 53.5, 43.5, 41.0, 33.9, 29.0, 28.4, 27.9. Data consistent for **3d** with literature precedent.^22^

#### Synthesis of *N*-Butyl-4,4-dimethyl-2-oxocyclopentane-1-carboxamide (**4a**)

General procedure B was followed using diazodimedone (**1a**, 0.208 g, 1.25 mmol), *n*-butylamine (0.101g, 1.37 mmol) and a temperature of 130 °C. Silica gel chromatography (Hexane:EtOAc, 100:0–80:20 v:v) gave **4a** as a pale brown solid, 88% (232 mg, 1.10 mmol). mp 48–51 °C. ^1^H NMR (500 MHz, CDCl_3_) δ 6.74 (br s, 1H), 3.31–3.19 (m, 2H), 3.17 (t, *J* = 9.5 Hz, *1H*), 2.32 (dd, *J* = 13.4, 9.9 Hz, 1H), 2.25–2.13 (m, 2H), 2.08 (m, 1H), 1.49 (m, 2H), 1.39–1.29 (m, 2H), 1.16 (s, 3H), 1.06 (s, 3H), 0.91 (t, *J* = 7.3 Hz, 3H) ppm. ^13^C{H} NMR (126 MHz, CDCl_3_) δ 216.7, 166.7, 53.7, 53.6, 39.4, 39.3, 34.0, 31.6, 28.8, 27.8, 20.1, 13.8 ppm. IR (neat) ν_max_ 3265, 3101, 2933, 2870, 1737, 1640, 1572, 1461, 1357, 1230, 1118, 738 cm^–1^. HRMS (ESI-TOF) *m*/*z* [M + Na^+^] calcd for C_12_H_21_NO_2_Na 234.1470, found 234.1478.

#### Synthesis of *N*-Benzyl-4,4-dimethyl-2-oxocyclopentane-1-carboxamide (**4b**)

General procedure B was followed using diazodimedone (**1a**, 0.208 g, 1.25 mmol), benzyl amine (0.147 g, 1.37 mmol) and a temperature of 130 °C. Silica gel chromatography (Hexane:EtOAc, 100:0–80:20 v:v) gave **4b** as a pale yellow solid, 71% (217 mg, 0.89 mmol). mp 69–71 °C. ^1^H NMR (500 MHz, CDCl_3_) δ 7.29–7.23 (m, 2H), 7.23–7.17 (m, 3H), 7.00 (br s, 1H), 4.45 (dd, *J* = 14.9, 6.0 Hz, 1H), 4.34 (dd, *J* = 14.8, 5.6 Hz, 1H), 3.17 (t, *J* = 9.6 Hz, 1H), 2.29 (dd, *J* = 13.4, 9.9, 1H), 2.20–1.93 (m, 3H), 1.11 (s, 3H), 1.01 (s, 3H) ppm. ^13^C{H} NMR (126 MHz, CDCl_3_) δ 216.3, 166.8, 138.1, 128.7, 127.7, 127.4, 53.7, 53.7, 43.7, 39.3, 34.0, 28.8, 27.8 ppm. IR (neat) ν_max_ 3343, 2960, 2870, 1774, 1737, 1703, 1640, 1569, 1361, 1167, 1126, 1055, 995, 902, 745, 719, 686 cm^–1^. HRMS (ESI-TOF) *m*/*z* [M + H^+^] calcd for C_15_H_20_NO_2_ 246.1494, found 246.1501.

#### Synthesis of 4,4-Dimethyl-2-(piperidine-1-carbonyl)cyclopentane-1-one (**4c**)

General procedure B was followed using diazodimedone (**1a**, 0.208 g, 1.25 mmol), piperidine (0.106 g, 1.37 mmol) and a temperature of 130 °C. Silica gel chromatography (Hexane:EtOAc, 100:0–80:20 v:v) gave **4c** as a white solid, 87% (243 mg, 1.09 mmol). mp 78–81 °C. ^1^H NMR (500 MHz, CDCl_3_) δ 3.69–3.53 (m, 1H), 3.52–3.36 (m, 1H), 2.42 (t, *J* = 10.6 Hz, 1H), 2.24–2.07 (m, 1H), 1.91 (ddd, *J* = 13.0, 8.6, 2.2, 1H), 1.78–1.44 (m, 2H), 1.21 (s, 1H), 1.02 (s, 1H) ppm. ^13^C{H} NMR (126 MHz, CDCl_3_) δ 213.8, 166.8, 53.5, 50.9, 47.3, 43.4, 40.8, 34.2, 29.1, 28.1, 26.6, 25.6, 24.6 ppm. IR (neat) ν_max_ 2941, 2855, 1730, 1618, 1439, 1230, 1122, 1006 cm^–1^. HRMS (ESI-TOF) *m*/*z* [M + H^+^] calcd for C_13_H_22_NO_2_ 224.1651, found 224.1659.

#### Synthesis of *N*,*N*-Bis(4-(*tert*-butyl)phenyl)-4,4-dimethyl-2-oxocyclopentane-1-carboxamide (**4d**)

General procedure B was followed using diazodimedone (**1a**, 0.208 g, 1.25 mmol), *N*,*N*-bis(4-(*tert*-butyl)phenyl)amine (0.386 g, 1.37 mmol) and a temperature of 130 °C. Silica gel chromatography (Hexane:EtOAc, 100:0–80:20 v:v) gave **4d** as a yellow semi solid, 74% (388 mg, 0.93 mmol). ^1^H NMR (500 MHz, CDCl_3_) δ 7.40 (d, *J* = 8.1 Hz 2H), 7.31 (m, 4H), 7.18 (d, *J* = 8.3 Hz, 2H), 3.52 (t, *J* = 9.6 Hz, 1H), 2.42–2.33 (m, 2H), 2.18 (m, 1H), 1.99–1.91 (m, 1H), 1.32 (s, 9H), 1.27 (s, 9H), 1.22 (s, 3H), 0.88 (s, 3H) ppm. ^13^C{H} NMR (126 MHz, CDCl_3_) δ 213.9, 170.5, 151.1, 149.1, 140.0, 139.9, 128.1, 126.6, 126.0, 125.8, 53.6, 52.8, 42.1, 34.7, 34.5, 34.5, 31.3, 29.1, 28.0 ppm. IR (neat) ν_max_ 3373, 2956, 2904, 2866, 1737, 1607, 1513, 1461, 1316, 1189, 820 cm^–1^. HRMS (ESI-TOF) *m*/*z* [M + H^+^] calcd for C_28_H_38_NO_2_ 420.2903, found 420.2908.

#### Synthesis of 4,4-Dimethyl-2-oxo-*N*-(prop-2-yn-1-yl)cyclopentanecarboxamide (**4e**)

General procedure B was followed using diazodimedone (**1a**, 0.208 g, 1.25 mmol), propargyl amine (0.075g, 1.37 mmol) and a temperature of 130 °C. Silica gel chromatography (Hexane:EtOAc, 100:0–80:20 v:v) gave **4e** as a pale yellow solid, 81% (98 mg, 1.01 mmol). mp 94–98 °C. ^1^H NMR (400 MHz, CDCl_3_) δ 7.02 (s, 1H), 4,08–3,92 (m, 2H), 3.19 (t, *J* = 9.6 Hz, 1H), 2.29–2.02 (m, 5H), 1.12 (s, 3H), 1.03 (s, 3H). ^13^C{H} NMR (100 MHz, CDCl_3_) δ 215.7, 166.7, 79.4, 71.5, 53.6, 53.5, 39.2, 34.0, 29.2, 28.8, 27.8. IR (neat) *v*_max_ 3257, 2955, 1733, 1640, 1550, 1341, 1129 cm^–1^. HRMS (ESI-TOF) *m*/*z* [M + Na^+^] calcd for C_11_H_15_NO_2_Na 216.1001, found 216.1002.

#### Synthesis of 4,4-Dimethyl-2-oxo-*N*-phenylcyclopentane-1-carboxamide (**4f**)

General procedure B was followed using diazodimedone (**1a**, 0.208 g, 1.25 mmol), aniline (0.128 g, 1.37 mmol) and a temperature of 130 °C. Silica gel chromatography (Hexane:EtOAc, 100:0–80:20 v:v) gave **4f** as a pale pink sweet smelling solid, 78% (225 mg, 0.98 mmol). mp 107–110 °C. ^1^H NMR (500 MHz, CDCl_3_) δ 8.74 (s, 1H), 7.54 (d, *J* = 7.3 Hz, 2H), 7.32 (t, *J* = 7.98 Hz, 2H), 7.10 (t, *J* = 7.4 Hz, jf1H), 3.39 (t, *J* = 9.5 Hz, 1H), 2.39 (dd, *J* = 13.4, 9.8 Hz, 1H), 2.27 (q, *J* = 18.1 Hz, j2H), 2.17 (ddd, *J* = 13.5, 9.2, 1.7 Hz, 1H), 1.20 (s, 3H), 1.12 (s, 3H) ppm. ^13^C{H} NMR (126 MHz, CDCl_3_) δ 216.7, 164.7, 137.7, 129.0, 124.3, 119.8, 54.2, 53.8, 39.0, 34.0, 28.8, 27.9 ppm. IR (neat) ν_max_ 3291, 3071, 2956, 2930, 2870, 1741, 1640, 1546, 1357, 1038, 1238, 1118, 719 cm^–1^. HRMS (ESI-TOF) *m*/*z* [M + Na^+^] calcd for C_14_H_17_NO_2_Na 254.1157, found 254.1158.

#### Synthesis of *N*-Cyclopropyl-4,4-dimethyl-2-oxocyclopentanecarboxamide (**4g**)

General procedure B was followed using diazodimedone (**1a**, 0.208 g, 1.25 mmol), cyclopropylamine (0.078 g, 1.37 mmol) and a temperature of 130 °C. Silica gel chromatography (Hexane:EtOAc, 100:0–80:20 v:v) gave **4g** as a white solid, 38% (88 mg, 0.48 mmol, 250 psi) and 68% (166 mg, 0.85 mmol, 500 psi). mp 97–101 °C. ^1^H NMR (400 MHz, CDCl_3_) δ 6.80 (s, 1H), 3.12 (t, *J* = 9.5 Hz, 1H), 2.69 (tq, *J* = 7.2, 3.6 Hz, 1H), 2.31 (dd, *J* = 13.3, 9.5 Hz, 1H), 2.23–2.11 (m, 2H), 2.05 (ddd, *J* = 13.3, 9.5, 1.4 Hz, 1H), 1.15 (s, 3H), 1.05 (s, 3H), 0.80–0.68 (m, 2H), 0.55–0.43 (m, 2H). ^13^C{H} NMR (100 MHz, CDCl_3_) δ 216.4, 168.2, 53.7, 39.2, 34.0, 28.8, 27.9, 22.7, 6.6, 6.5. IR (neat) *v*_max_ 3295, 2955, 2870, 1744, 1640, 1539, 1320, 1125 cm^–1^. HRMS (ESI-TOF) *m*/*z* [M + Na^+^] calcd for C_11_H_17_NO_2_Na 218.1157, found 218.1153.

#### Synthesis of *N*-Cyclobutyl-4,4-dimethyl-2-oxocyclopentanecarboxamide (**4h**)

General procedure B was followed using diazodimedone (**1a**, 0.208 g, 1.25 mmol), cyclobutylamine (0.096 g, 1.37 mmol) and a temperature of 130 °C. Silica gel chromatography (Hexane:EtOAc, 100:0–80:20 v:v) gave **4h** as a white solid, 19% (50 mg, 0.24 mmol, 250 psi) and 69% (180 mg, 0.86 mmol, 500 psi). mp 93–97 °C. ^1^H NMR (400 MHz, CDCl_3_) δ 6.86 (br s, 1H), 4.34 (sext, *J* = 8 Hz, 1H), 3.12 (t, *J* = 9.6 Hz, 1H), 2.36–2.25 (m, 3H), 2.24–2.12 (m, 2H), 2.04 (dd, *J* = 13.1, 9.6 Hz, 1H), 1.87 (tt, *J* = 19.8, 9.6 Hz, 2H), 1.76–1.57 (m, 2H), 1.14 (s, 3H), 1.04 (s, 3H). ^13^C{H} NMR (100 MHz, CDCl_3_) δ 216.4, 165.8, 53.8, 53.6, 44.9, 39.3, 34.0, 31.2, 31.0, 28.8, 27.9, 15.2. IR (neat) *v*_max_ 3287, 2937, 2870, 1736, 1632, 1546, 1241, 1121 cm^–1^. HRMS (ESI-TOF) *m*/*z* [M + Na^+^] calcd for C_12_H_19_NO_2_Na 232.1313, found 232.1307.

#### Synthesis of *N*-((3s,5s,7s)-Adamantan-1-yl)-4,4-dimethyl-2-oxocylopentanecarboxamide (**4i**)

General procedure B was followed using diazodimedone (**1a**, 0.208 g, 1.25 mmol), (3s, 5s, 7s)adamantan-1-amine (0.207 g, 1.37 mmol) and a temperature of 130 °C. Silica gel chromatography (Hexane:EtOAc, 100:0–80:20 v:v) gave **4i** as a white solid, 69% (126 mg, 0.86 mmol). mp 127–132 °C. ^1^H NMR (400 MHz, CDCl_3_) δ 6.43 (br s, 1H), 3.12 (t, *J* = 9.4 Hz, 1H), 2.30 (dd, *J* = 13.4, 9.4 Hz, 1H), 2.18 (s, 2H), 2.06 (br s, 3H), 2.00–1.99 (m, 7H), 1.66–1.63 (m, 6H), 1.14 (s, 3H), 1.05 (s, 3H). ^13^C{H} NMR (100 MHz, CDCl_3_) δ 216.8, 165.5, 54.5, 53.9, 52.0, 41.6, 39.2, 36.5, 34.0, 29.5, 28.8, 28.0. IR (neat) *v*_max_ 3309, 2907, 2851, 1744, 1636, 1543, 1356, 1308, 1125 cm^–1^. HRMS (ESI-TOF) *m*/*z* [M + H^+^] calcd for C_18_H_28_NO_2_ 290.2120, found 290.2116.

#### Large Scale Continuous Flow Synthesis of Benzyl 4,4-dimethyl-2-oxocyclopentane-1-carboxylate (**2ab**) and *N*-Cyclopropyl-4,4-dimethyl-2-oxocyclopentanecarboxamide (**4g**)

Diazodimedone (**1a**, 9.0 g, 54.2 mmol) and benzyl alcohol (6.44 g, 59.6 mmol) or cyclopropylamine (3.40 g, 59.6 mmol) were added to a round-bottom flask in toluene (0.25 M). The solution was continuously pumped directly through an HPLC pump at 0.5 mL min^–1^ and into a 20 mL stainless steel reactor coil at 130 °C for 6 h. The eluting stream was collected and purified by silica gel column chromatography, which gave **2ab** as a pale yellow oil, 85% (9.36 g, 6.3 mmol h^–1^, 250 PSI) or **4g** as a white solid, 80% (7.02 g, 6.0 mmol h^–1^, 500 PSI).

#### General Procedure C for the Synthesis of 1,3-Oxazine-2,4-diones

Diazodimedone (0.208 g, 1.25 mmol) and the corresponding isocyanate (1.37 mmol, 1.1 equiv) were mixed in toluene (5 mL, 0.25 M) and loaded into a 5 mL loading loop. The loop was injected into a stream of toluene at 0.5 mL min^–1^ and heated to 130 °C for 40 min using a 20 mL stainless steel coil reactor loaded onto a CRD Polar Bear device. The products were purified by silica gel column chromatography using Hexane/EtOAc (100:0–60:40 v:v) as solvent elution.

#### Synthesis of 3-Ethyl-6,6-dimethyl-6,7-dihydrocyclopenta[*e*][1,3]oxazine-2,4(3*H*,5*H*)-dione (**5a**)

General procedure C was followed using ethyl isocyanate (0.098 g, 1.37 mmol). Silica gel chromatography (Hexane:EtOAc, 100:0–60:40 v:v) gave **5a** as a yellow oil, 71% (186 mg, 0.89 mmol). ^1^H NMR (500 MHz, CDCl_3_) δ 3.94 (q, *J* = 7.1 Hz, 2H), 2.57 (app. s, 2H), 2.48 (app. s, 2H), 1.24 (t, *J* = 7.1 Hz, 2H), 1.21 (s, 3H) ppm. ^13^C{H} NMR (126 MHz, CDCl_3_) δ 164.9, 159.9, 149.8, 110.9, 45.6, 40.6, 37.4, 36.5, 29.8, 1.7. IR (neat) ν_max_ 2960, 2870, 1759, 1692, 1439, 1308, 1241, 1170, 1107, 1036, 958, 760 cm^–1^. HRMS (ESI-TOF) *m*/*z* [M + Na^+^] calcd for C_11_H_15_NO_3_Na 232.0950, found 232.0960.

#### Synthesis of 6,6-Dimethyl-3-phenyl-6,7-dihydrocyclopenta[*e*][1,3]oxazine-2,4(3*H*,5*H*)-dione (**5b**)

General procedure C was followed using phenyl isocyanate (0.164 g, 1.37 mmol). Silica gel chromatography (Hexane:EtOAc, 100:0–60:40 v:v) gave **5b** as a pale brown solid, 36% (116 mg, 0.45 mmol). mp 148–151 °C. ^1^H NMR (500 MHz, CDCl_3_) δ 7.50 (m, 2H), 7.45 (m, 1H), 7.24 (m, 2H), 2.67 (app. s, 2H), 2.55 (app. s, 2H), 1.26 (s, 6H). ^13^C{H} NMR (126 MHz, CDCl_3_) δ 165.6, 159.9, 149.8, 134.3, 129.6, 129.2, 128.1, 111.2, 45.7, 40.7, 36.6, 29.9 ppm. IR (neat) ν_max_ 2956, 2866, 1759, 1692, 1409, 1342, 1245, 1144, 697 cm^–1^. HRMS (ESI-TOF) *m*/*z* [M + Na^+^] calcd for C_15_H_15_NO_3_Na 280.0950, found 280.0950.

#### Synthesis of 3-(3-Acetylphenyl)-6,6-dimethyl-6,7-dihydrocyclopenta[*e*][1,3]-oxazine-2,4(3*H*,5*H*)-dione (**5c**)

General procedure C was followed using 3-acetylphenyl isocyanate (0.222 g, 1.37 mmol). Silica gel chromatography (Hexane:EtOAc, 100:0–60:40 v:v) gave **5c** as a white solid, 47% (172 mg, 0.59 mmol). mp 172–175 °C ^1^H NMR (500 MHz, CDCl_3_) δ 8.06 (dt, *J* = 7.9, 1.4 Hz, 1H), 7.87 (t, *J* = 1.9 Hz, 1H), 7.63 (t, *J* = 7.9 Hz 1H), 7.50–7.45 (ddd, *J* = 7.8, 2.1, 1.1 Hz, 1H), 2.69 (t, *J* = 1.8 Hz, 2H), 2.62 (s, 3H), 2.57 (t, *J* = 1.8 Hz, 2H), 1.28 (s, 6H). ^13^C{H} NMR (126 MHz, CDCl_3_) δ 196.7, 166.0, 159.8, 149.7, 138.5, 134.9, 132.9, 129.9, 129.1, 128.3, 111.2, 45.8, 40.7, 36.8, 29.9, 26.7. IR (neat) ν_max_ 3280, 1677, 1625, 1595, 1562, 1439, 1424, 1357, 1275, 1226, 1200, 1092, 976, 902, 793, 749, 686 cm^–1^. HRMS (ESI-TOF) *m*/*z* [M + Na^+^] calcd for C_17_H_17_NO_4_Na 322.1055, found 322.1053.

#### Synthesis of 3-(4-Chlorophenyl)-6,6-dimethyl-6,7-dihydrocyclopenta[*e*][1,3]-oxazine-2,4(3*H*,5*H*)-dione (**5d**)

General procedure C was followed using 4-chlorophenyl isocyanate (0.256 g, 1.37 mmol). Silica gel chromatography (Hexane:EtOAc, 100:0–60:40 v:v) gave **5d** as a pale pink solid, 46% (167 mg, 0.58 mmol). ^1^H NMR (500 MHz, CDCl_3_) δ 7.47 (d, *J* = 8.6 Hz, 2H), 7.19 (d, *J* = 8.6 Hz, 1H), 2.67 (t, *J* = 1.8 Hz, 2H), 2.55 (t, *J* = 1.8 Hz, 2H), 1.26 (s, 3H). ^13^C{H} NMR (126 MHz, CDCl_3_) δ 165.9, 159.8, 149.7, 135.3, 132.8, 130.0, 129.6, 111.2, 45.8, 40.8, 36.8, 30.0. IR (neat) ν_max_ 2956, 2866, 1767, 1692, 1416, 1148, 1081, 757 cm^–1^. mp 168–173 °C HRMS (ESI-TOF) *m*/*z* [M + Na^+^] calcd for C_15_H_14_ClNO_3_Na 314.0560, found 314.0557.

#### Synthesis of 3-(4-Methoxyphenyl)-6,6-dimethyl-6,7-dihydrocyclopenta[*e*][1,3]oxazine-2,4(3*H*,5*H*)-dione (**5e**)

General procedure C was followed using 4-methoxyphenyl isocyanate (0.205 g, 1.37 mmol). Silica gel chromatography (Hexane:EtOAc, 100:0–60:40 v:v) gave **5e** as a pale yellow solid, 38% (136 mg, 0.48 mmol). mp 178–181 °C. ^1^H NMR (500 MHz, CDCl_3_) δ 7.12–7.07 (m, 2H), 6.96–6.91 (m, 2H), 3.78 (s, 3H), 2.60 (app. s, 2H), 2.49 (app. s, 2H), 1.20 (s, 6H). ^13^C{H} NMR (126 MHz, CDCl_3_) δ 165.6, 160.3, 160.0, 150.2, 129.2, 126.9, 115.0, 111.3, 55.6, 45.8, 40.9, 36.8, 30.0. IR (neat) ν_max_ 2956, 2863, 1767, 1696, 1513, 1409, 1342, 1297, 1245, 1144, 1029, 831, 752, 667 cm^–1^. HRMS (ESI-TOF) *m*/*z* [M + Na^+^] calcd for C_16_H_17_NO_4_Na 310.1055, found 310.1049.

#### Synthesis of Phenyl Acyl Azide (**6**)

Benzoyl chloride (1.16 mL, 10 mmol) and tetrabutylammonium iodide (0.01 mmol) were dispersed in CH_2_Cl_2_ (40 mL) and cooled to 0 °C. Sodium azide (975 mg, 15 mmol) solution in water (8 mL) was added portion wise, and the reaction mixture was allowed to return to room temperature and stir overnight. The reaction mixture was diluted with water (32 mL) and the organic phase extracted. The aqueous phase was further extracted with CH_2_Cl_2_ (2 × 40 mL) and the combined organic phases were dried over MgSO_4_ and concentrated in vacuo. The crude mixture was purified using column chromatography (Petroleum Ether:EtOAc 100:0–80:20, 40–60 °C) to give **6** as a white crystalline solid, 86% (1.26 g, 8.6 mmol). ^1^H NMR (400 MHz, CDCl_3_) δ 7.42–7.46 (m, 2H), 7.59–7.63 (m, 1H), 8.02 (d, 2H), ^13^C{H} NMR (126 MHz, CDCl_3_) δ 128.4, 129.2, 130.4, 134.4, 172.5. IR (neat) ν_max_ 2356, 2173, 2132, 1684, 1595, 1446, 1315, 1234, 1182, 984, 678 cm^–1^. Data consistent with literature.^23^

#### Synthesis of 6,6-Dimethyl-3-phenyl-6,7-dihydrocyclopenta[*e*][1,3]oxazine-2,4(3*H*,5*H*)-dione (**5b**) from Diazodimedone, **1a**, and Phenyl Acyl Azide, **6**

Diazodimedione (**1a**, 0.166g, 1 mmol) and phenyl acyl azide (**6**, 0.736 g, 5 mmol) were mixed in toluene (0.2 M) and loaded into a 5 mL loading loop. The solution was injected into a stream of toluene at 0.5 mL min^–1^ and passed through a 20 mL stainless steel reactor at 130 °C, which gave **5b** as a pale brown solid, 64% (165 mg, 0.64 mmol).

## Supporting Information Available

Flow equipment set up, TGA curves, ^1^H NMR and ^13^C{H} NMR spectra (PDF)
