# Supplementary material for: Continuous Flow Generation of Acylketene Intermediates via Nitrogen Extrusion
Source: J Org Chem. 2022 Sep 1;87(18):12297–305. doi: 10.1021/acs.joc.2c01486 (PMC9486939; doi:10.1021/acs.joc.2c01486)
Supplement: Supplementary file 1 — jo2c01486_si_001.pdf [file jo2c01486_si_001.pdf]

Supporting information for

Continuous Flow Generation of Acylketene Intermediates via Nitrogen Extrusion

Harry R. Smallman,[a] Guilherme A. Brancaglion,[ab] Julio C. Pastre[b] and Duncan L. Browne.[a*]

[a] School of Pharmacy, University College London (UCL), 29-39 Brunswick Square, London WC1N 1AX.

[b] Institute of Chemistry, University of Campinas – UNICAMP, PO Box 6154 – Zip Code 13083-970, Campinas, SP, Brazil.

* Corresponding author: Duncan L. Browne, Email: duncan.browne@ucl.ac.uk

Contents

1. Flow chemistry equipment S2
2. TGA Curves S3
3. NMR Spectra S7

1. Flow chemistry equipment

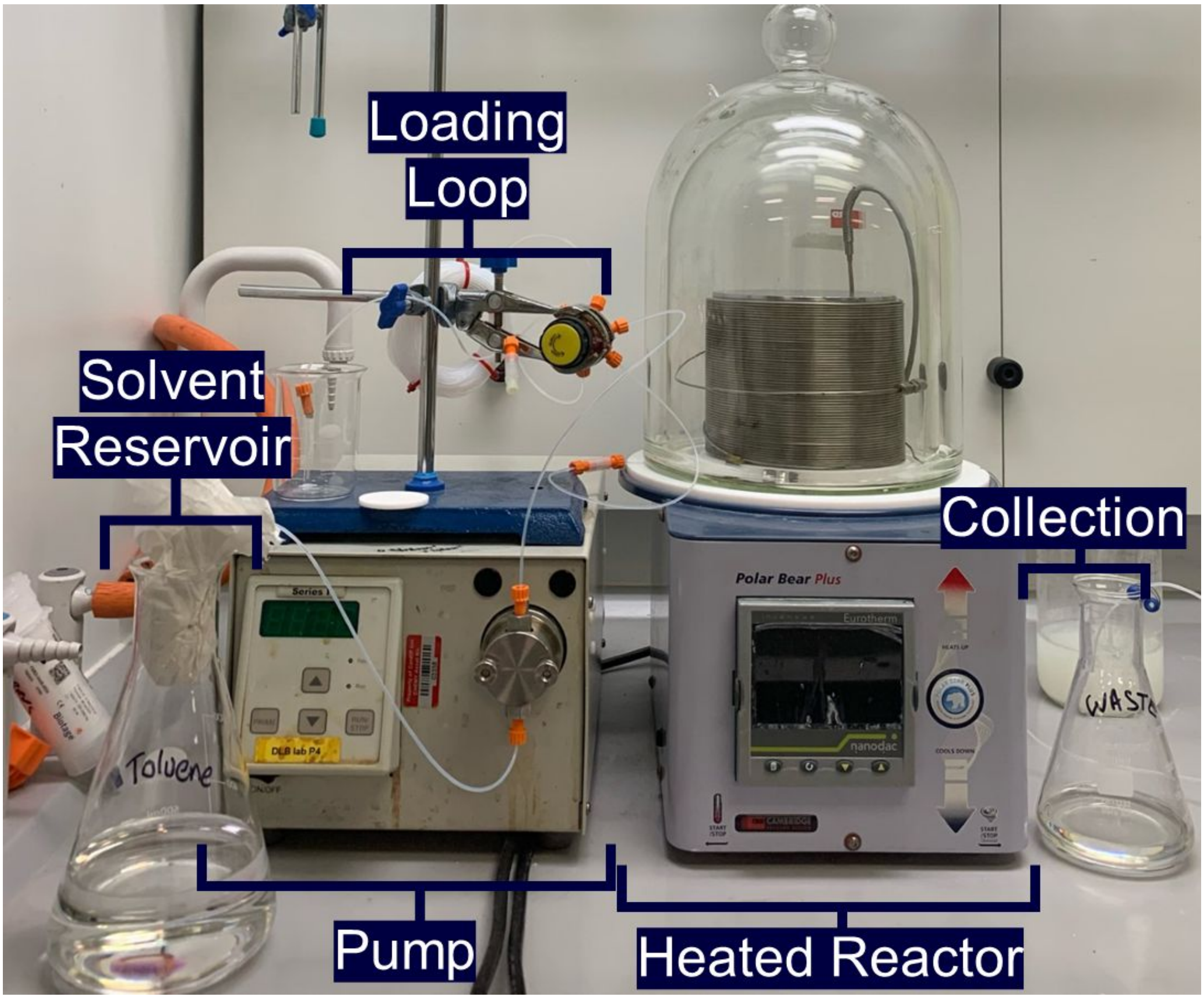

Figure S1. Flow equipment setup

2. TGA Curves

Diazodimedone (1a)

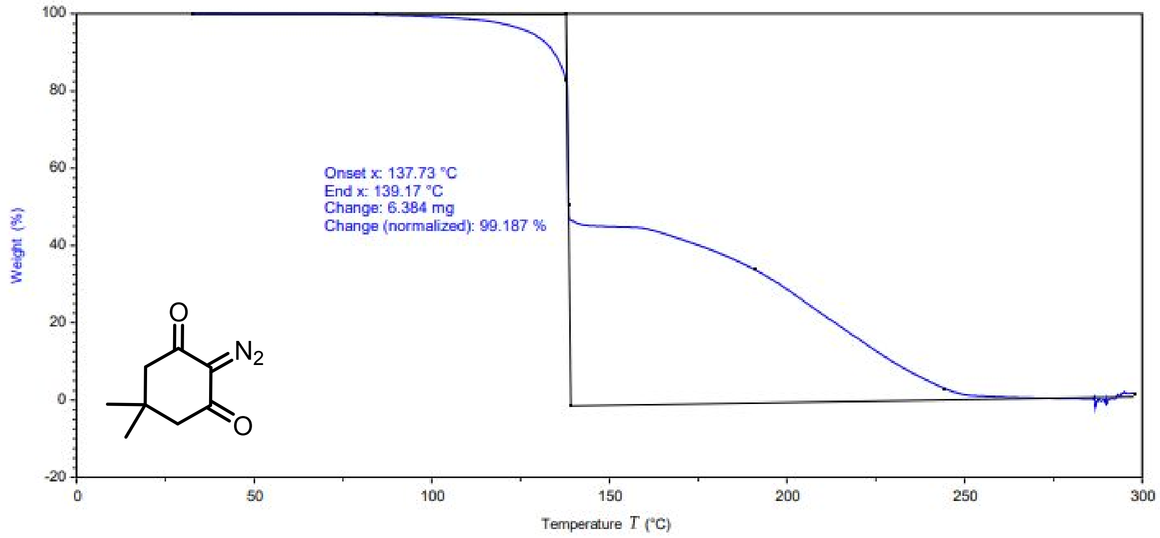

2-diazocyclohexane-1,3-dione (1b)

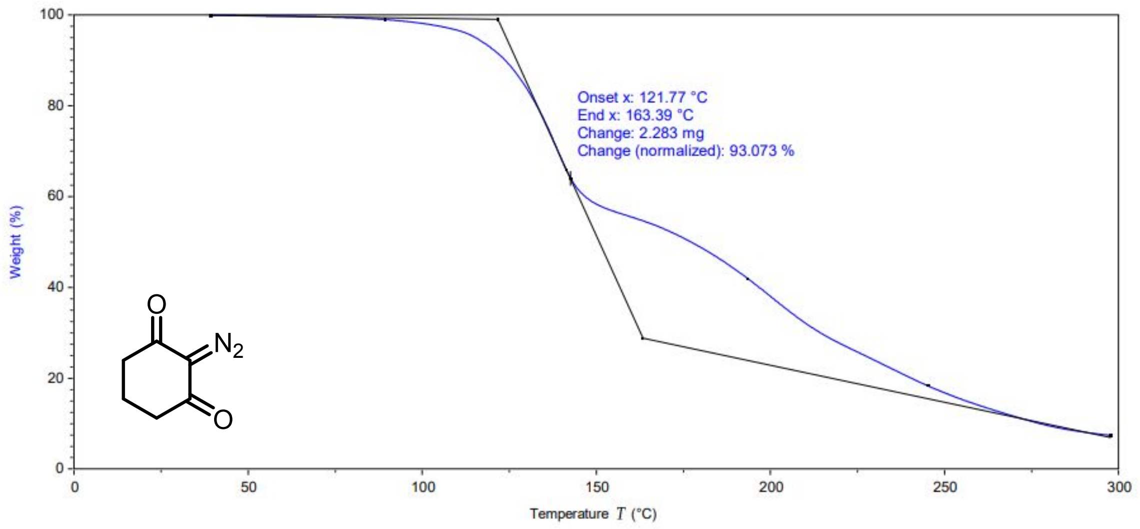

2-diazocycloheptane-1,3-dione (1c)

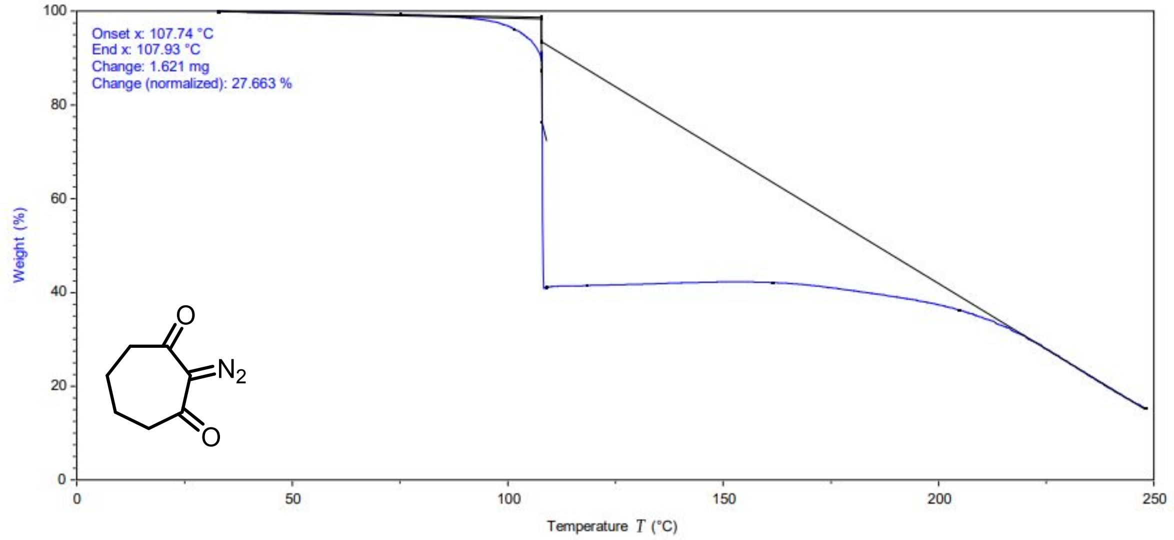

2-diazo-5-phenylcyclohexane-1,3-dione (1d)

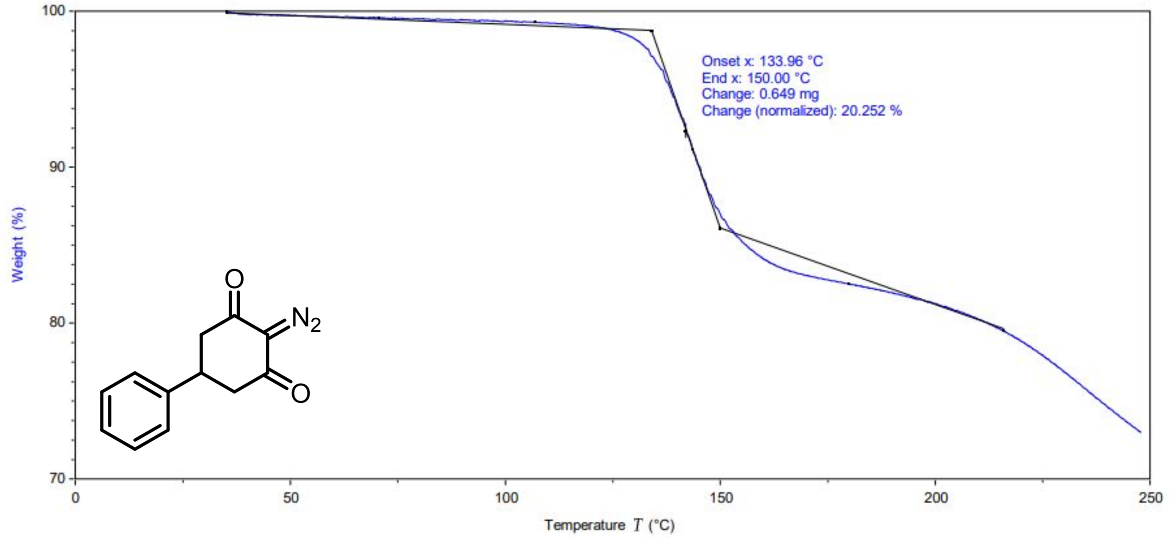

2-diazo-4,4-dimethylcyclohexane-1,3-dione (1e)

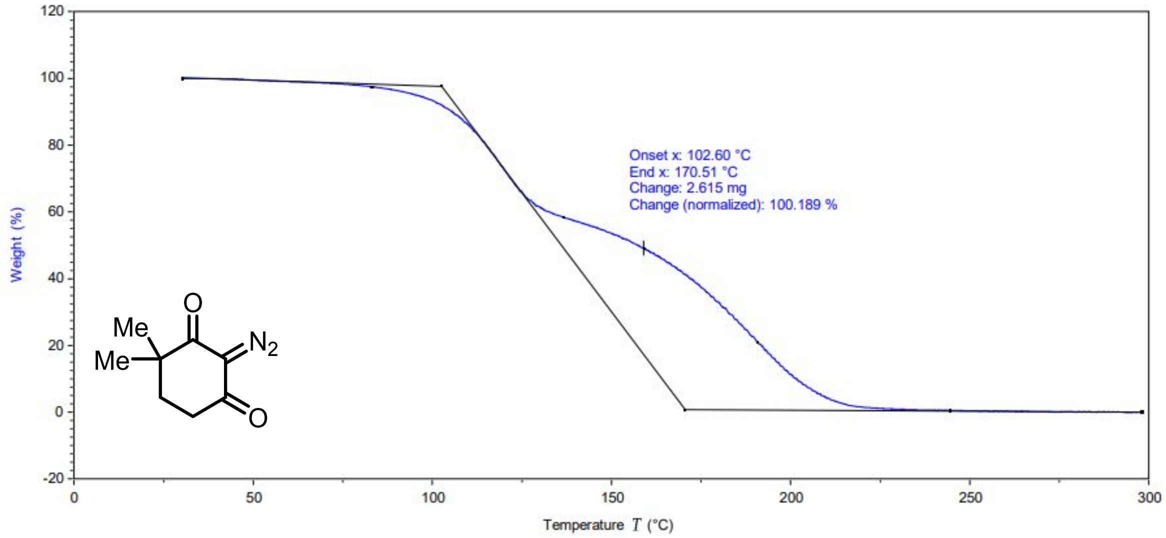

3-diazopentane-2,4-dione (1e)

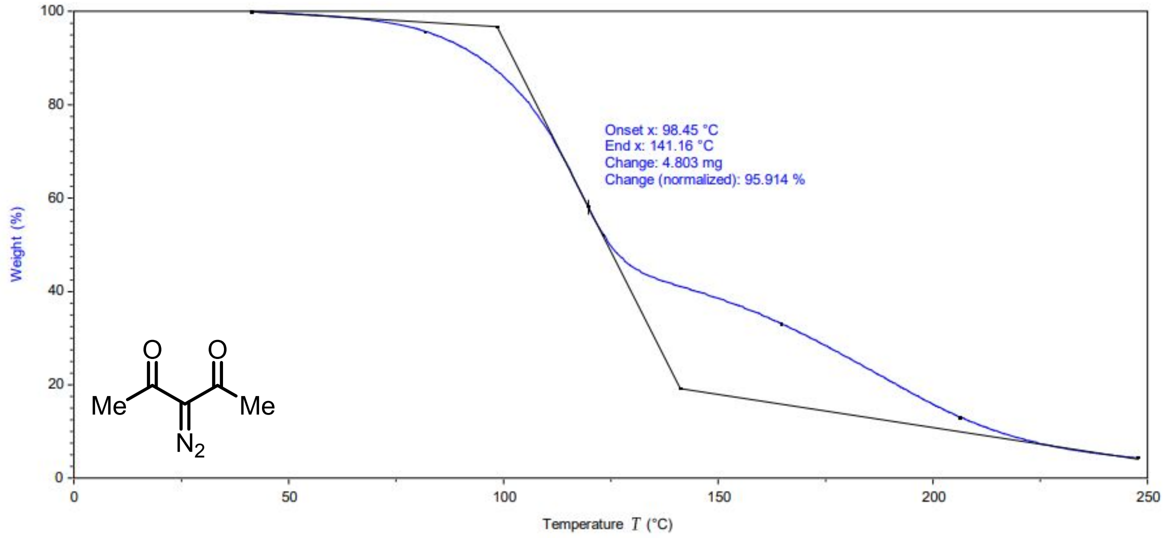

2-diazocyclopentane-1,3-dione (1f)

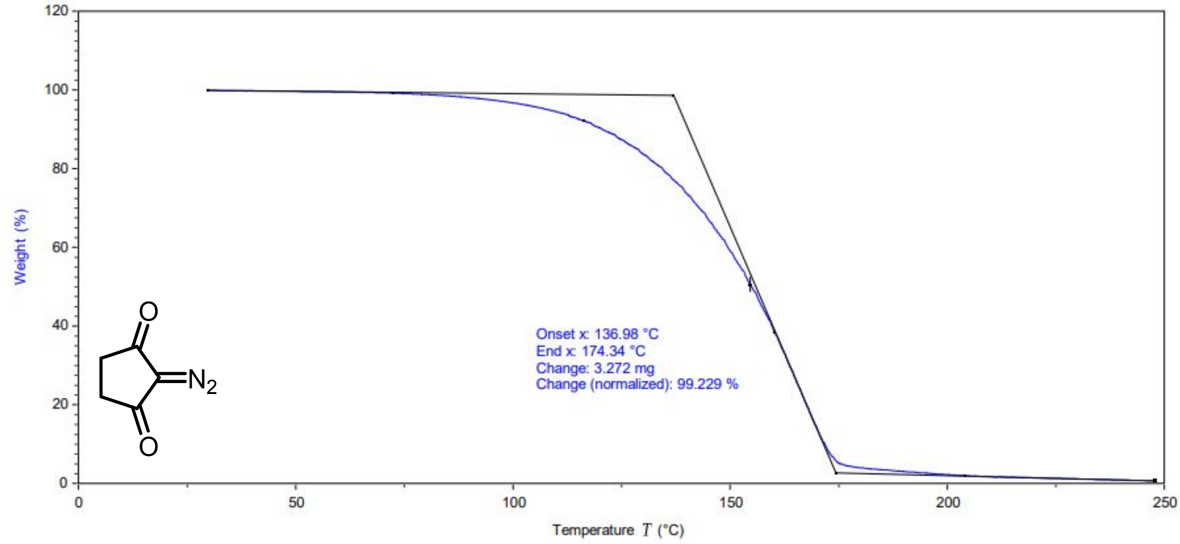

3. NMR Spectra

Diazodimedone (1a)

^1H NMR (400 MHz, $CDCl_3$)

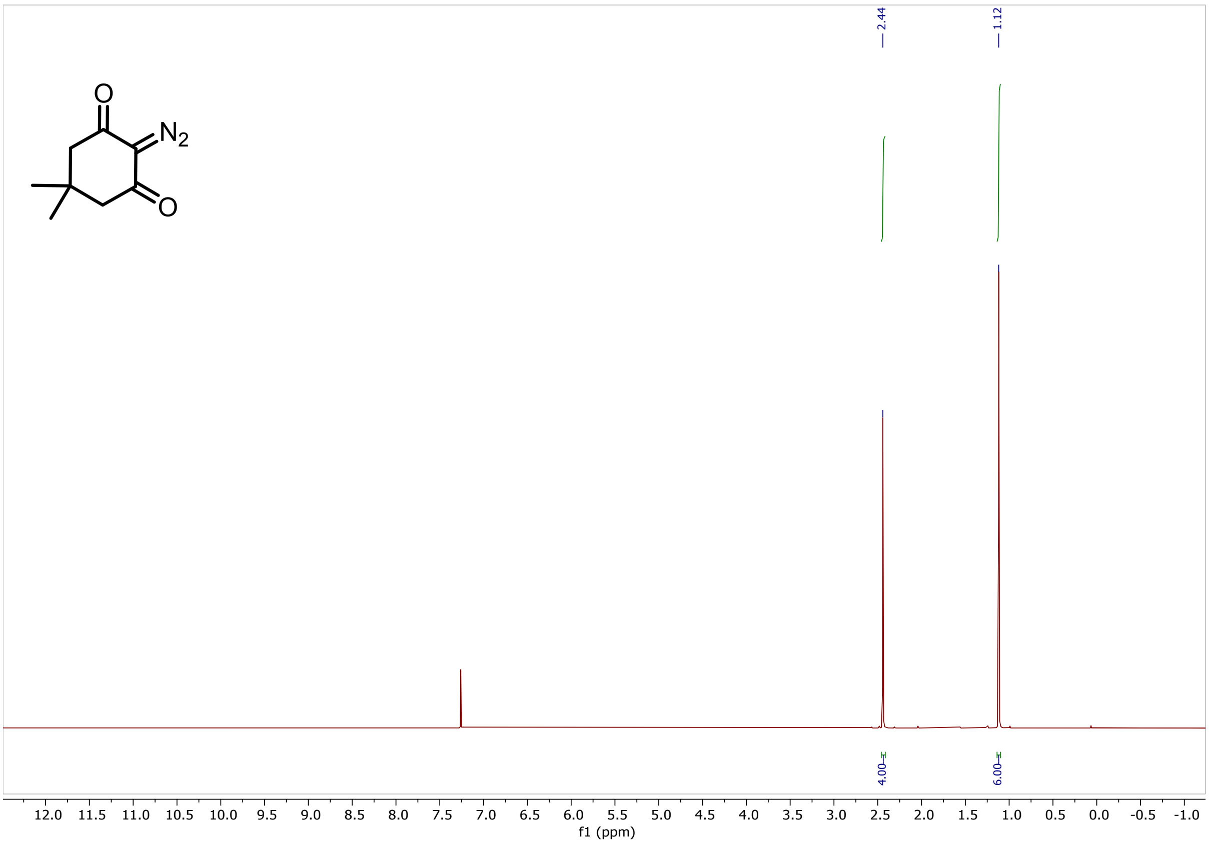

13C{1H} NMR (126 MHz, CDCl3)

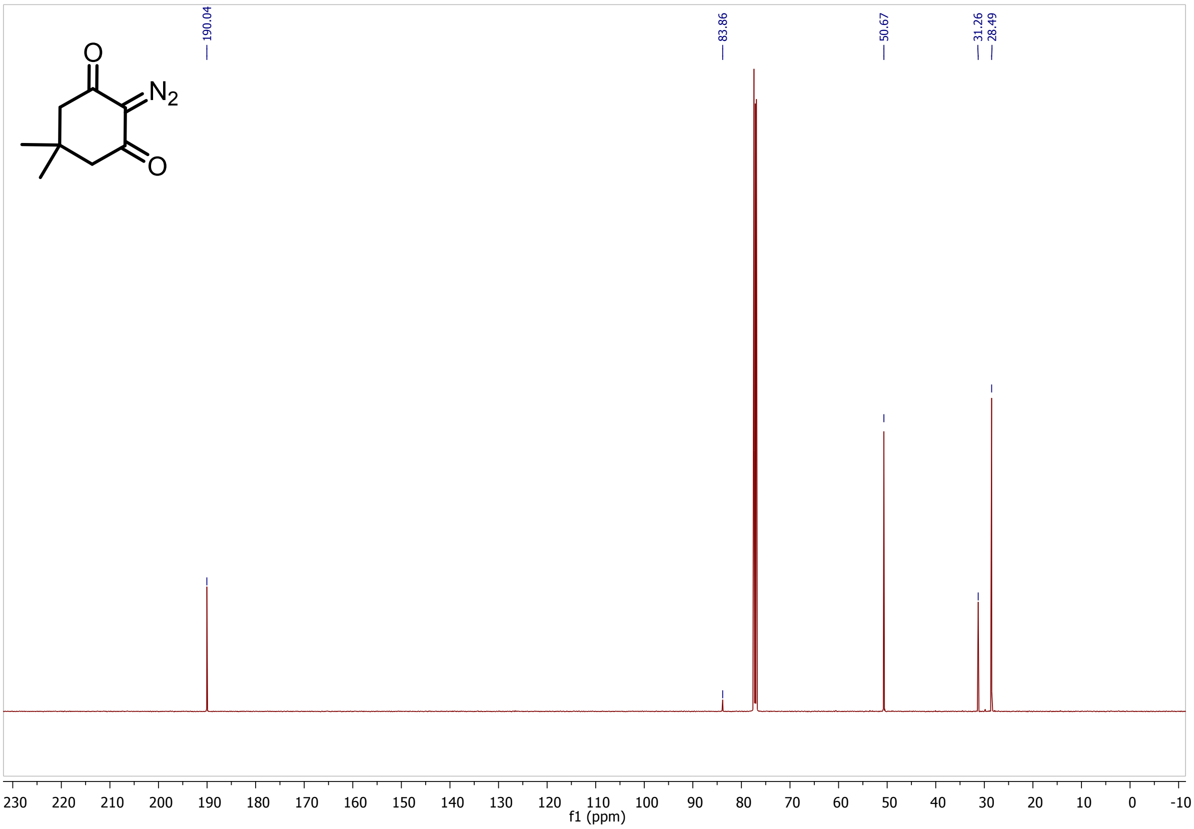

2-diazocyclohexane-1,3-dione (1b)

^1H NMR (400 MHz, $CDCl_3$)

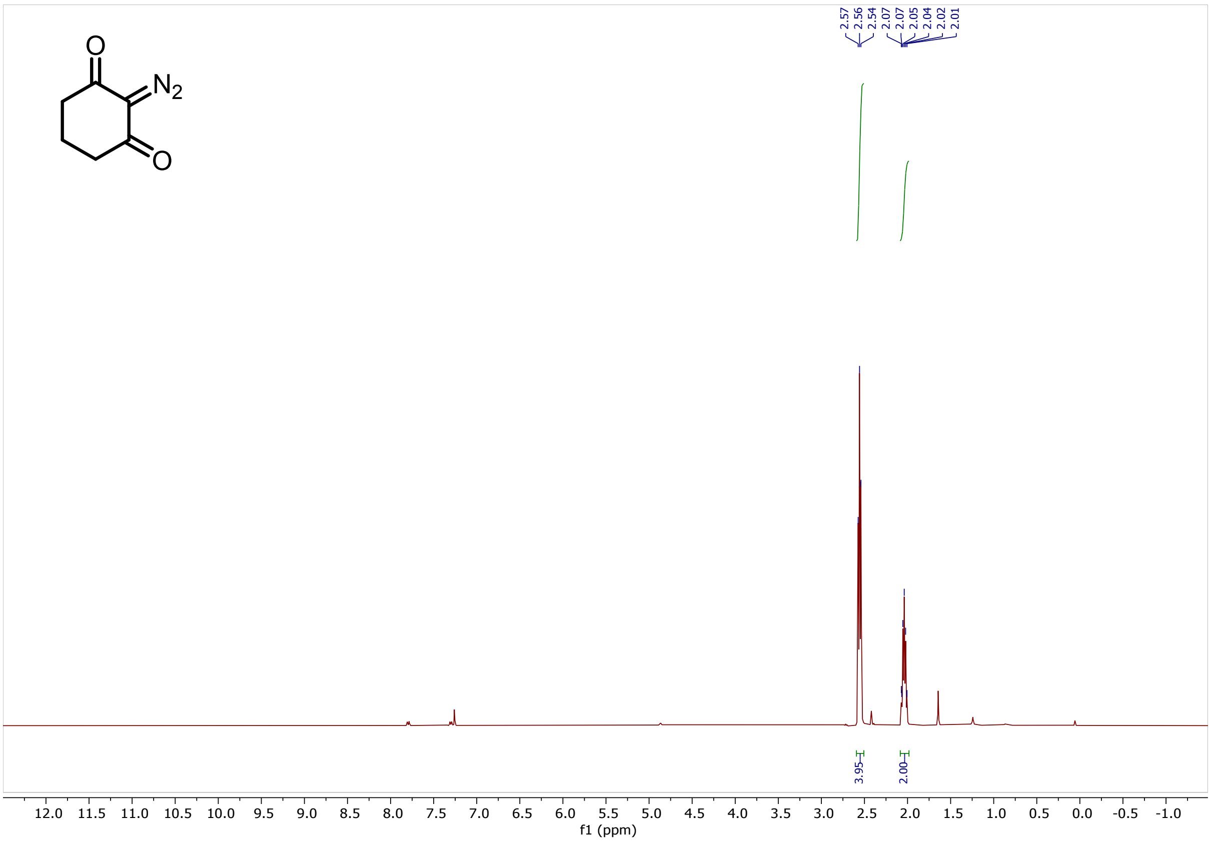

13C{1H} NMR (100 MHz, CDCl3)

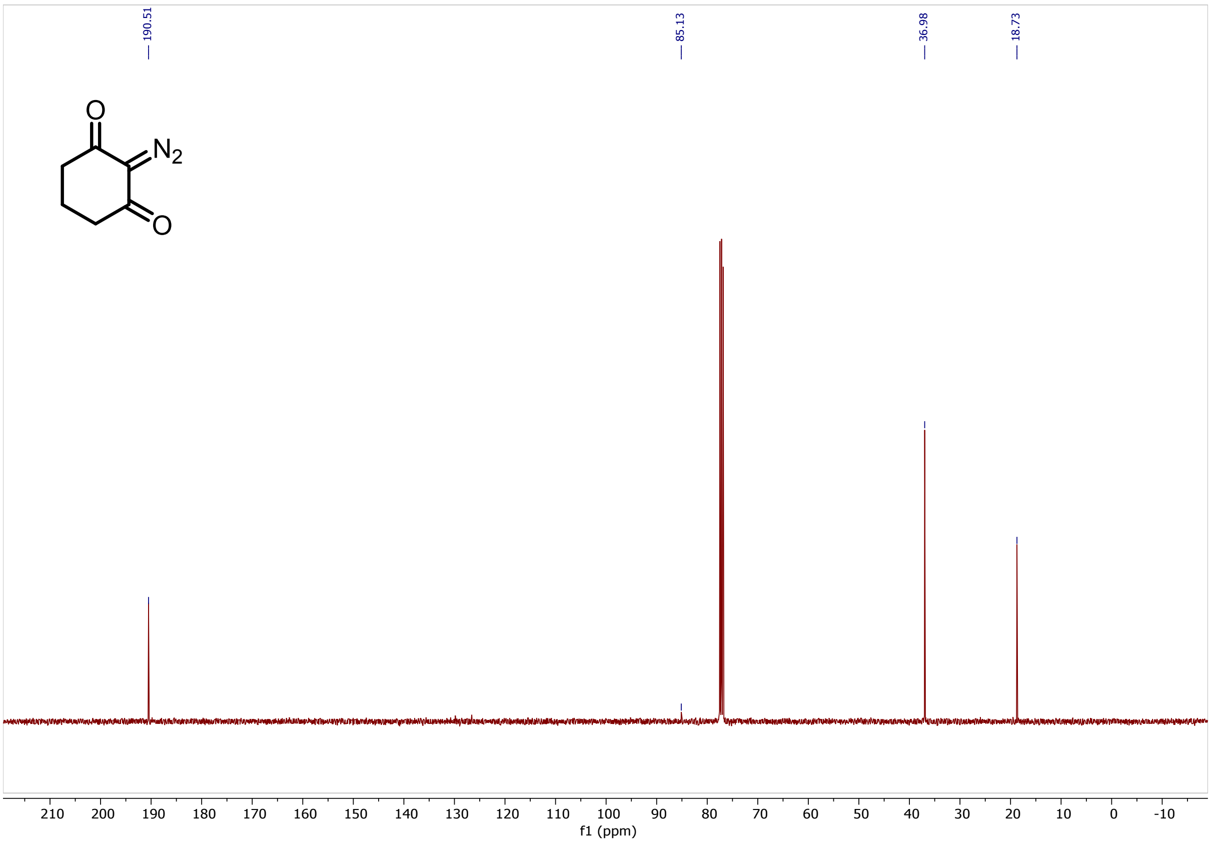

2-diazocycloheptane-1,3-dione (1c)

^1H NMR (400 MHz, $CDCl_3$)

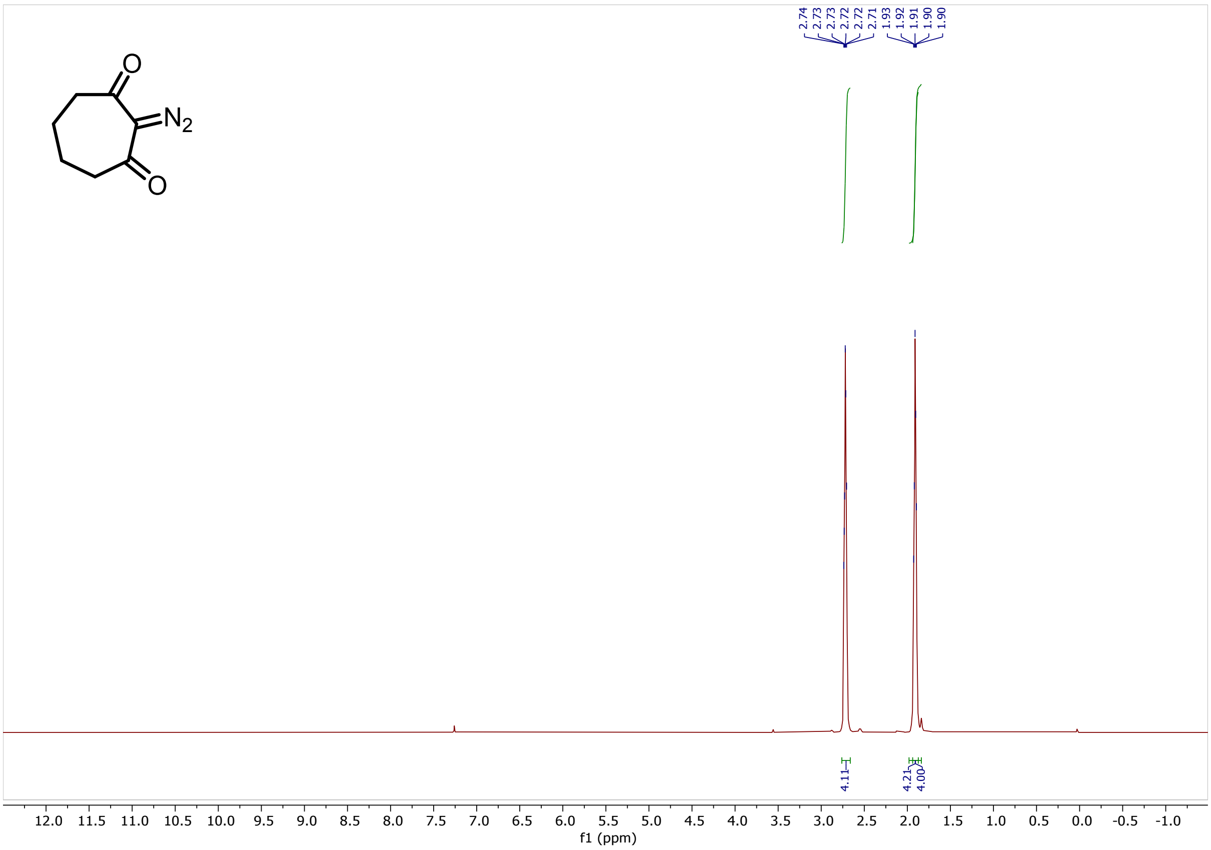

13C{1H} NMR (100 MHz, CDCl3)

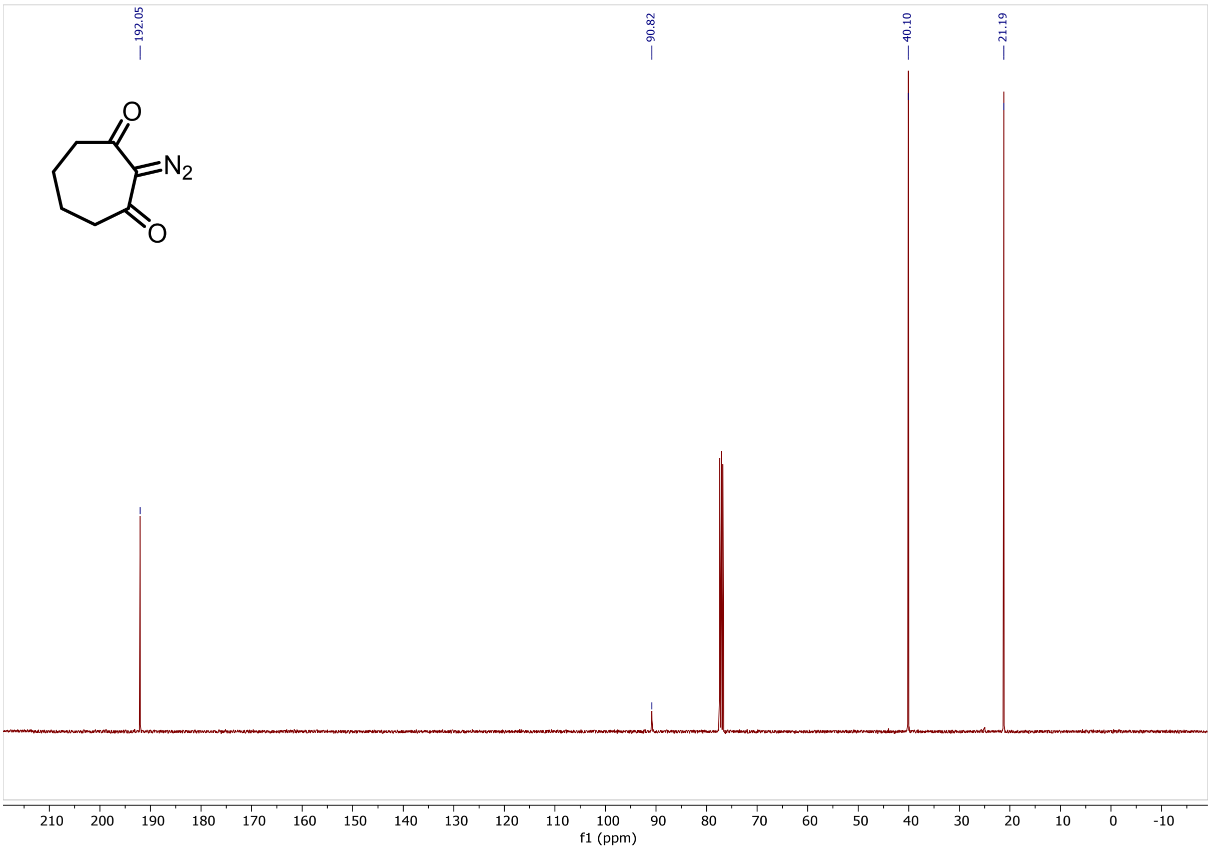

2-diazo-5-phenylcyclohexane-1,3-dione (1d)

^{1}H NMR (400 MHz, $CDCl_3$)

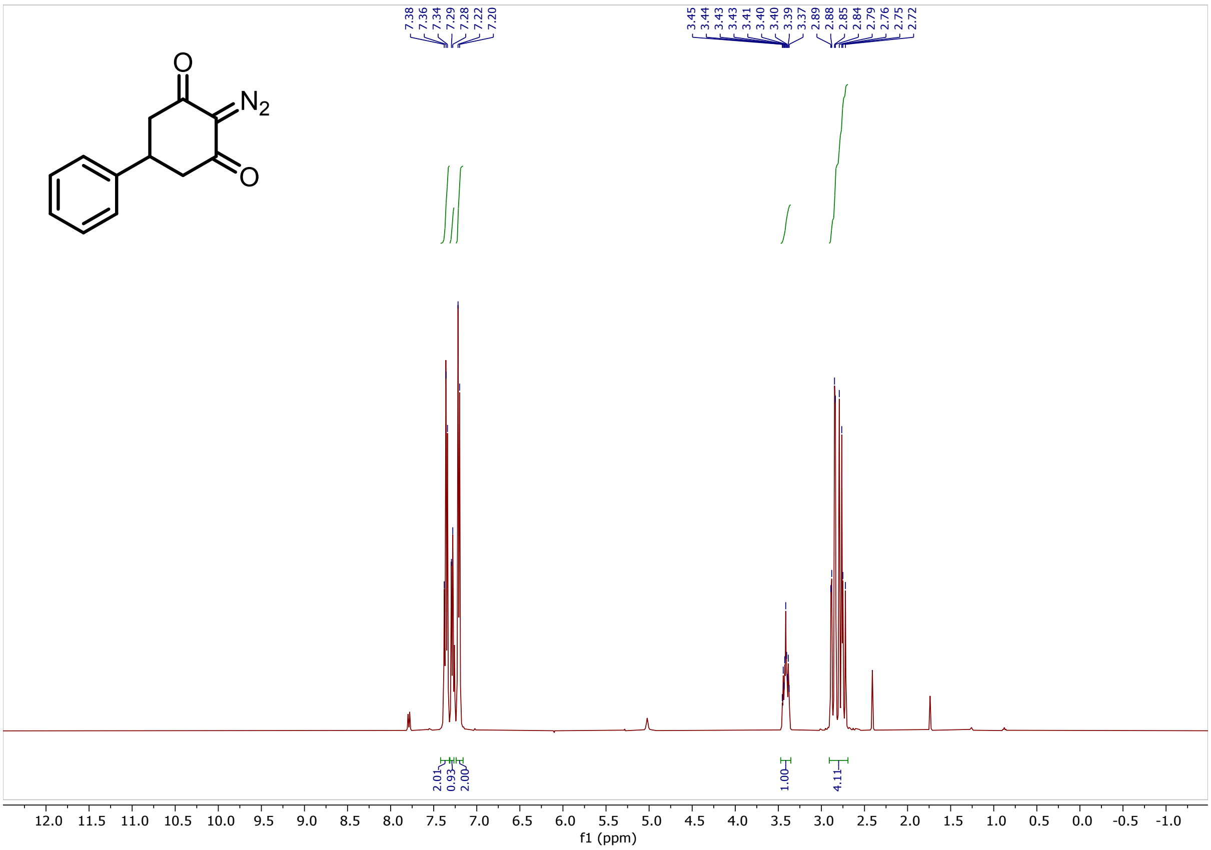

13C{1H} NMR (100 MHz, CDCl3)

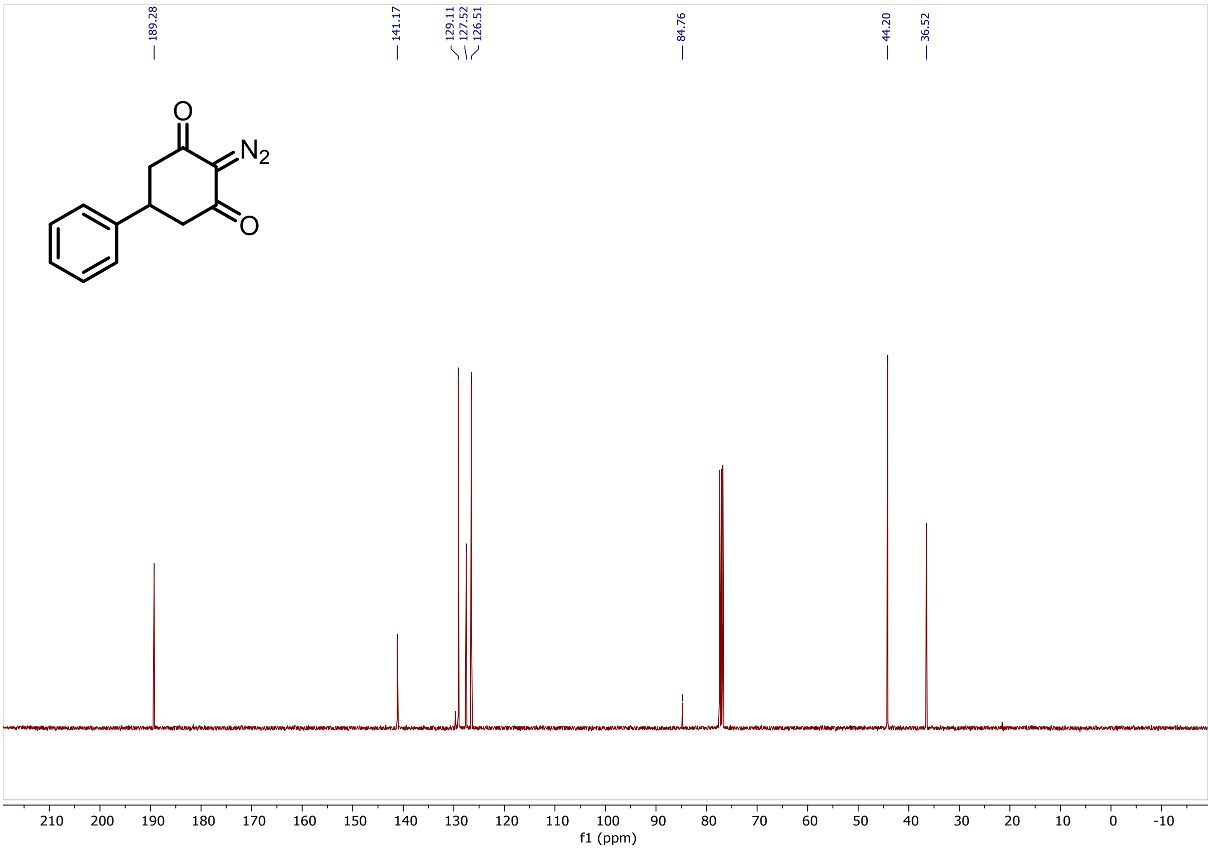

2-diazo-4,4-dimethylcyclohexane-1,3-dione (1e)

^1H NMR (400 MHz, $CDCl_3$)

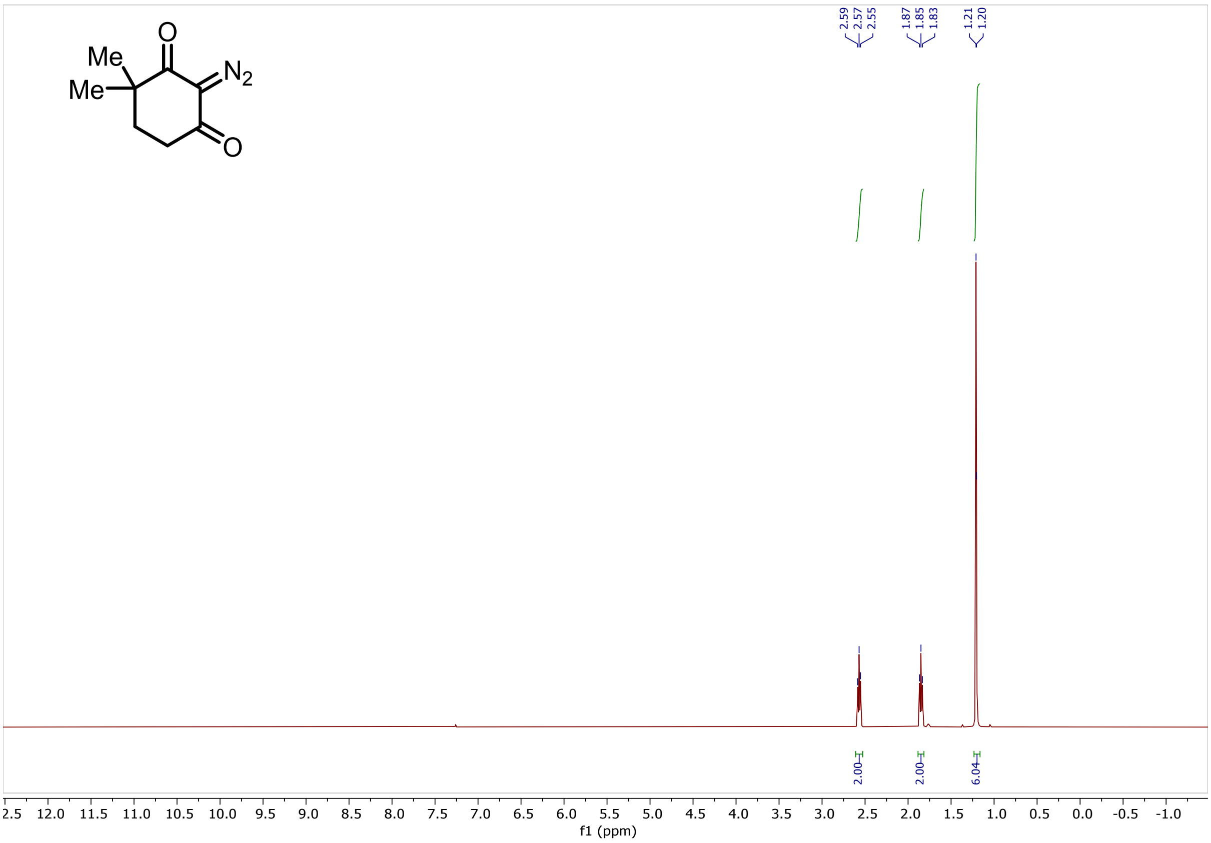

13C{1H} NMR (100 MHz, CDCl3)

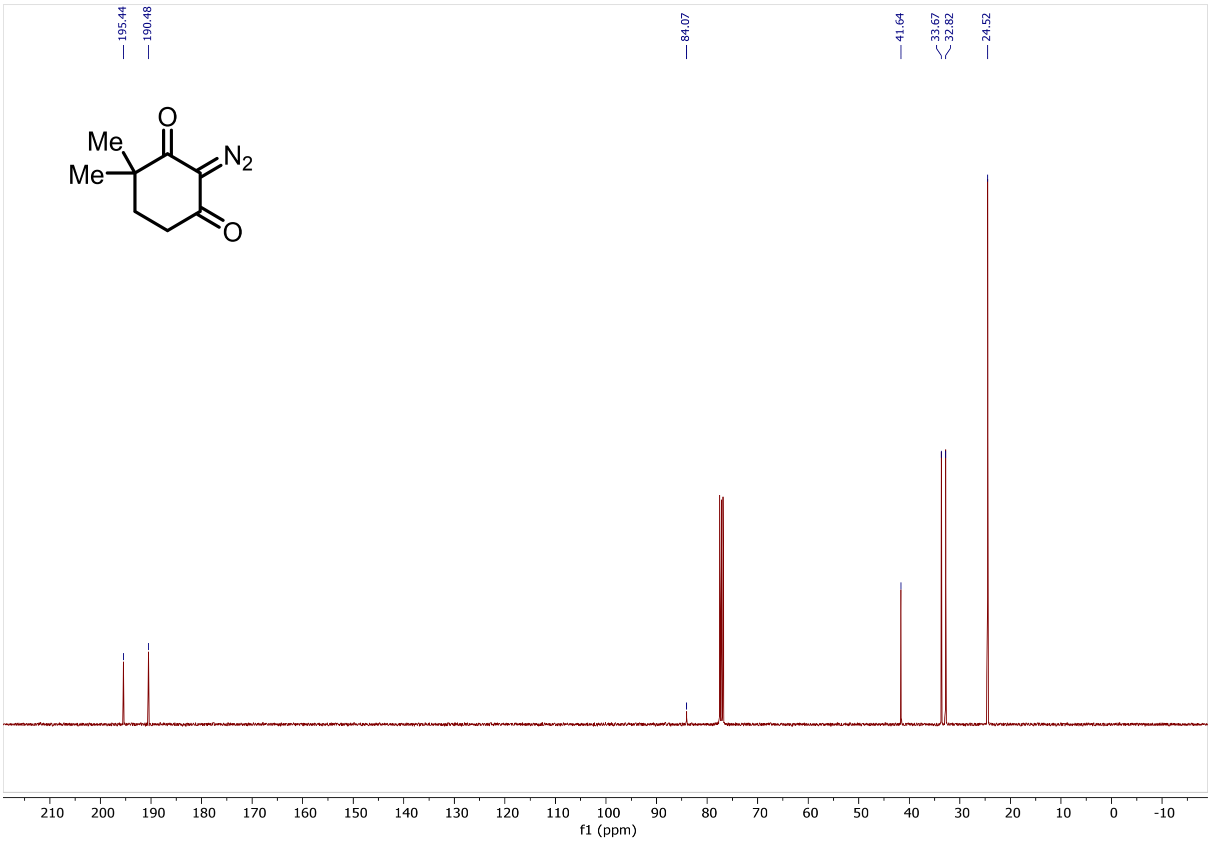

3-diazopentane-2,4-dione (1f)

^1H NMR (500 MHz, $CDCl_3$)

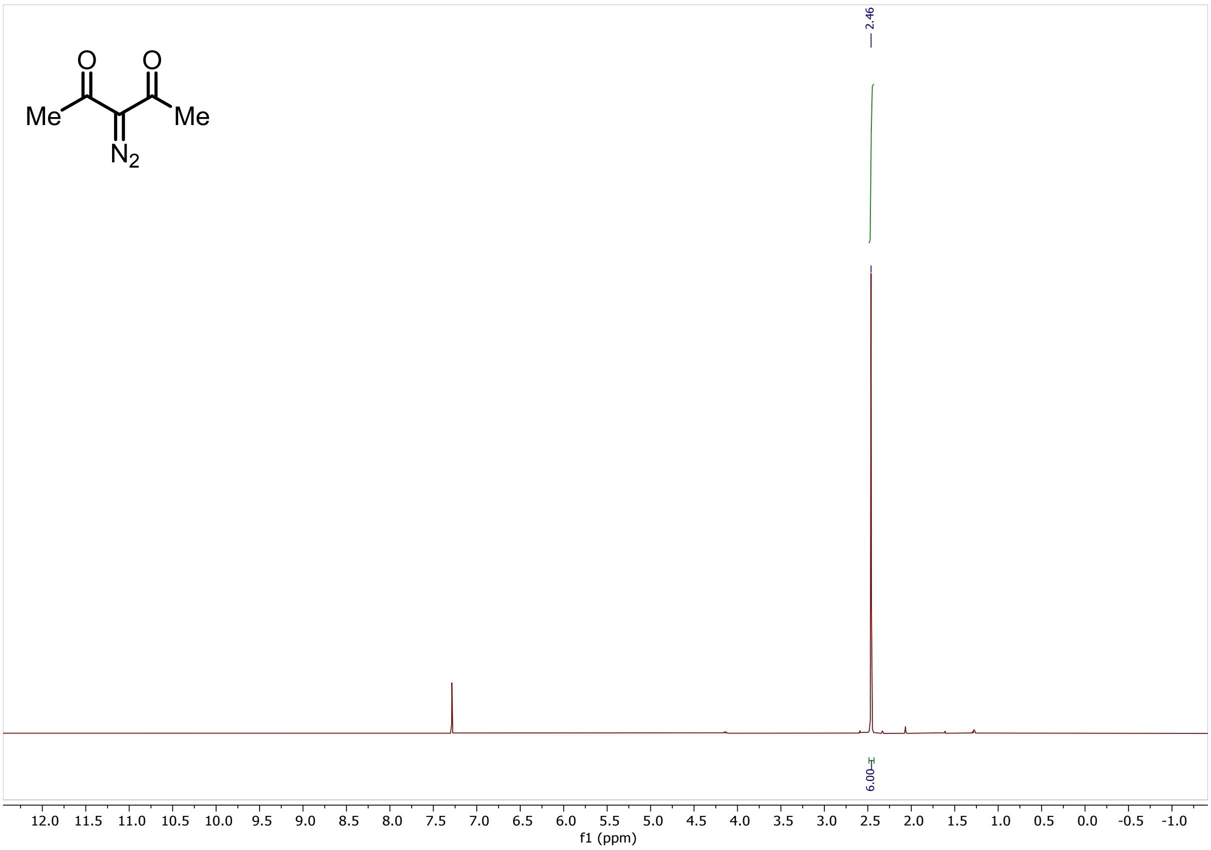

13C{1H} NMR (126 MHz, CDCl3)

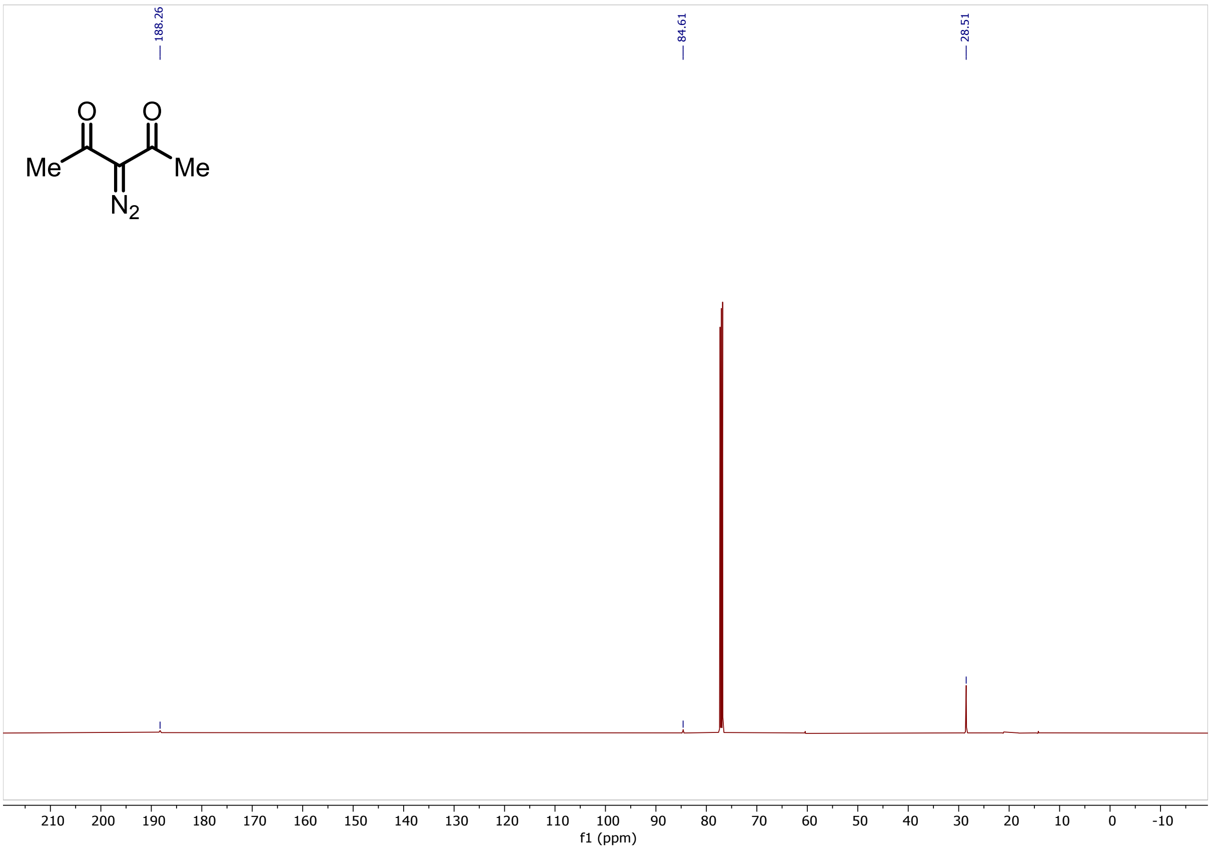

2-diazocyclopentane-1,3-dione (1g)

^{1}H NMR (400 MHz, $CDCl_3$)

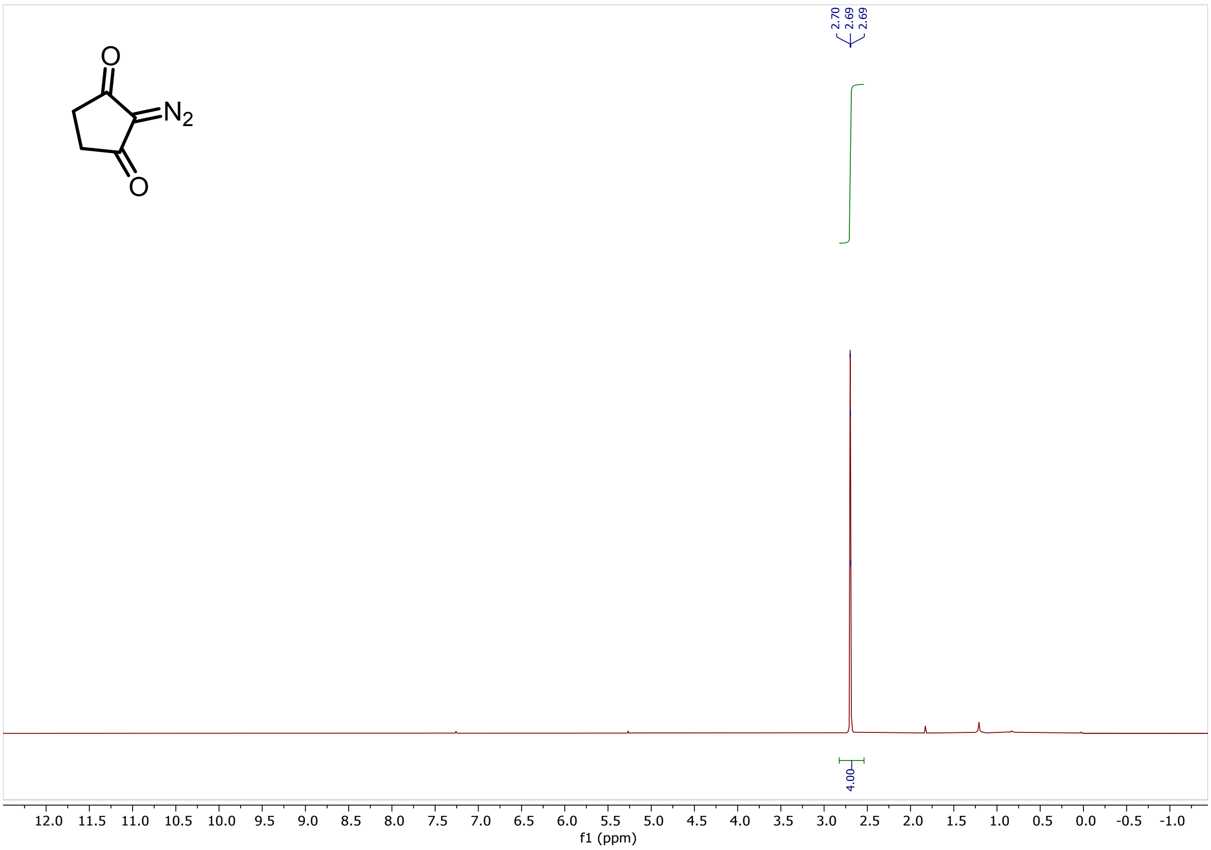

13C{1H} NMR (100 MHz, CDCl3)

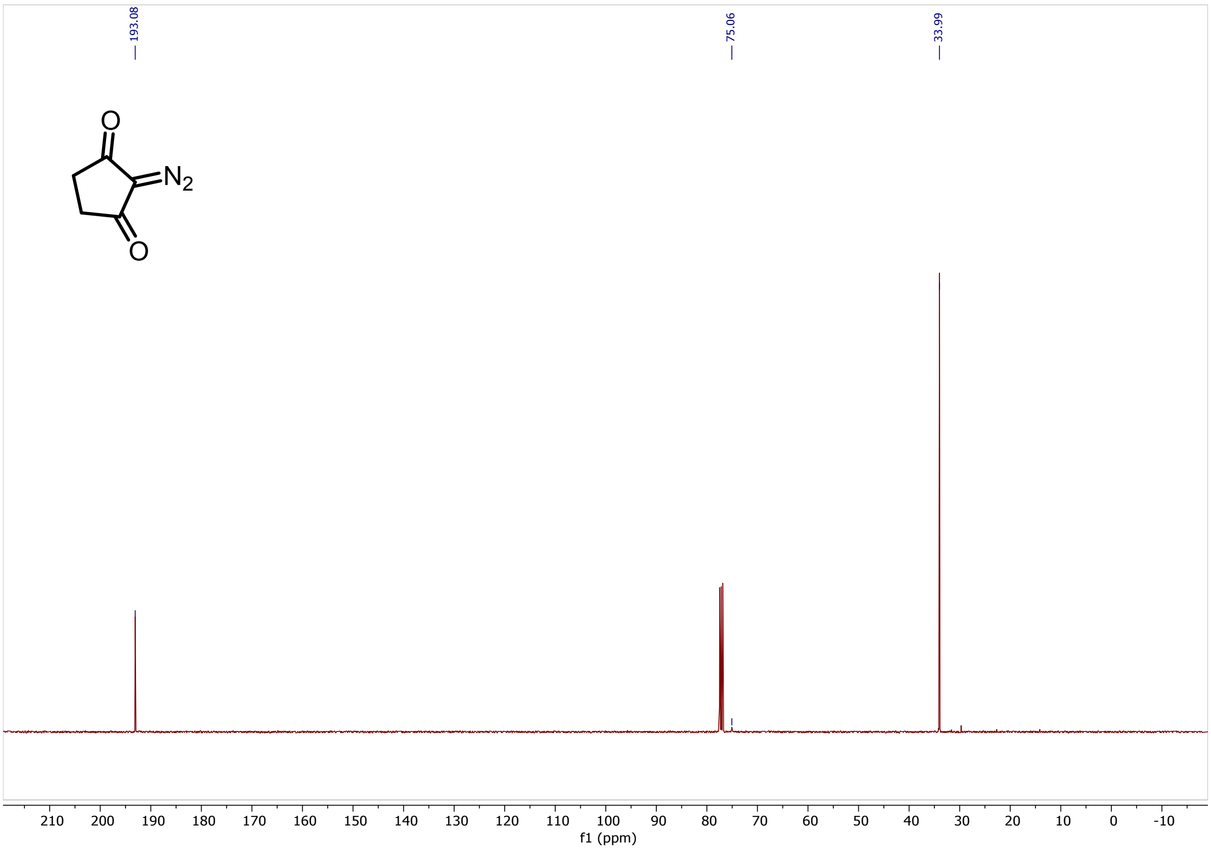

Butyl 4,4-dimethyl-2-oxocyclopentane-1-carboxylate (2a)

^{1}H NMR (500 MHz, $CDCl_3$)

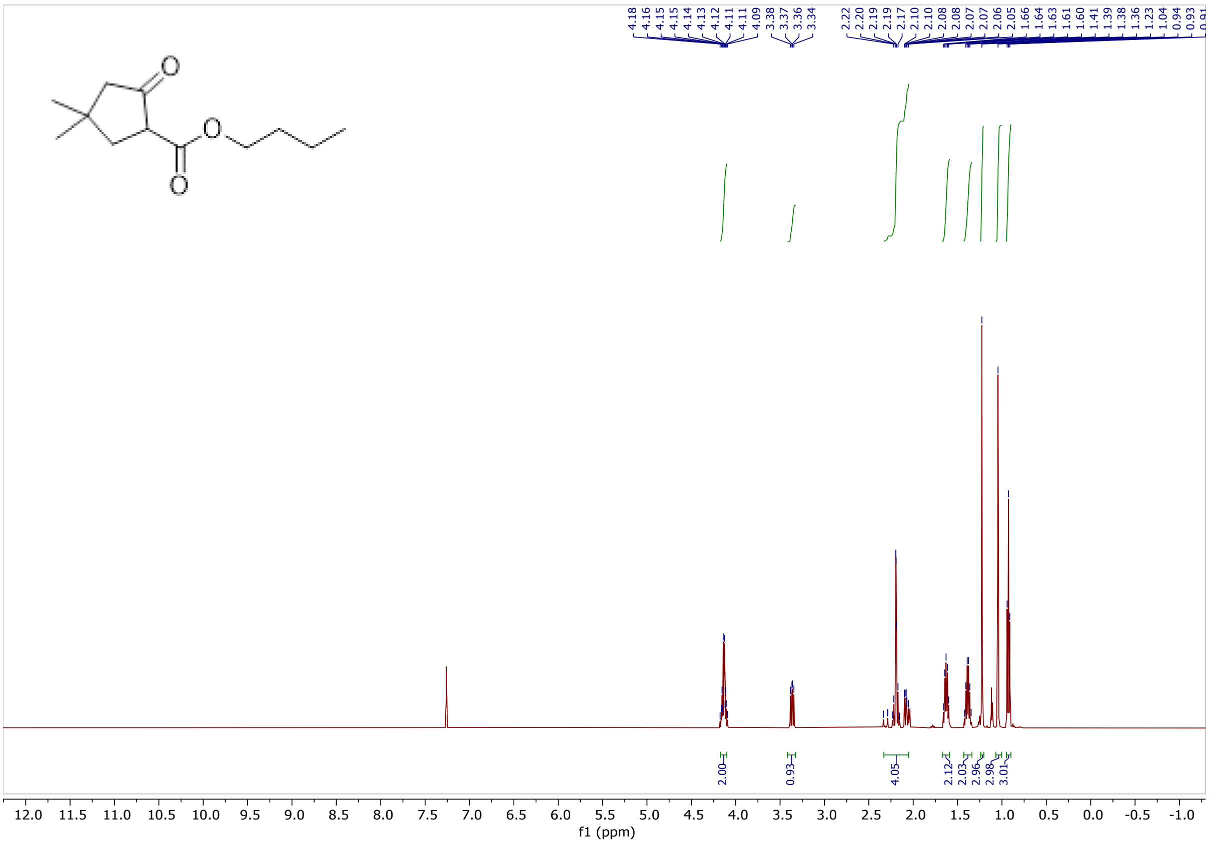

13C{1H} NMR (126 MHz, CDCl3)

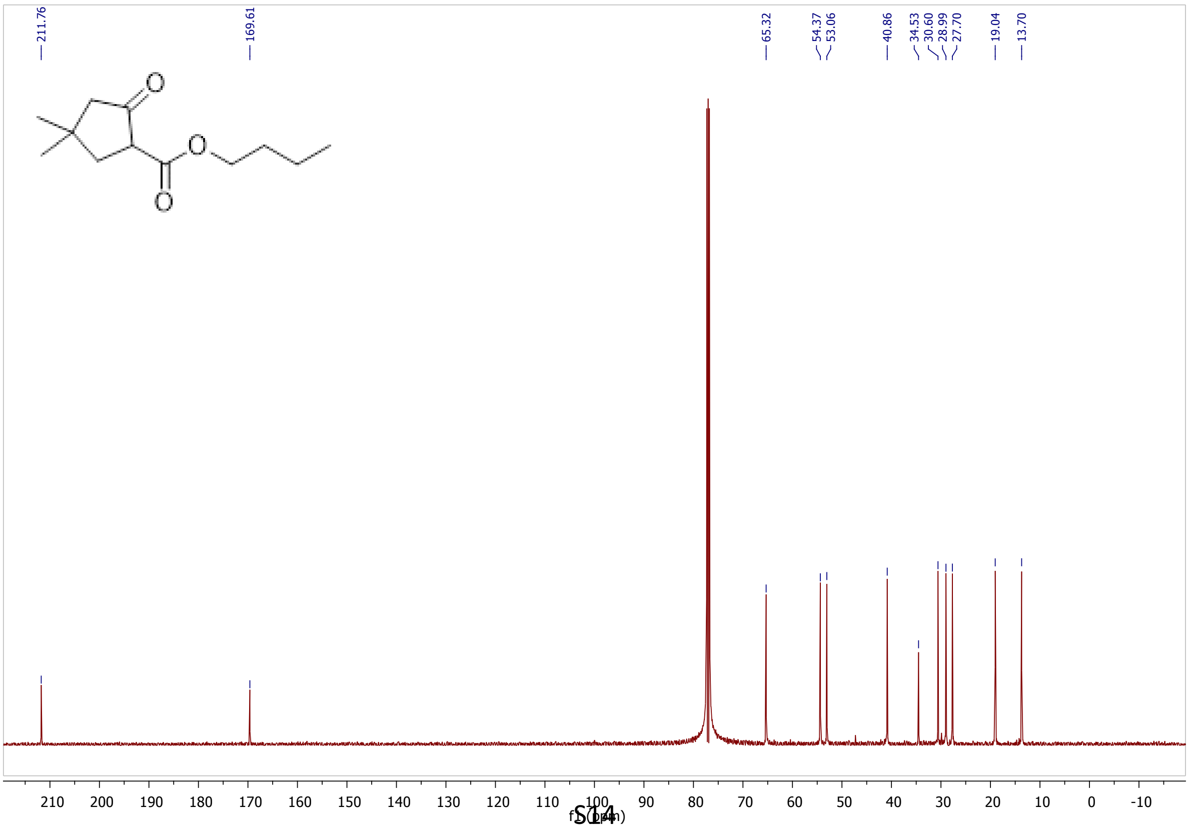

butyl 2-oxocyclopentanecarboxylate (2b)

^{1}H NMR (400 MHz, $CDCl_3$)

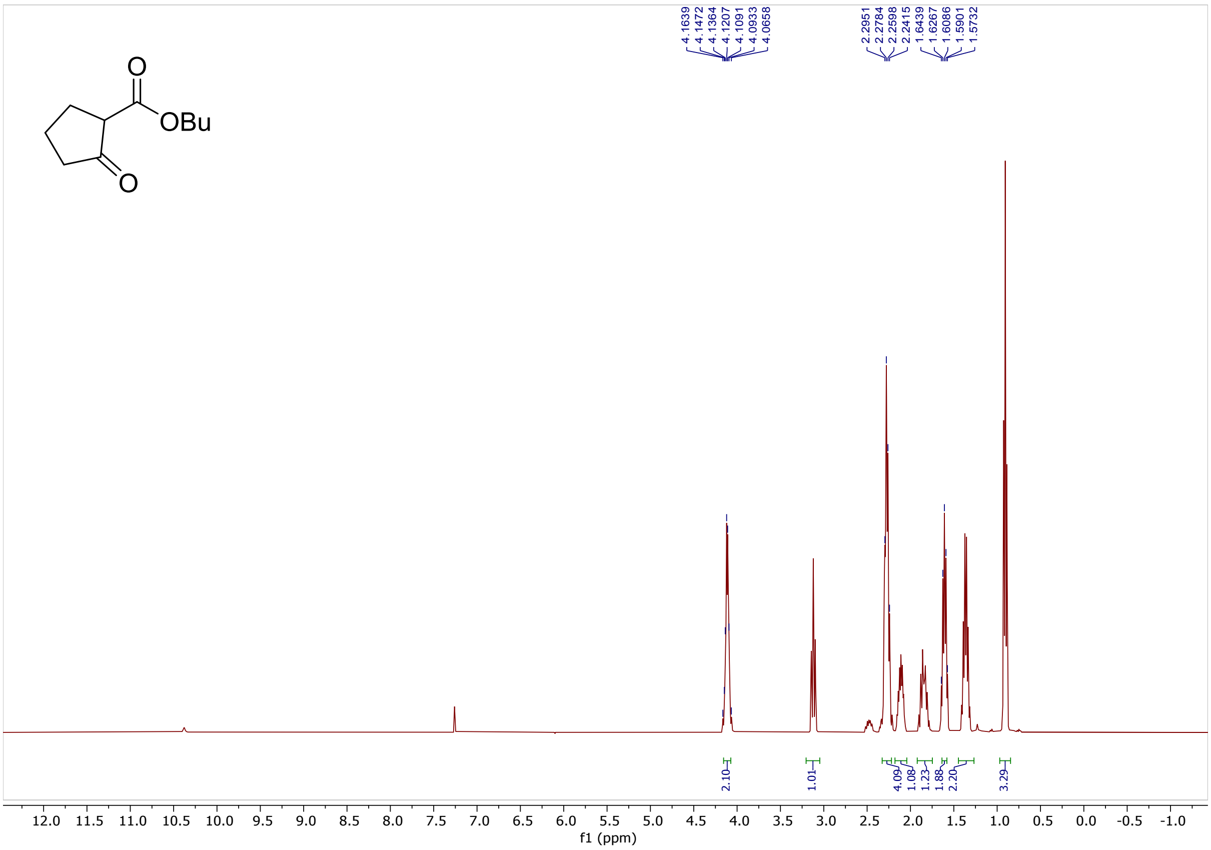

13C{1H} NMR (100 MHz, CDCl3)

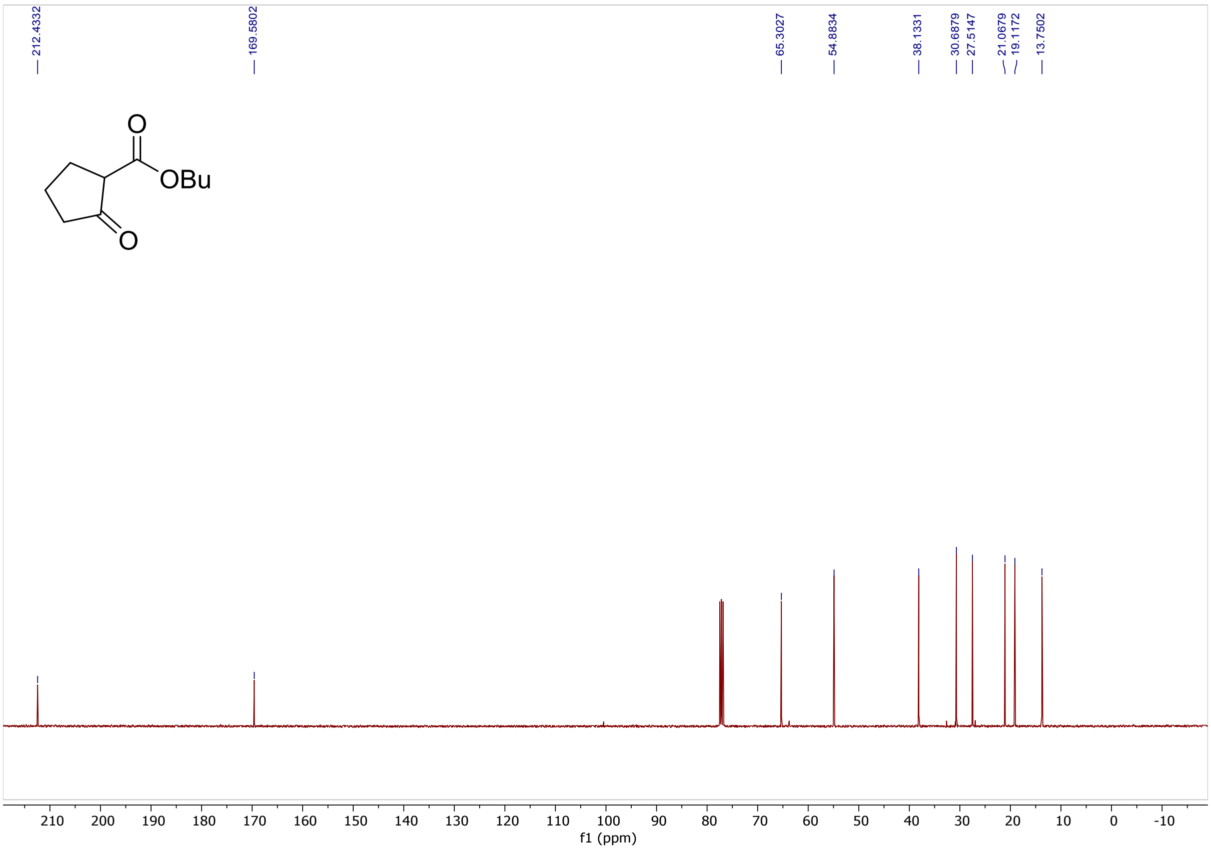

butyl 2-hydroxycyclohex-1-enecarboxylate (2c)

^{1}H NMR (400 MHz, $CDCl_3$)

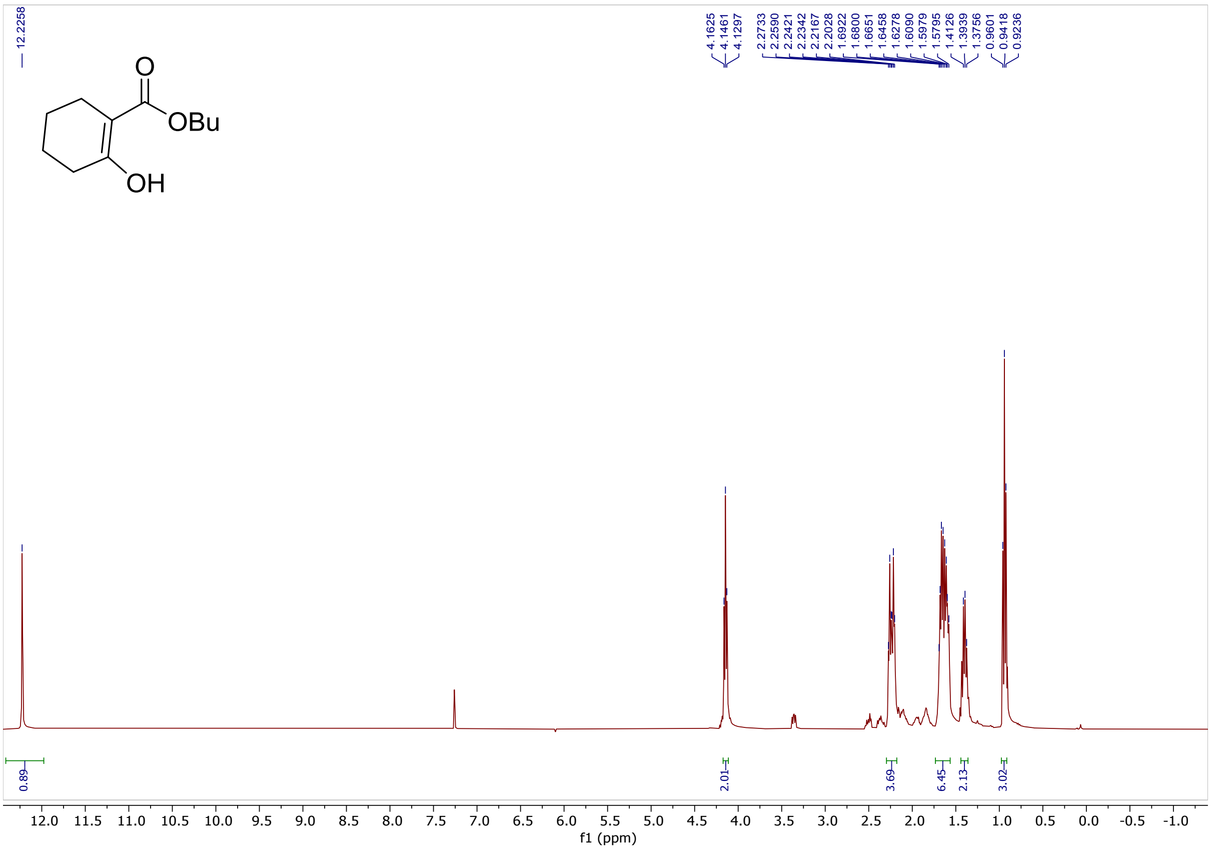

13C{1H} NMR (100 MHz, CDCl3)

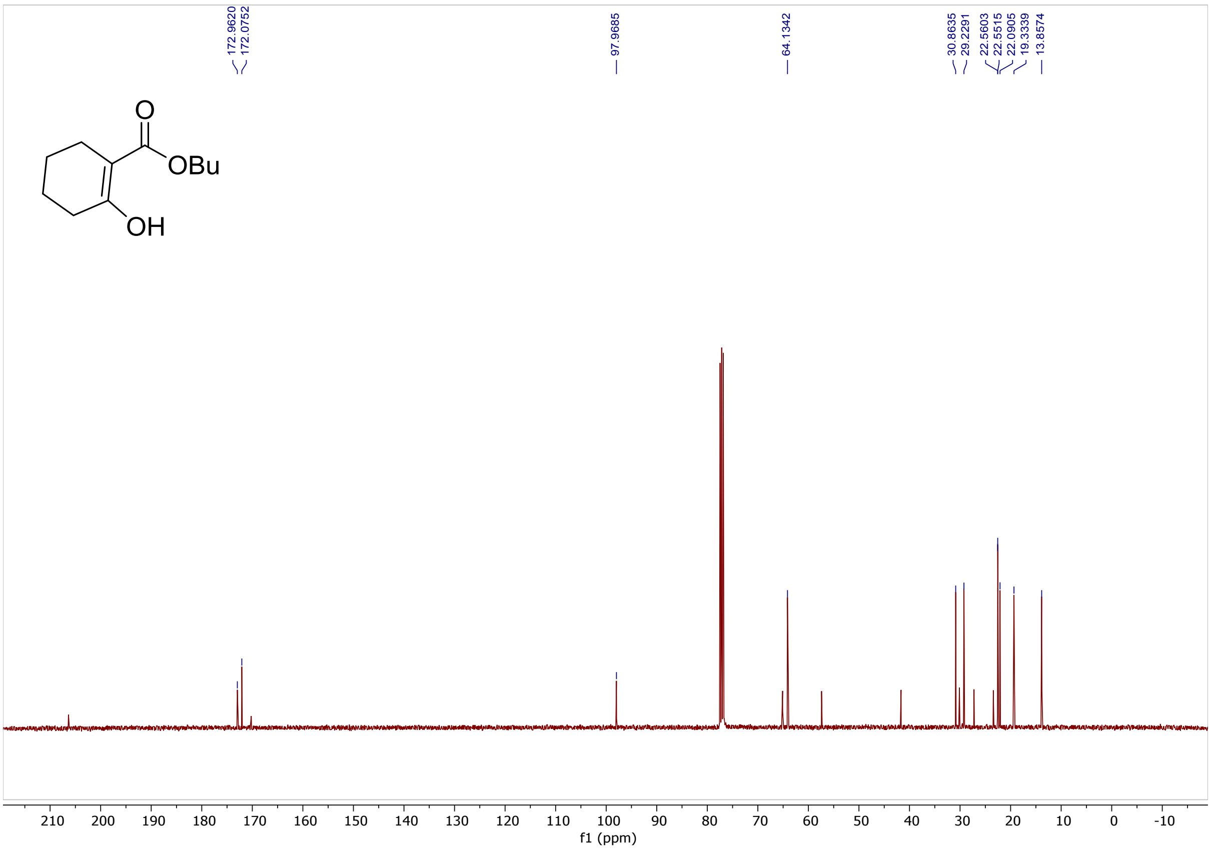

butyl 2-oxo-4-phenylcyclopentanecarboxylate (2d)

^{1}H NMR (400 MHz, $CDCl_3$)

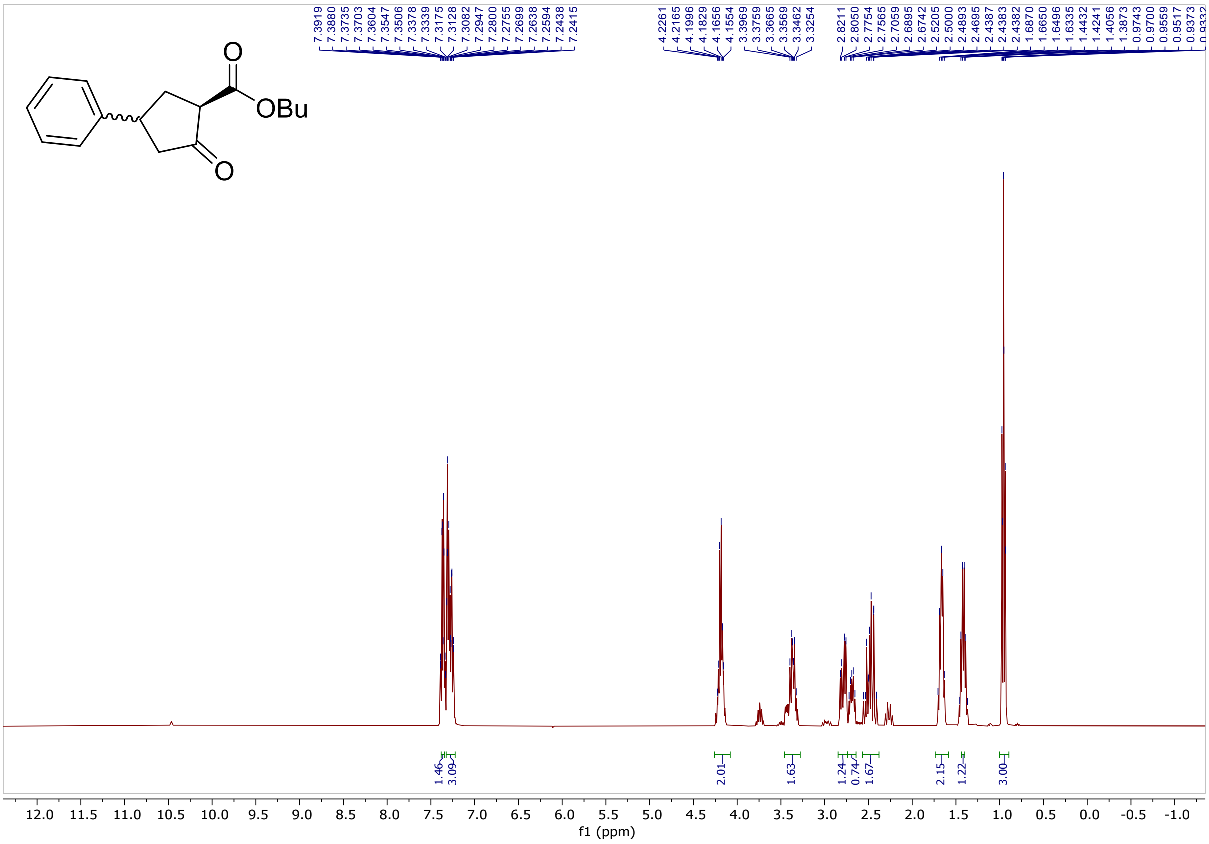

13C{1H} NMR (100 MHz, CDCl3)

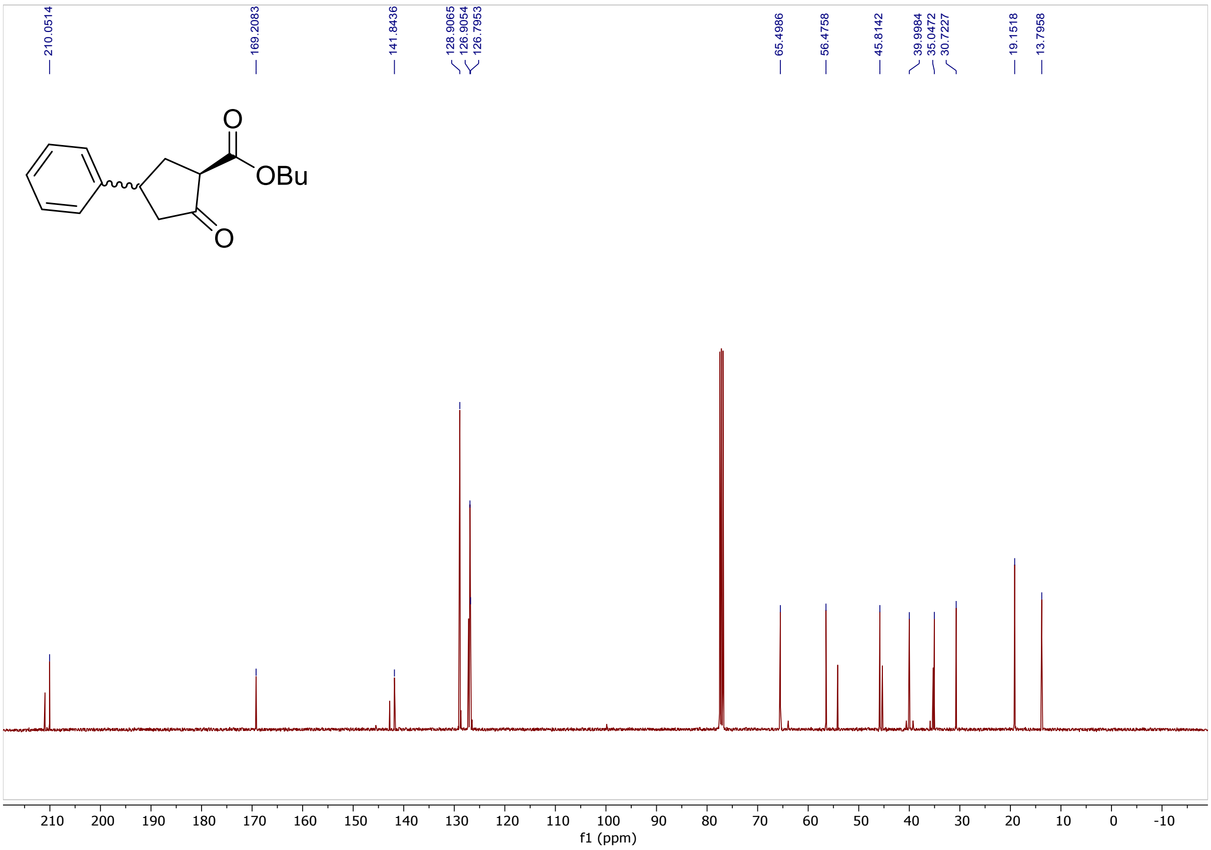

butyl 2,2-dimethyl-5-oxocyclopentanecarboxylate (2ea) and butyl 3,3-dimethyl-2-oxocyclopentanecarboxylate (2eb).

^{1}H NMR (400 MHz, $CDCl_3$)

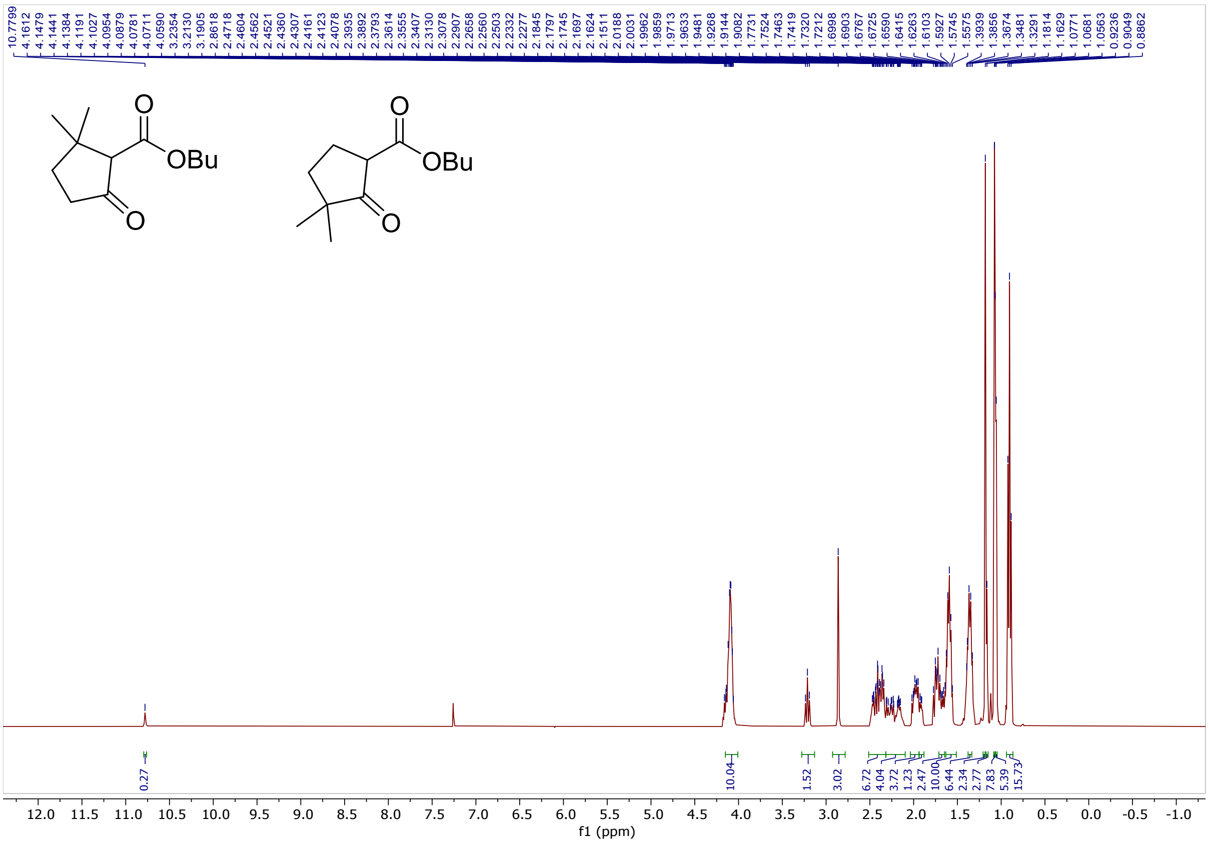

13C{1H} NMR (100 MHz, CDCl3)

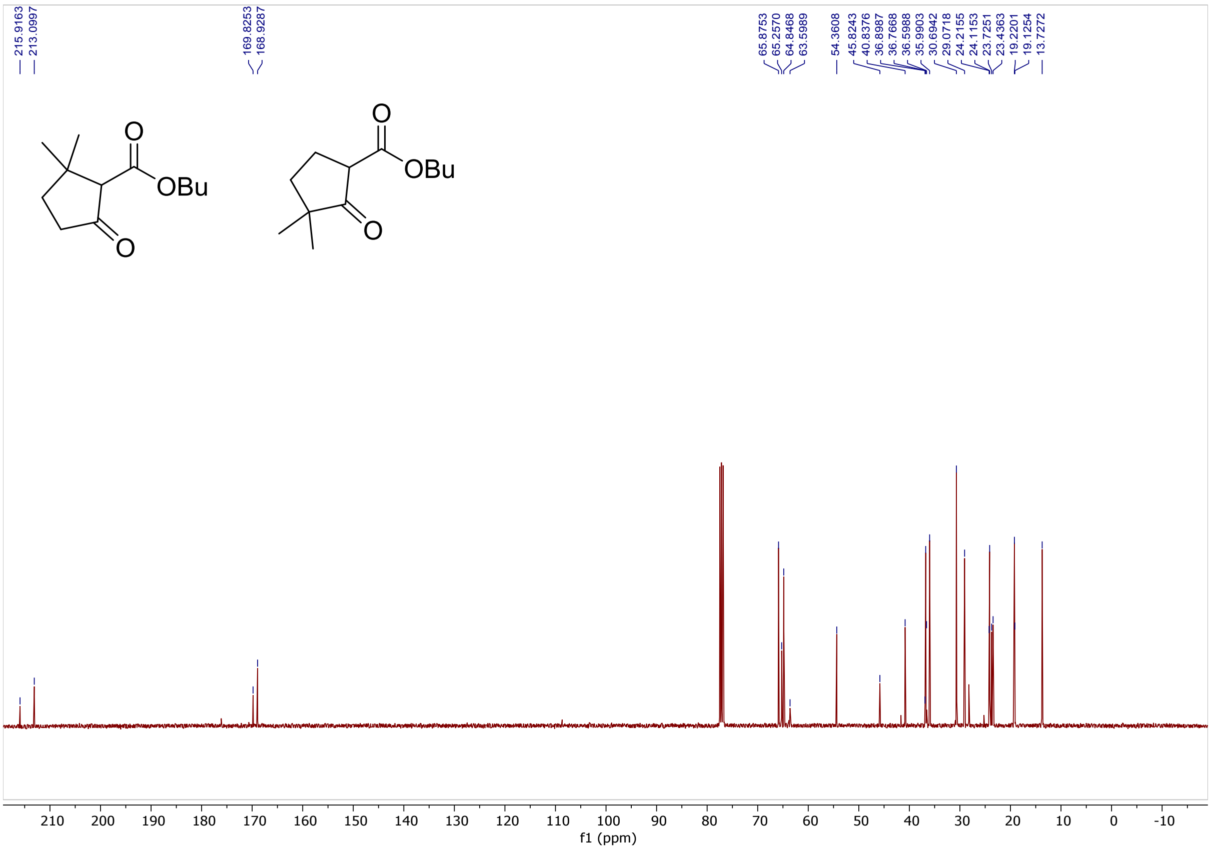

butyl 2-methyl-3-oxobutanoate (2f)

^{1}H NMR (400 MHz, $CDCl_3$)

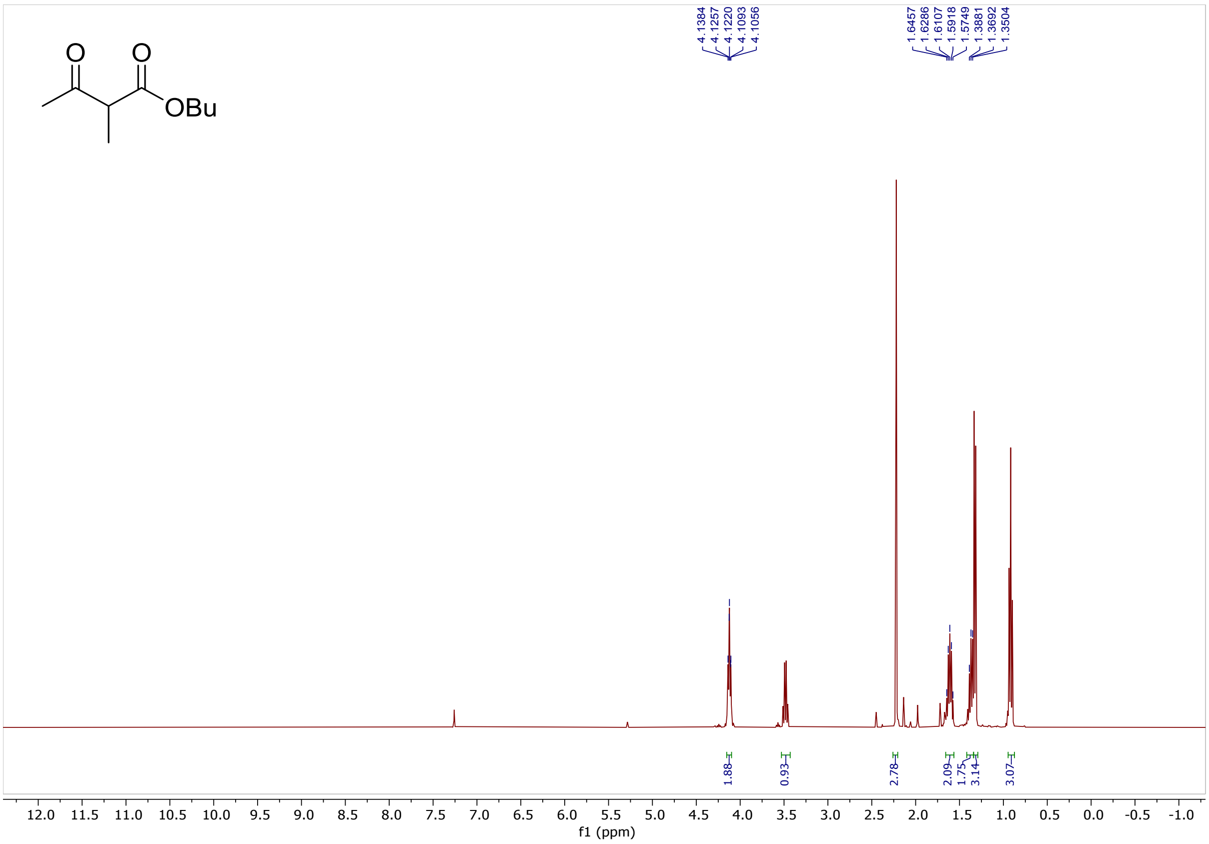

13C{1H} NMR (100 MHz, CDCl3)

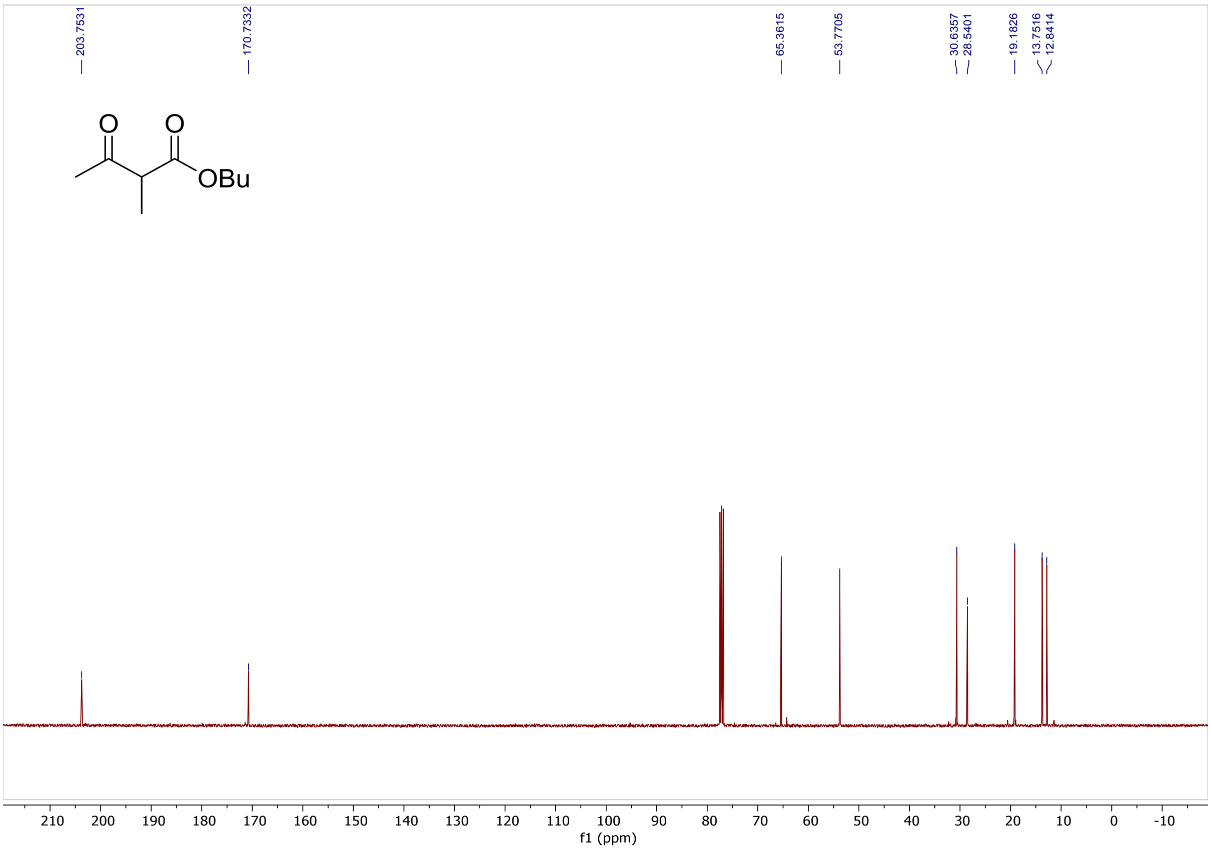

Benzyl 4,4-dimethyl-2-oxocyclopentane-1-carboxylate (2ab)

^{1}H NMR (500 MHz, $CDCl_3$)

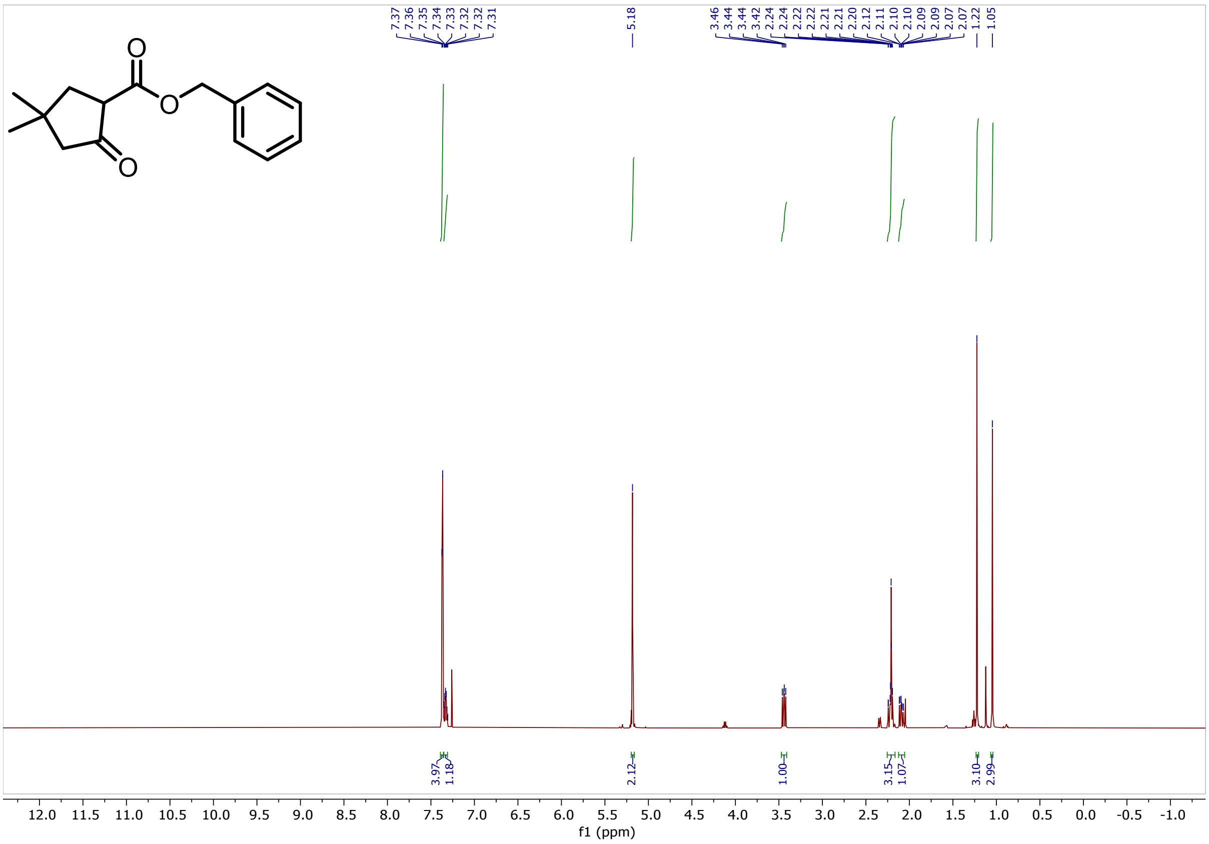

13C{1H} NMR (126 MHz, CDCl3)

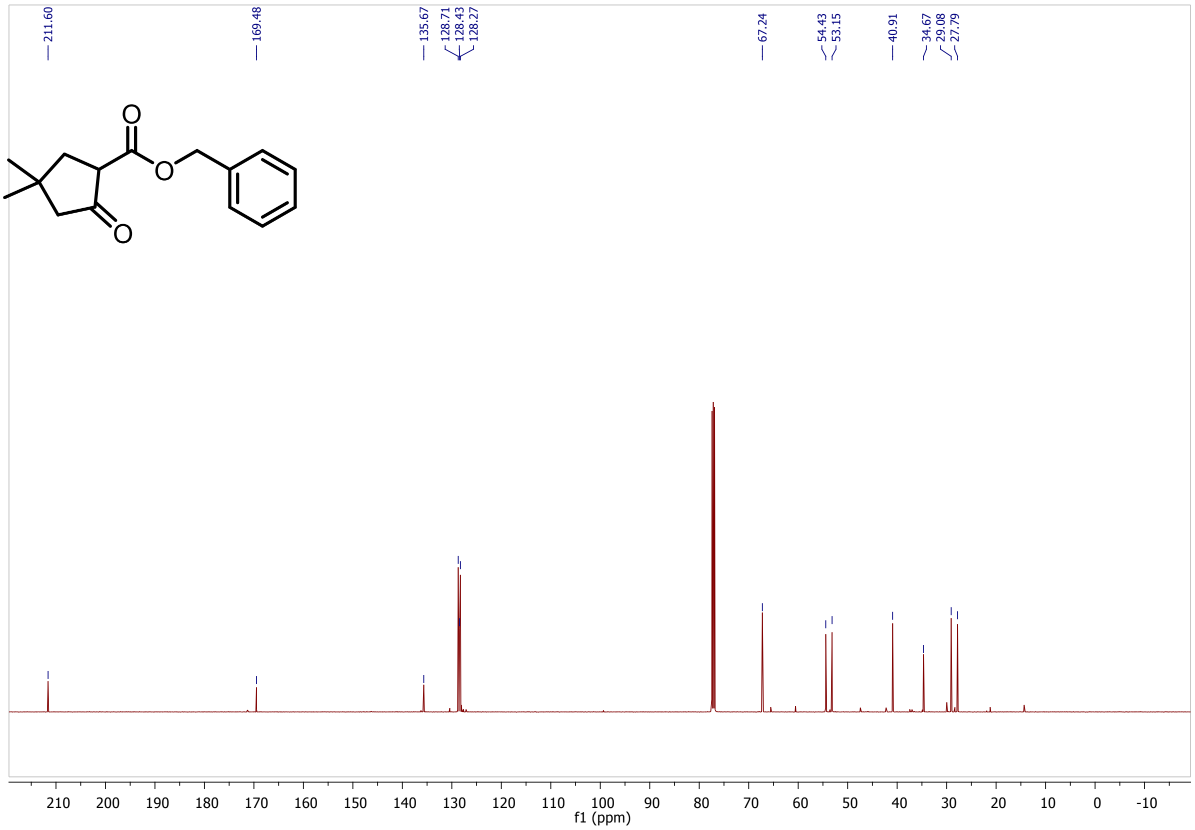

Cyclopentyl 4,4-dimethyl-2-oxocyclopentane-1-carboxylate (2ac)

^{1}H NMR (500 MHz, $CDCl_3$)

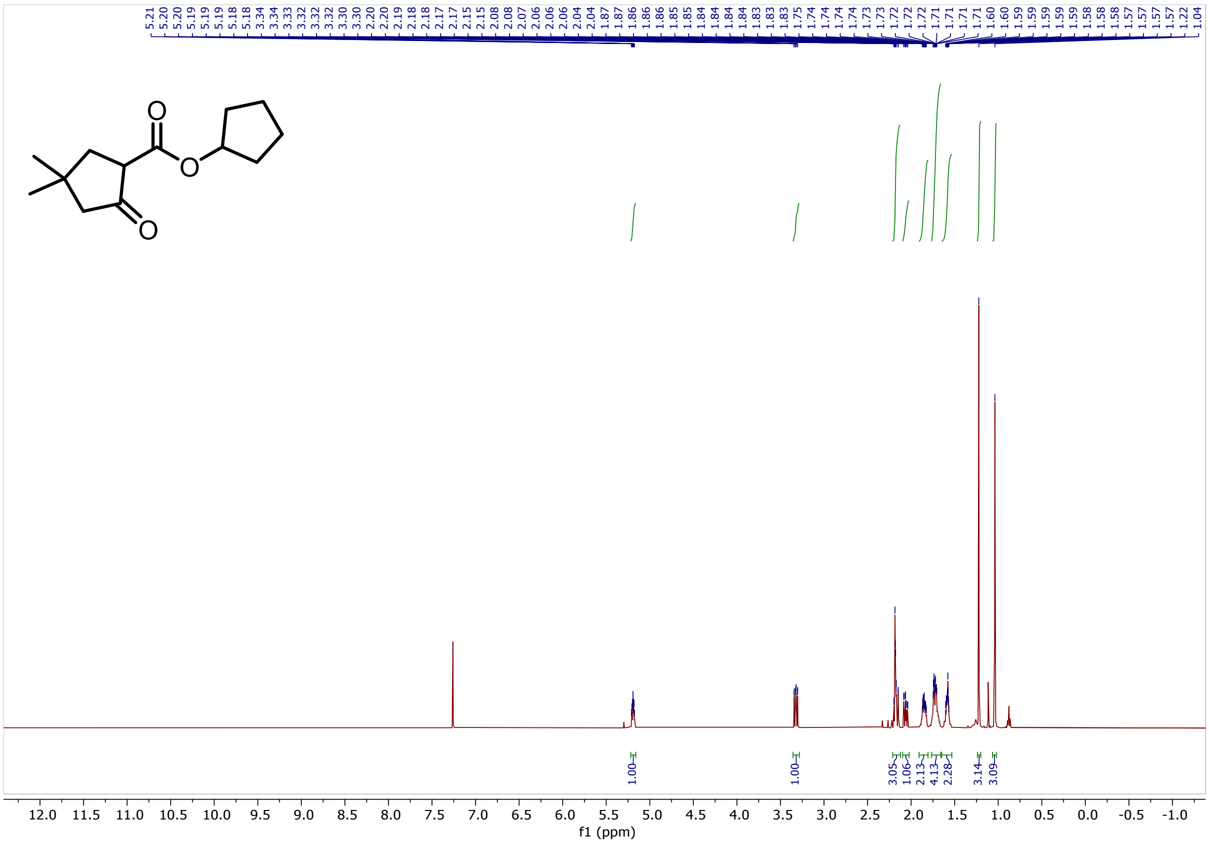

13C{1H} NMR (126 MHz, CDCl3)

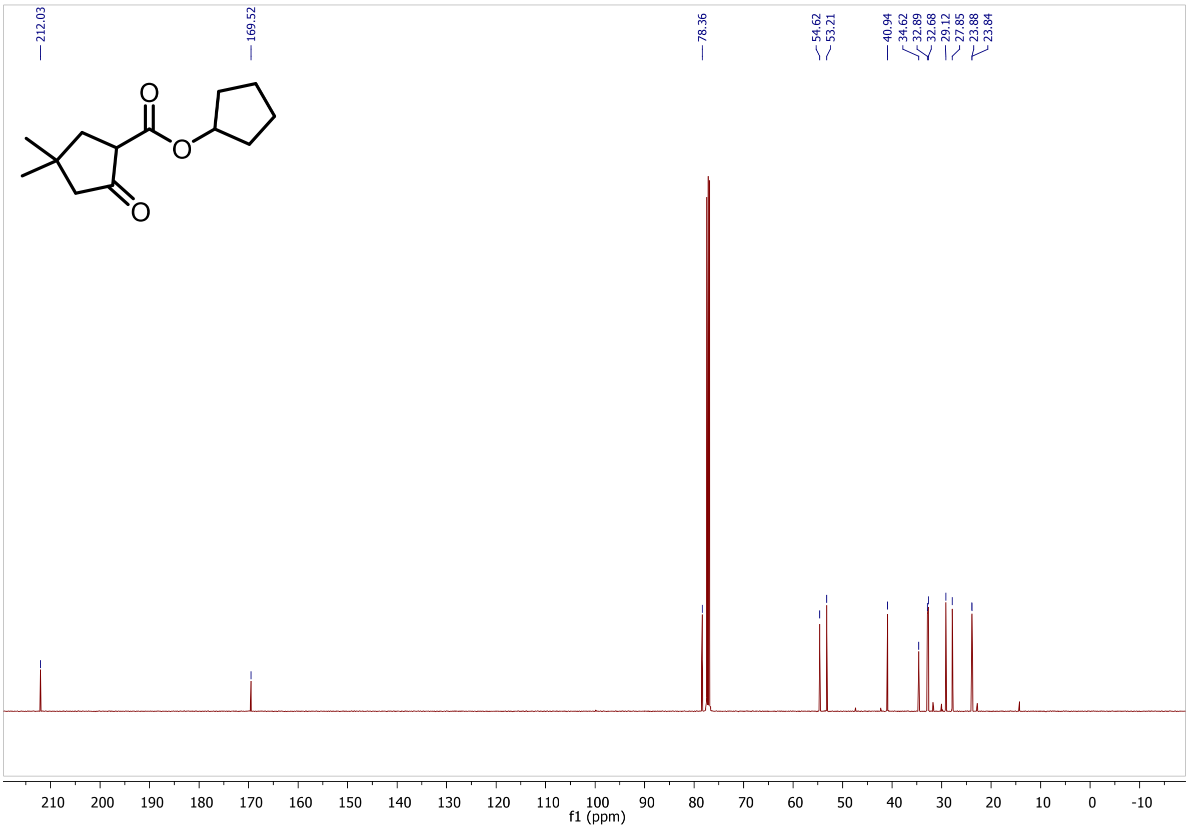

S-phenyl 4,4-dimethyl-2-oxocyclopentane-1-carbothioate (3a) and 5,5-dimethyl-2-(phenylthio)cyclohexane-1,3-dione (3b)

^{1}H NMR (500 MHz, $CDCl_3$)

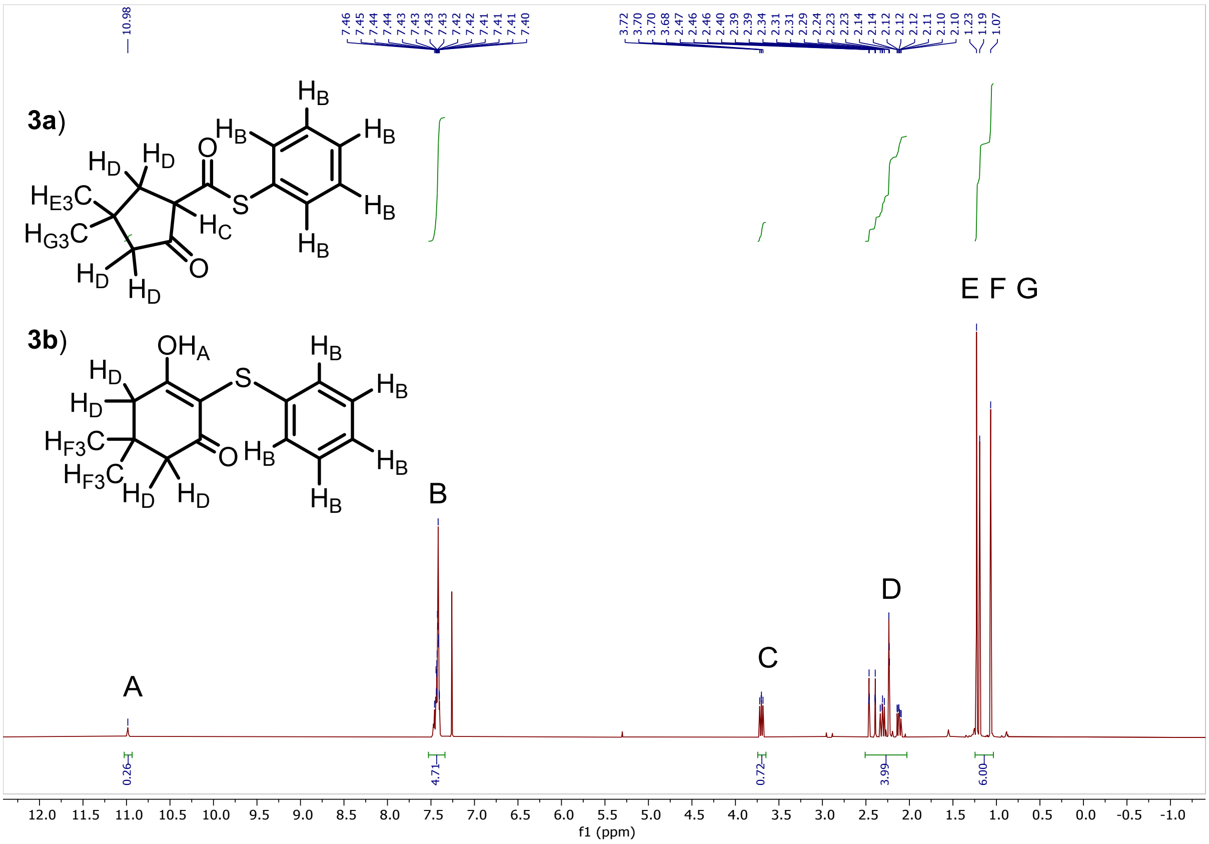

13C{1H} NMR (126 MHz, CDCl3)

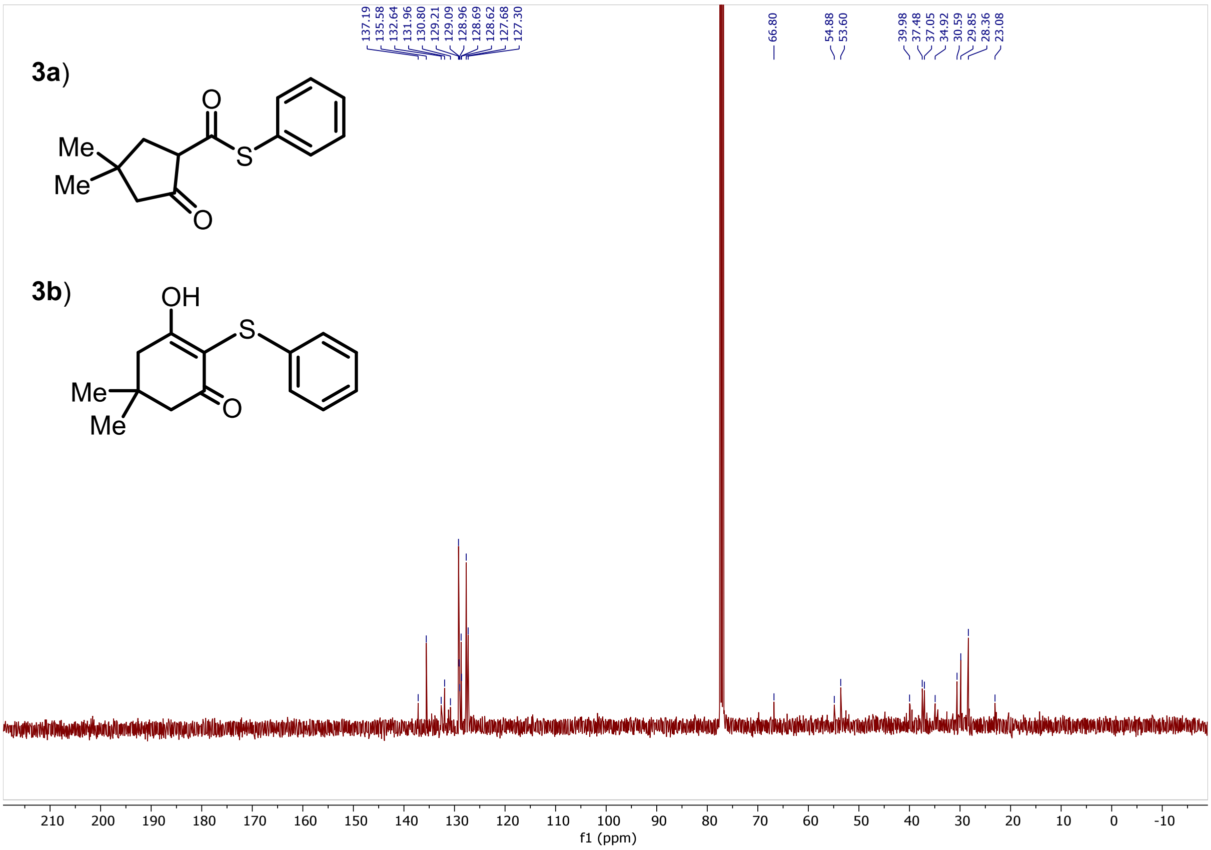

S-benzyl 4,4-dimethyl-2-oxocyclopentane-1-carbothioate (3c) and 2-(benzylthio)-5,5-dimethylcyclohexane-1,3-dione (3d).

^{1}H NMR (500 MHz, $CDCl_3$)

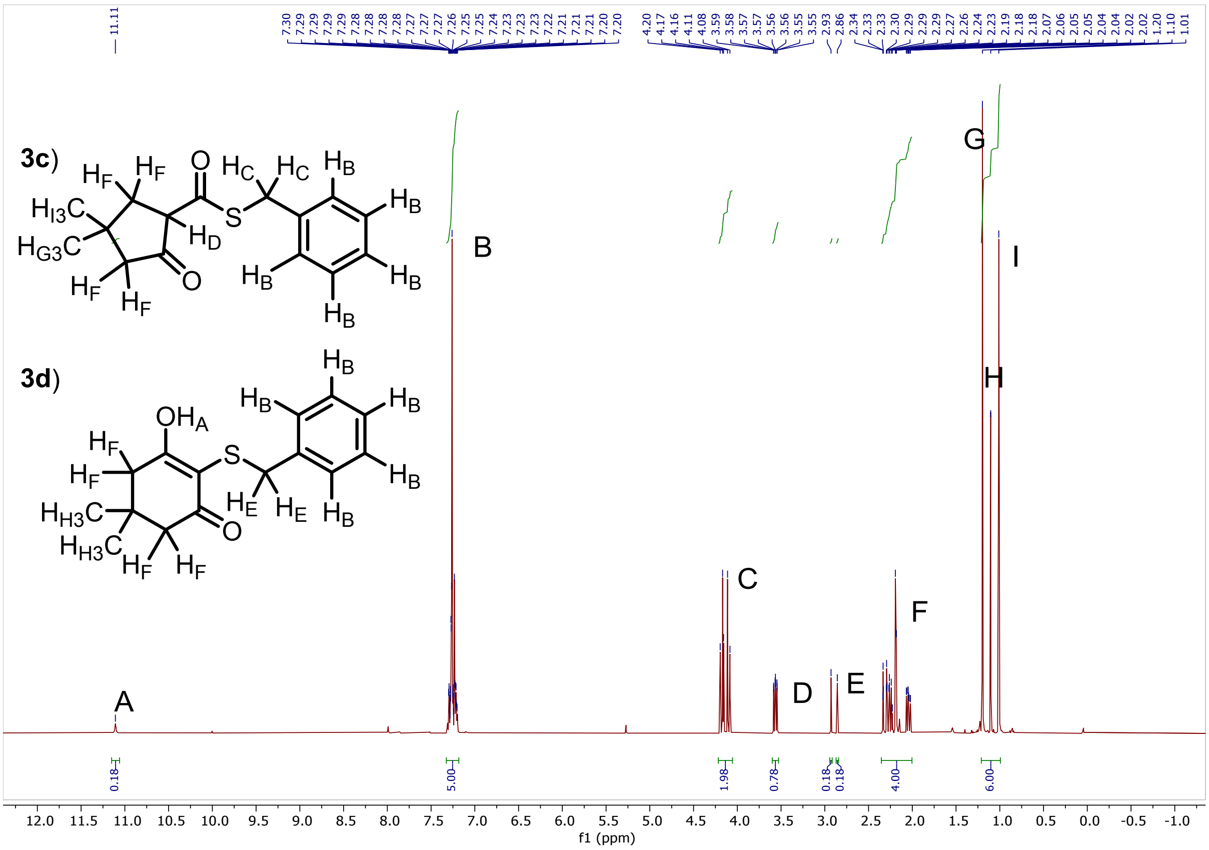

13C{1H} NMR (126 MHz, CDCl3)

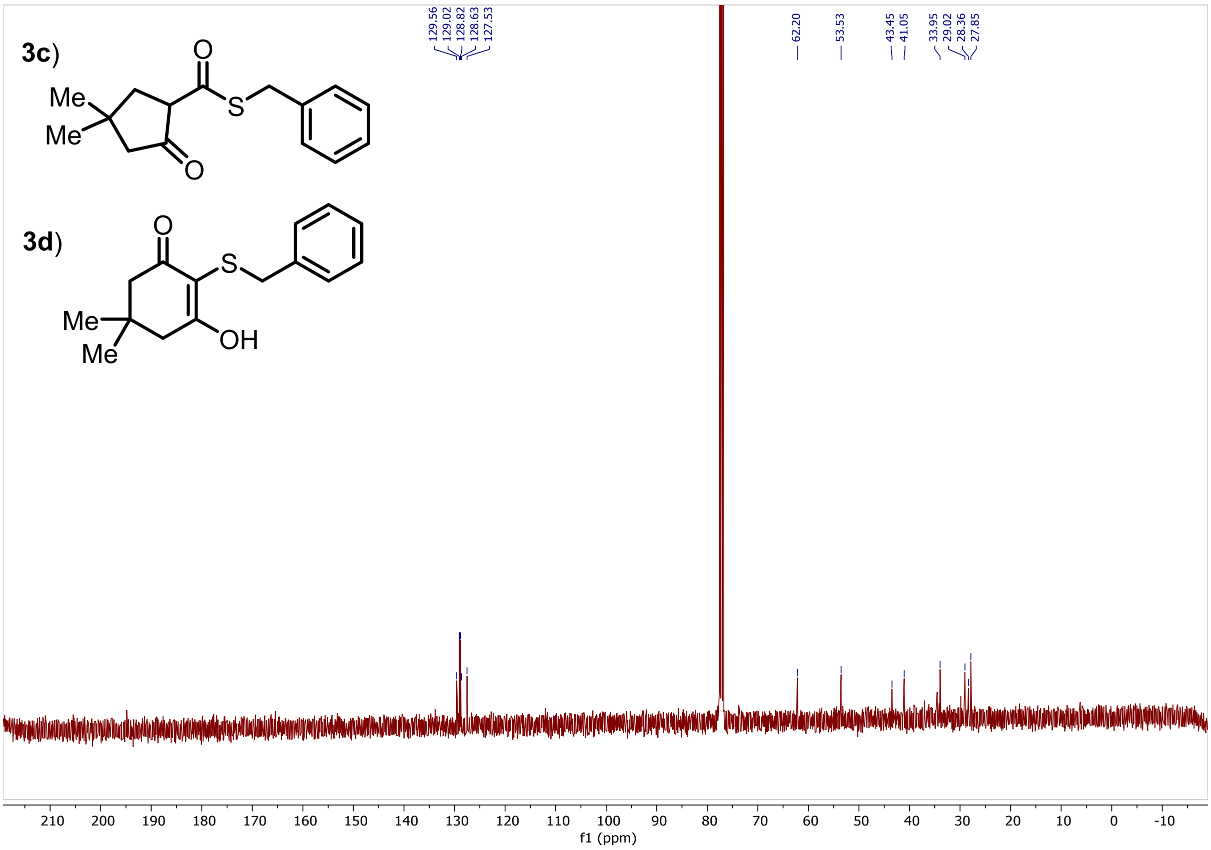

N-butyl 4,4-dimethyl-2-oxocyclopentane-1-carboxamide (4a)

^{1}H NMR (500 MHz, $CDCl_3$)

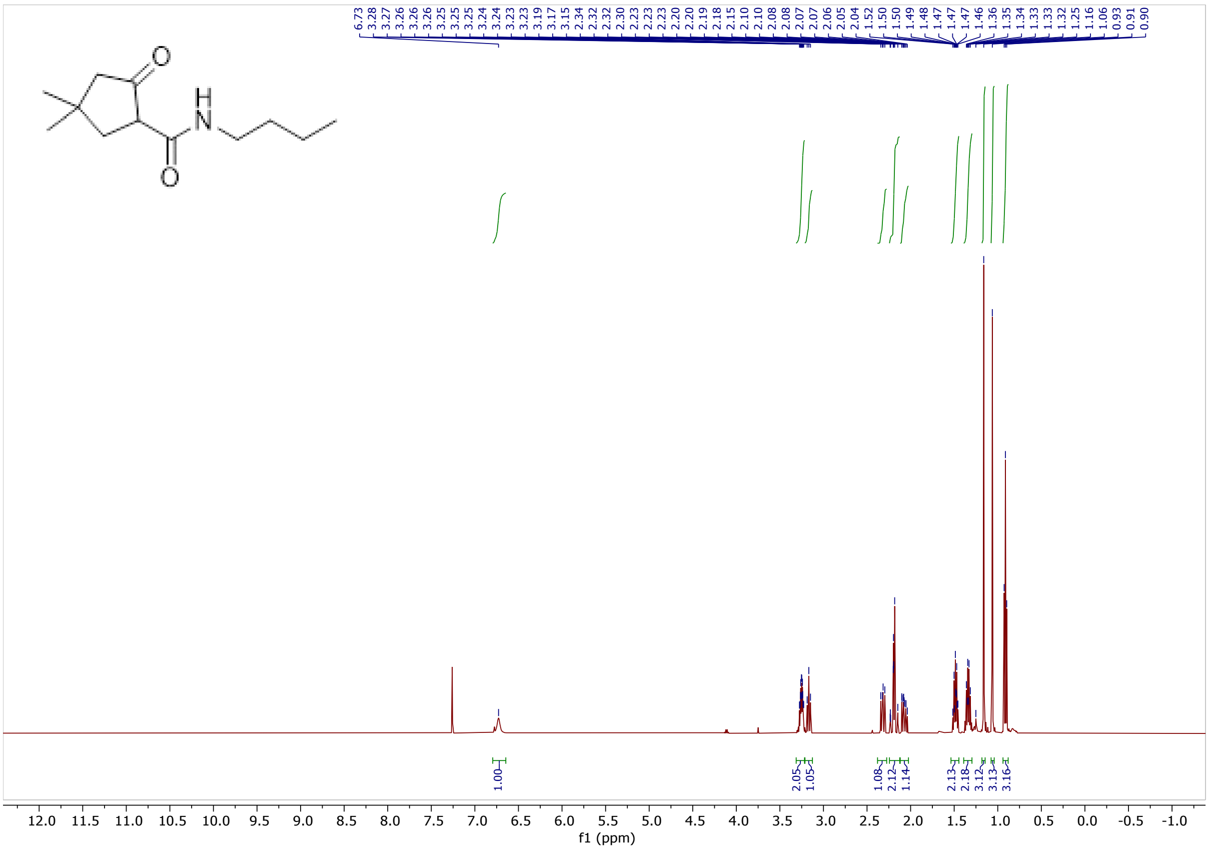

13C{1H} NMR (126 MHz, CDCl3)

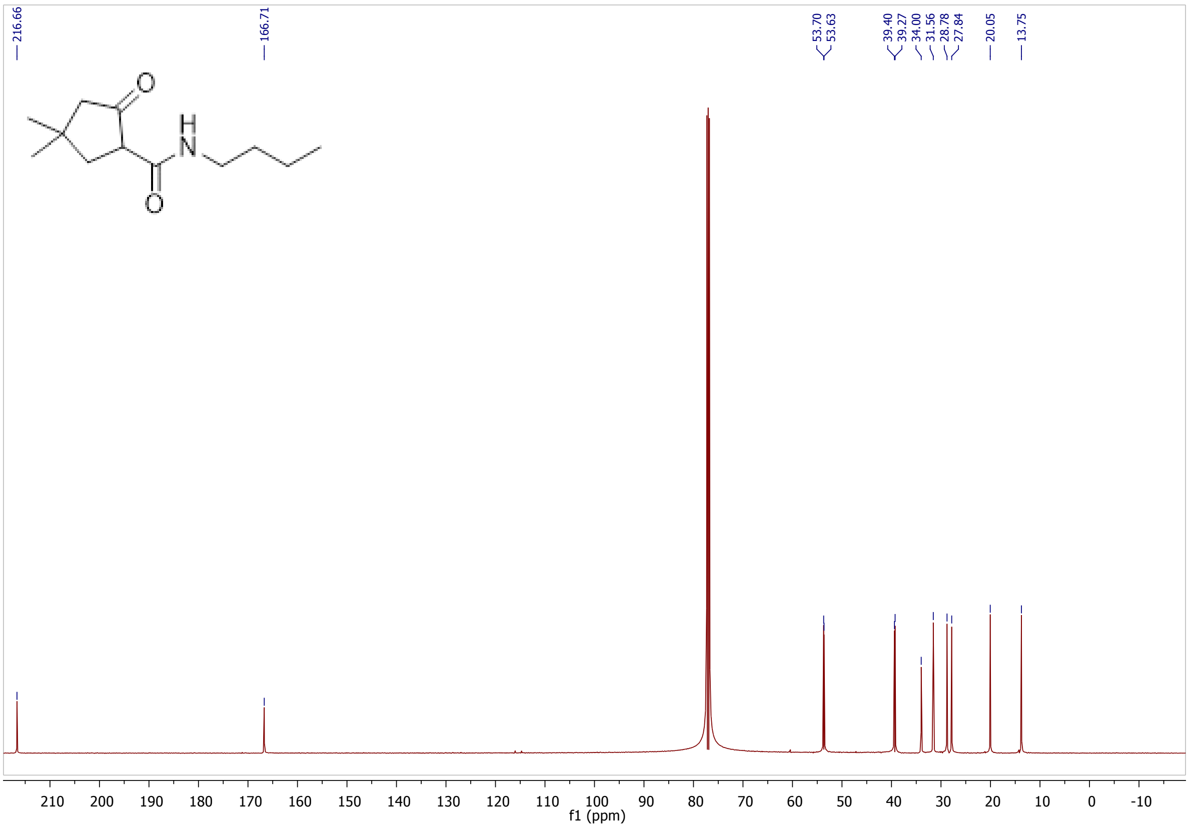

N-benzyl 4,4-dimethyl-2-oxocyclopentane-1-carboxamide (4b)

^{1}H NMR (500 MHz, $CDCl_3$)

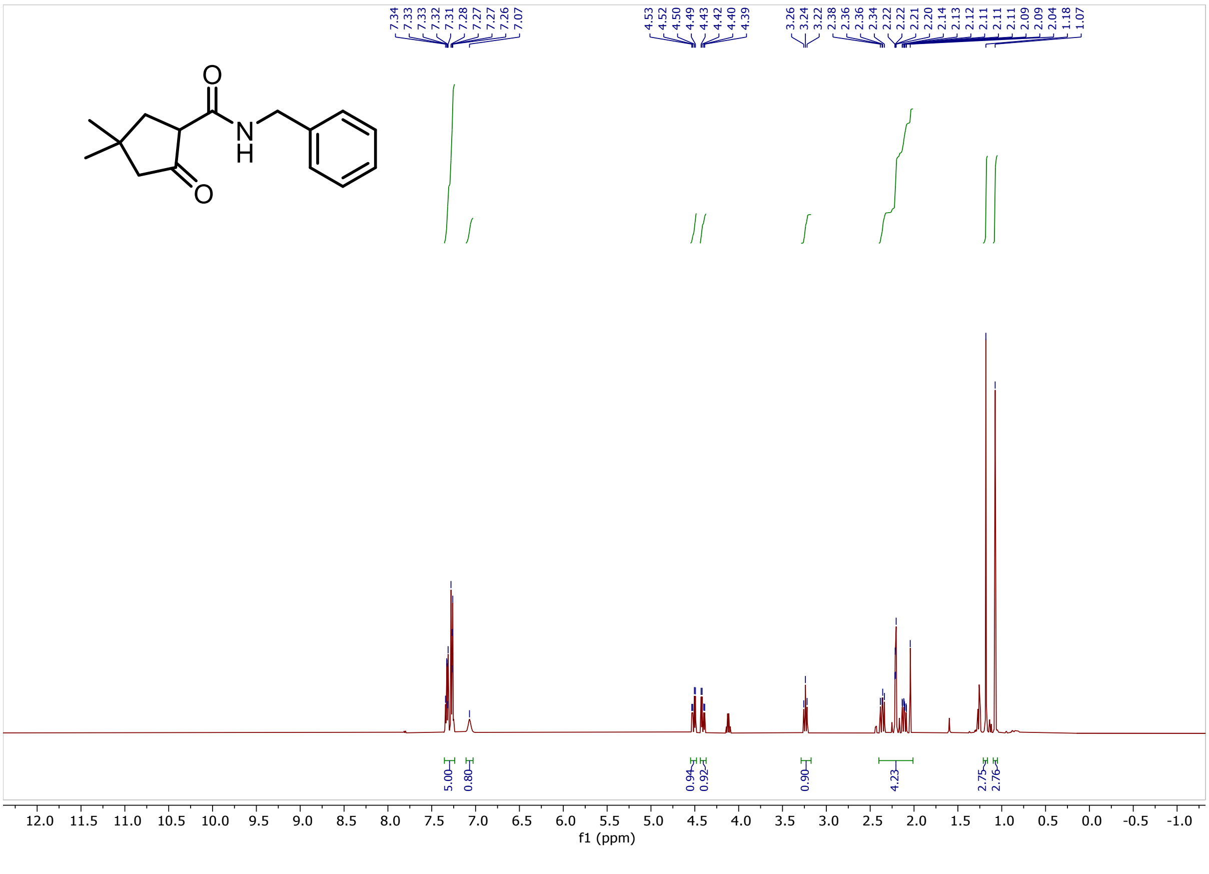

13C{1H} NMR (126 MHz, CDCl3)

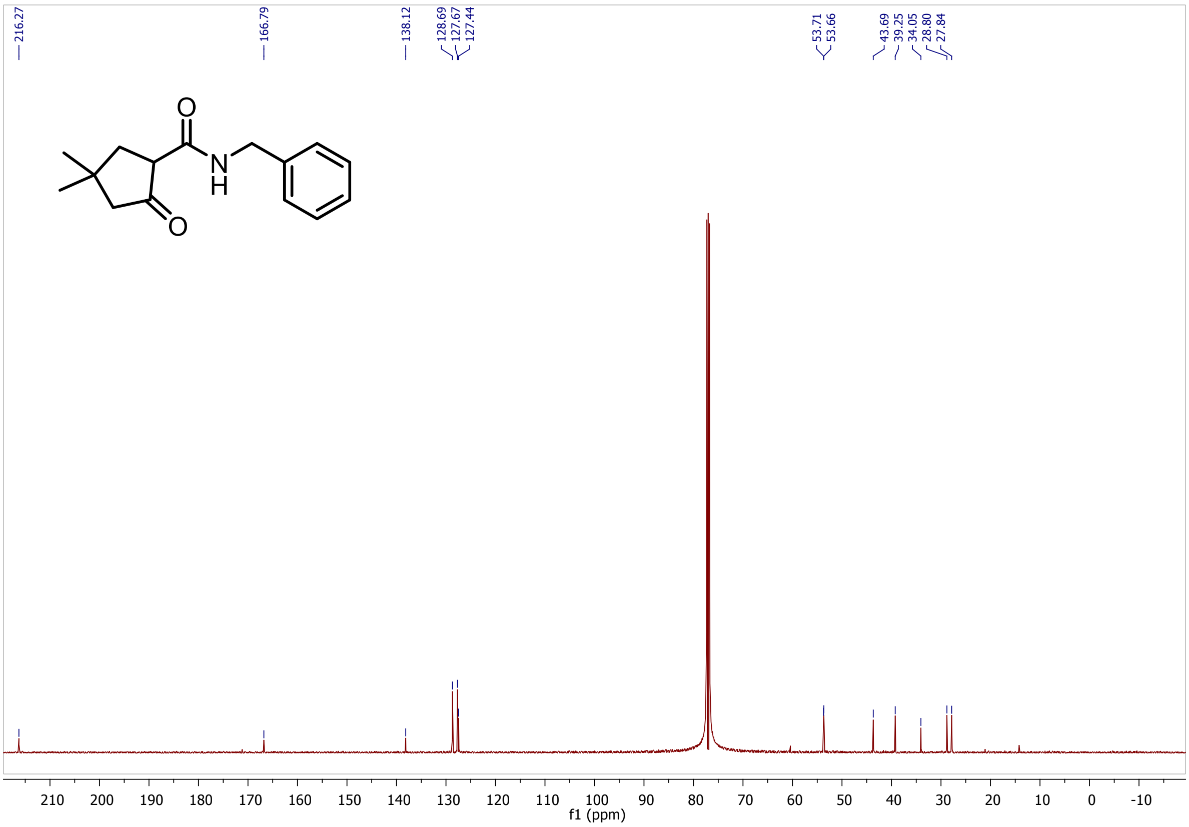

4,4-dimethyl-2-(piperidine-1-carbonyl)cyclopentane-1-one (4c)

^{1}H NMR (500 MHz, $CDCl_3$)

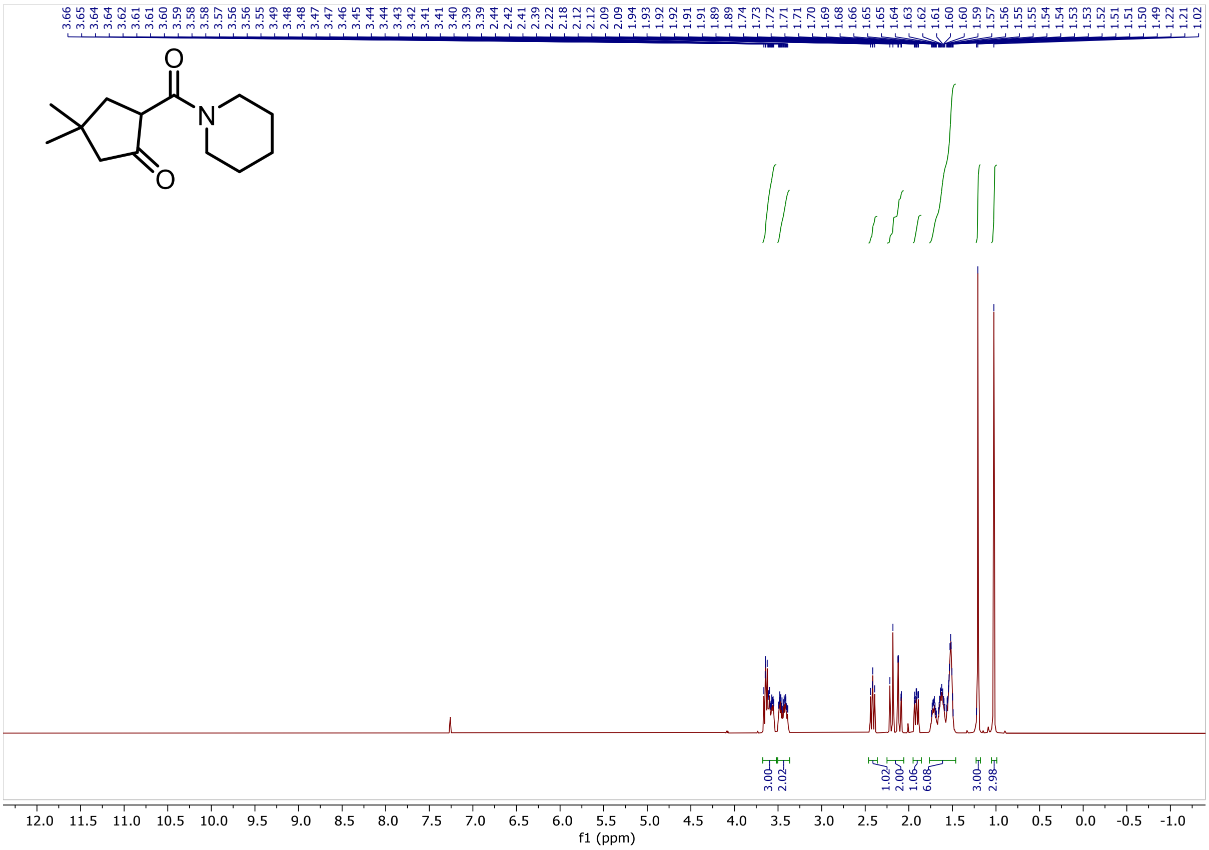

13C{1H} NMR (126 MHz, CDCl3)

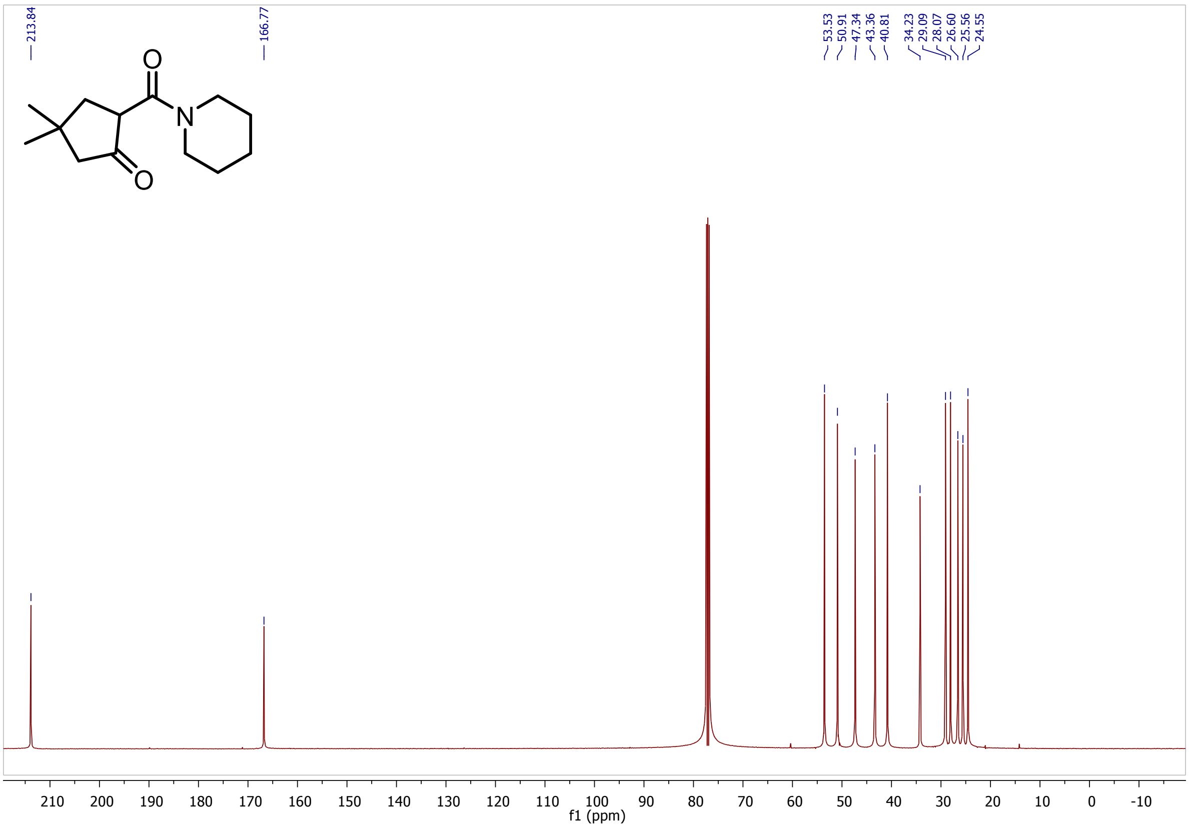

N,N-bis(4-(tert-butyl)phenyl-4,4-dimethyl-2-oxocyclopentane-1-carboxamide (4d)

^{1}H NMR (500 MHz, $CDCl_3$)

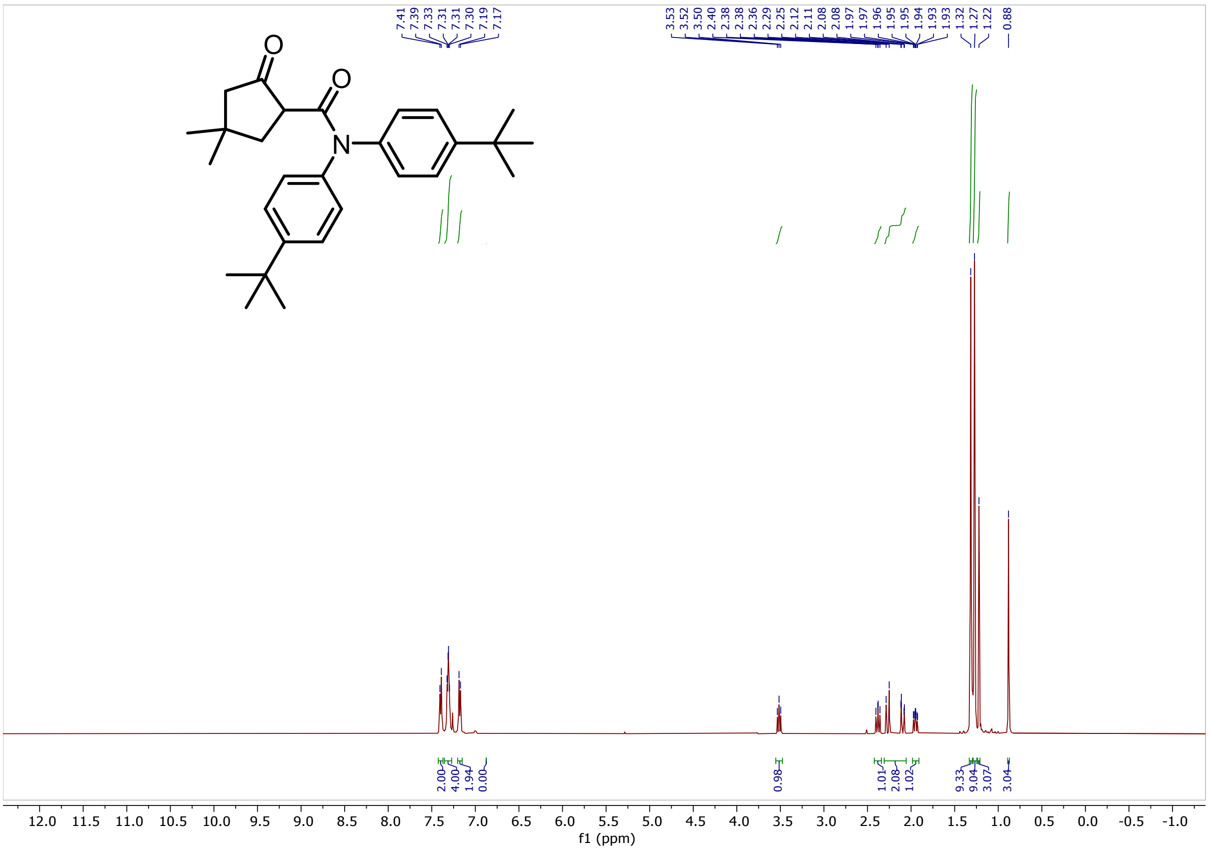

13C{1H} NMR (126 MHz, CDCl3)

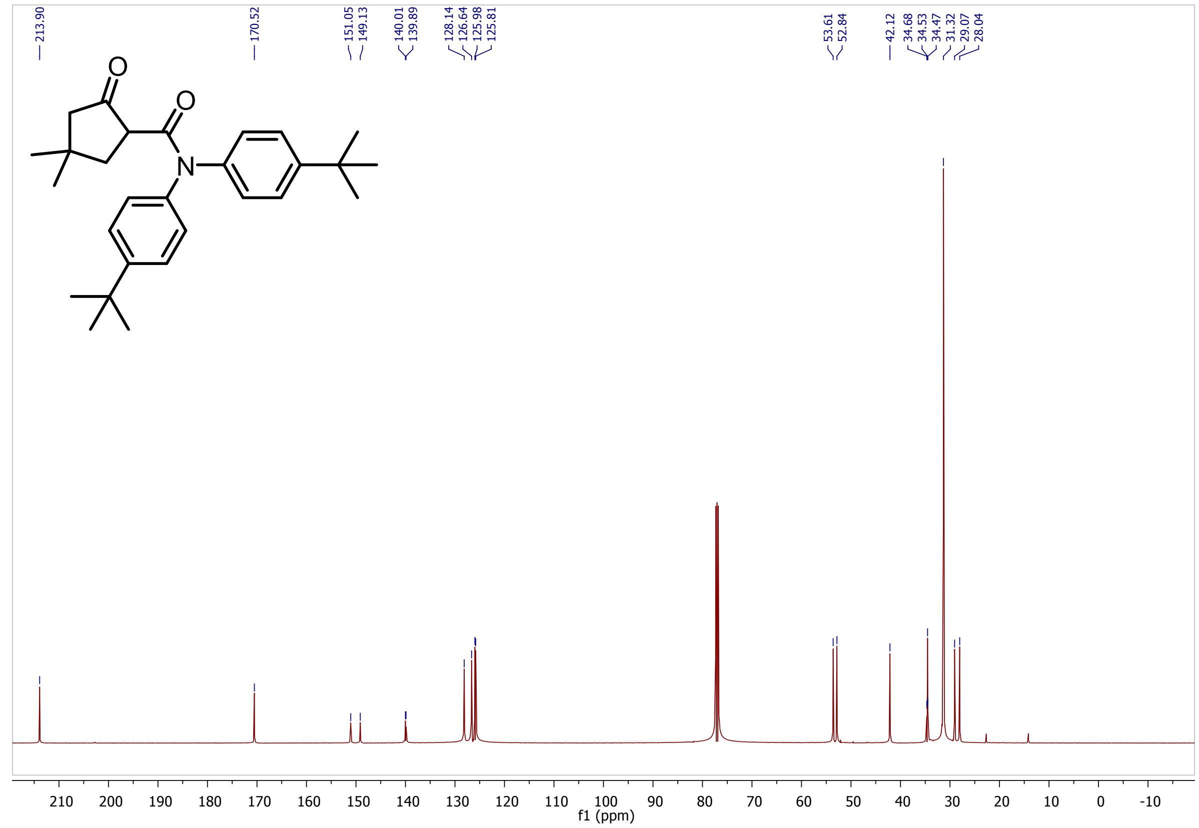

4,4-dimethyl-2-oxo-*N*-(prop-2-yn-1-yl)cyclopentanecarboxamide (4e)

^{1}H NMR (400 MHz, CDCl$_3$)

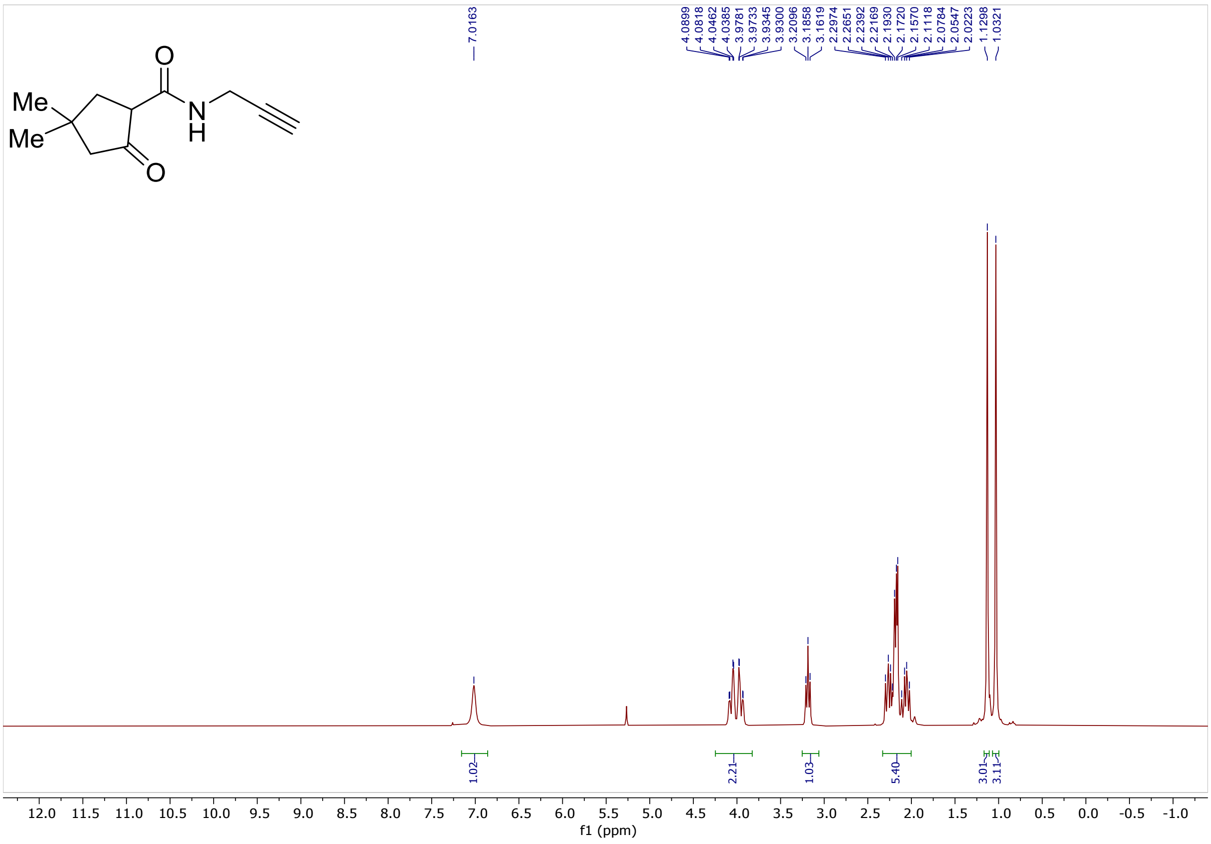

13C{1H} NMR (100 MHz, CDCl3)

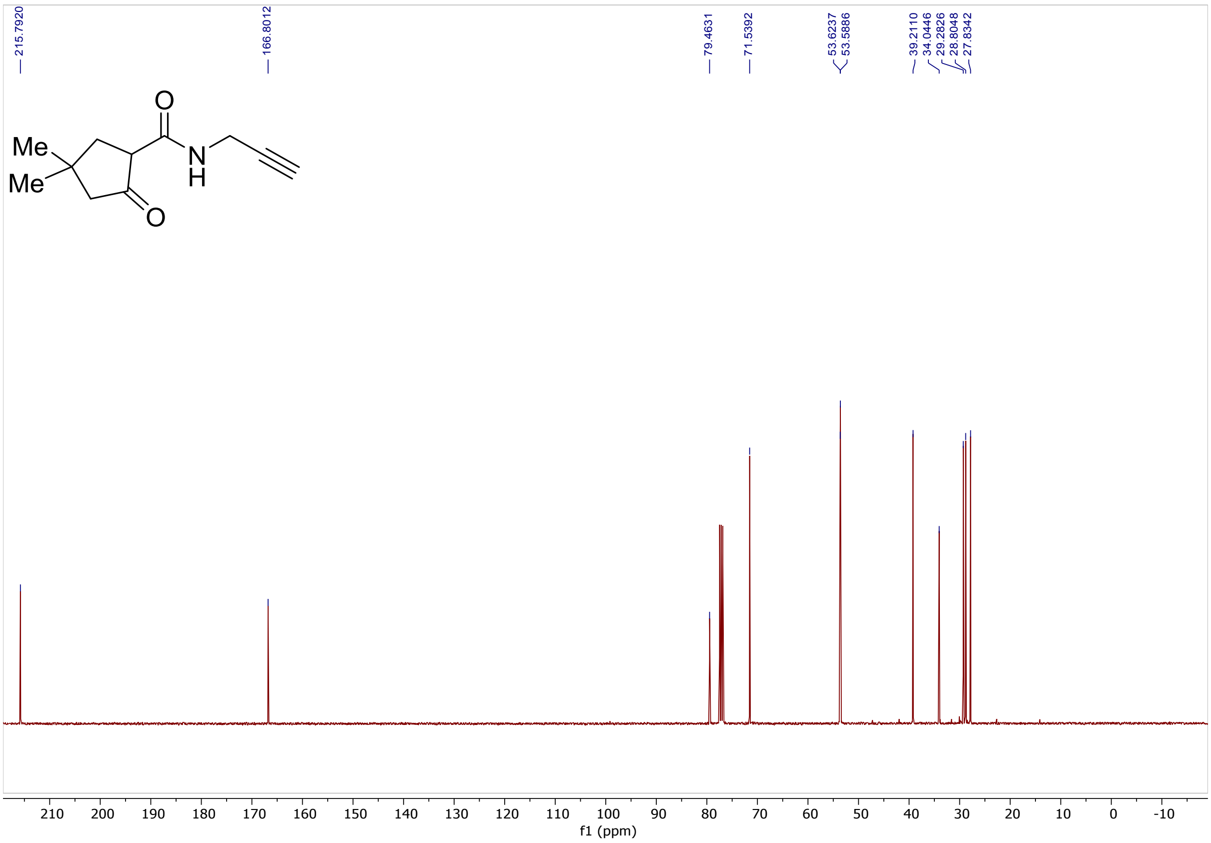

4,4-dimethyl-2-oxo-N-phenylcyclopentane-1-carboxamide (4f)

^{1}H NMR (500 MHz, $CDCl_3$)

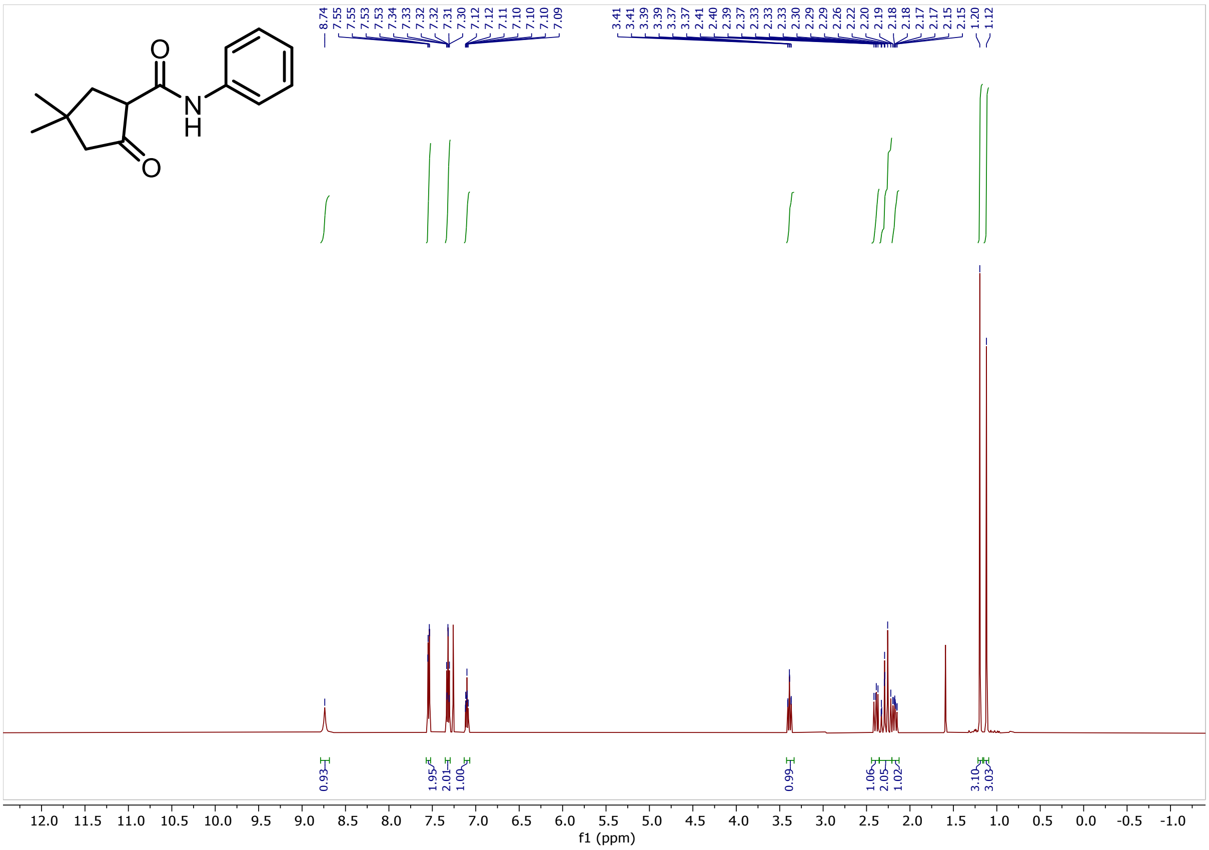

13C{1H} NMR (126 MHz, CDCl3)

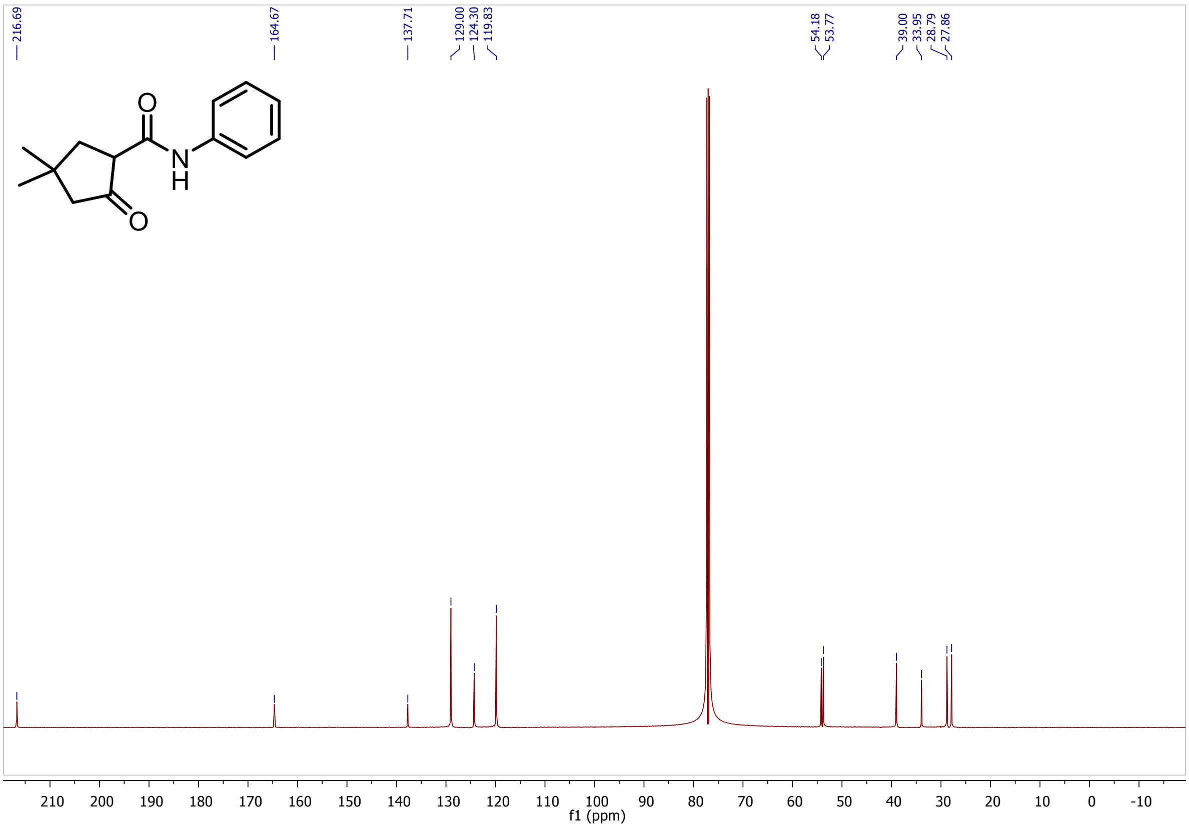

***N*-cyclopropyl-4,4-dimethyl-2-oxocyclopentanecarboxamide (4g)**

^{1}H NMR (400 MHz, $CDCl_3$)

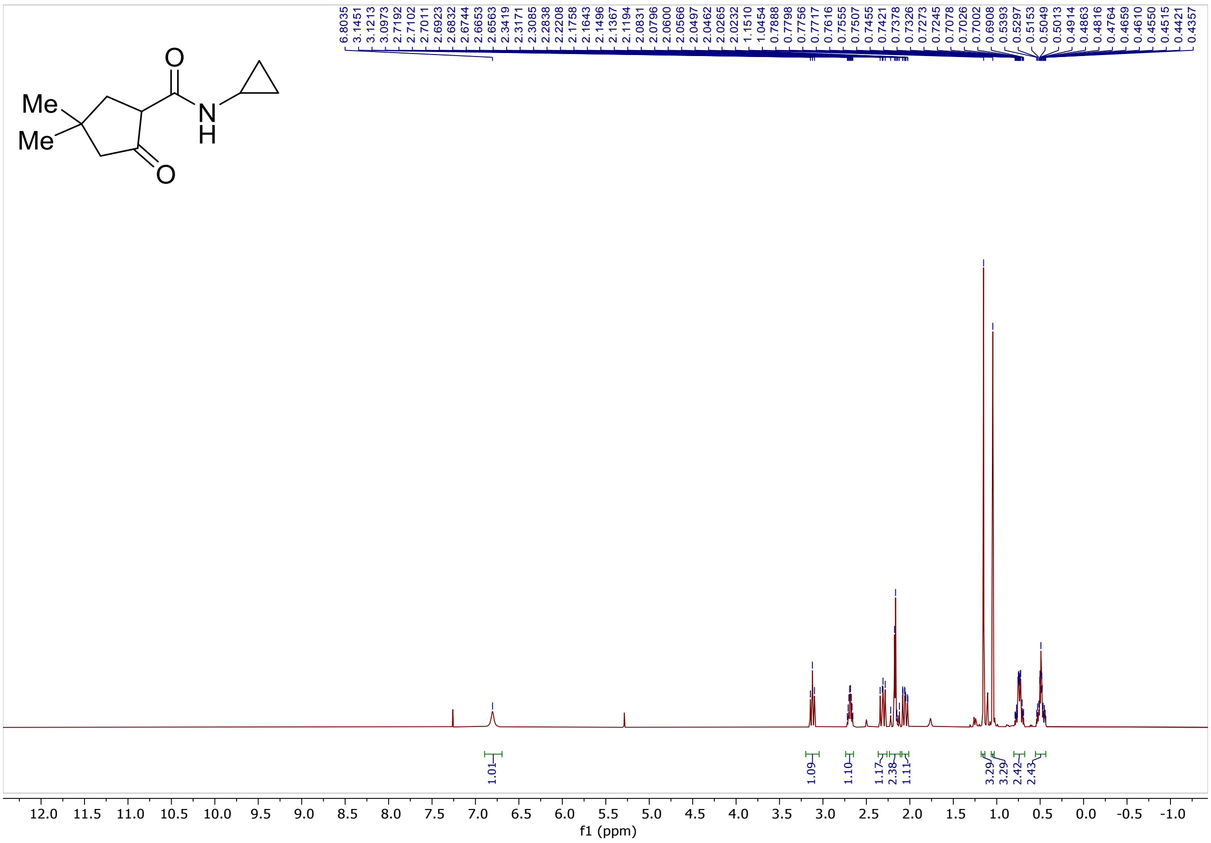

13C{1H} NMR (100 MHz, CDCl3)

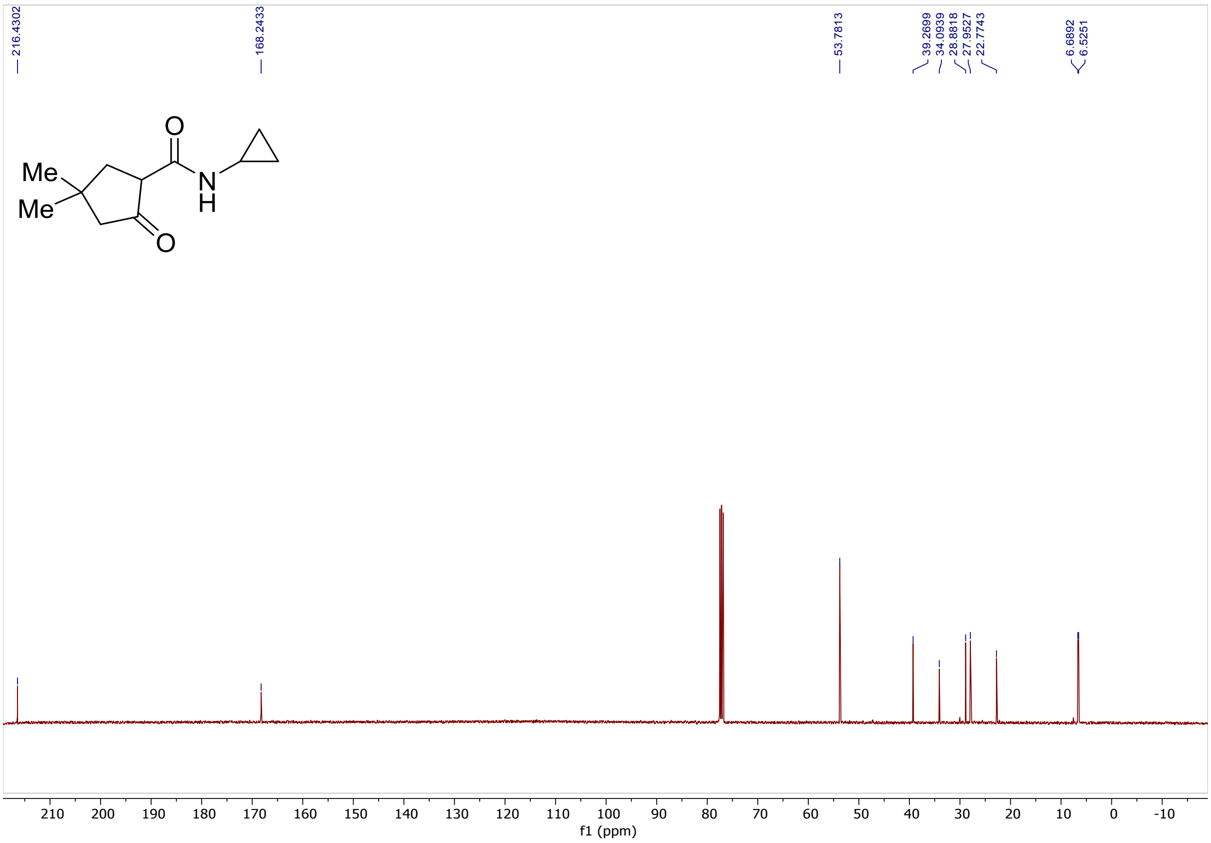

N-cyclobutyl-4,4-dimethyl-2-oxocyclopentanecarboxamide (4h)

^{1}H NMR (400 MHz, $CDCl_3$)

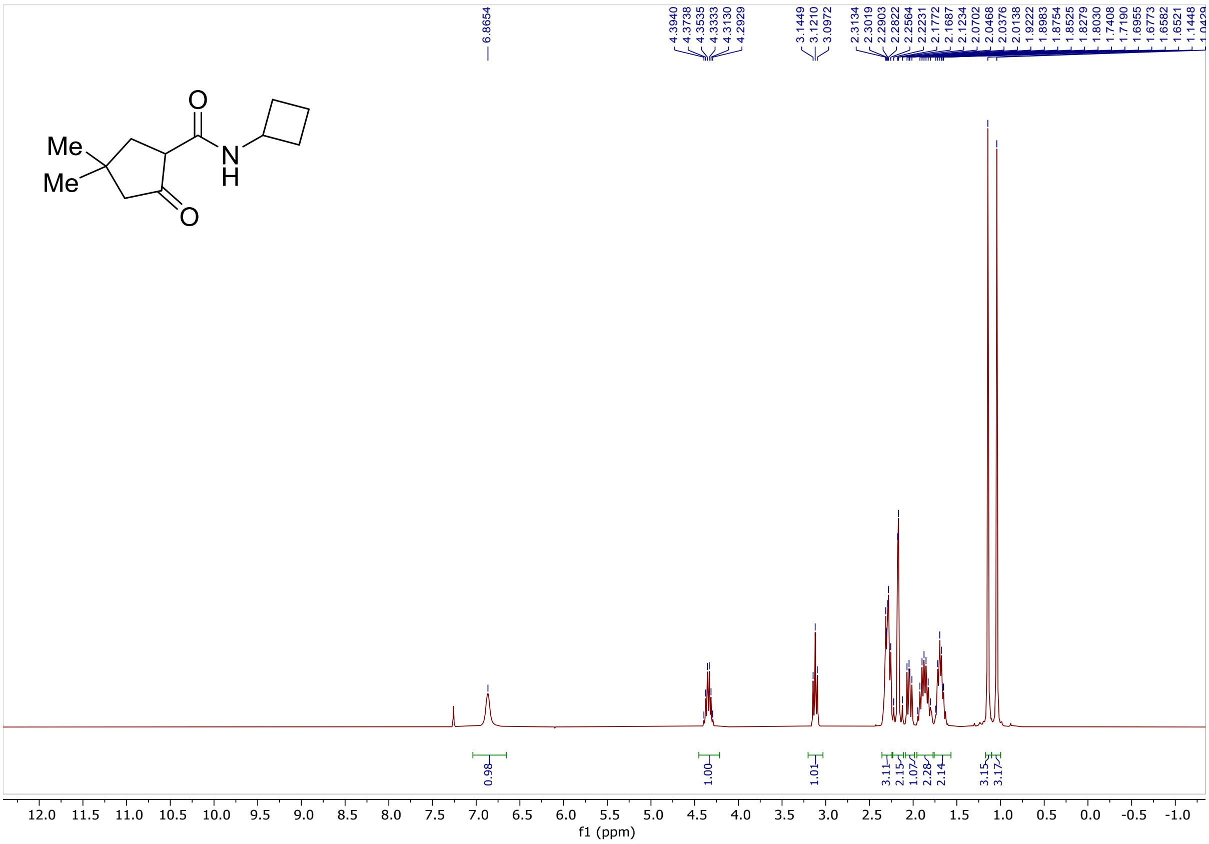

13C{1H} NMR (100 MHz, CDCl3)

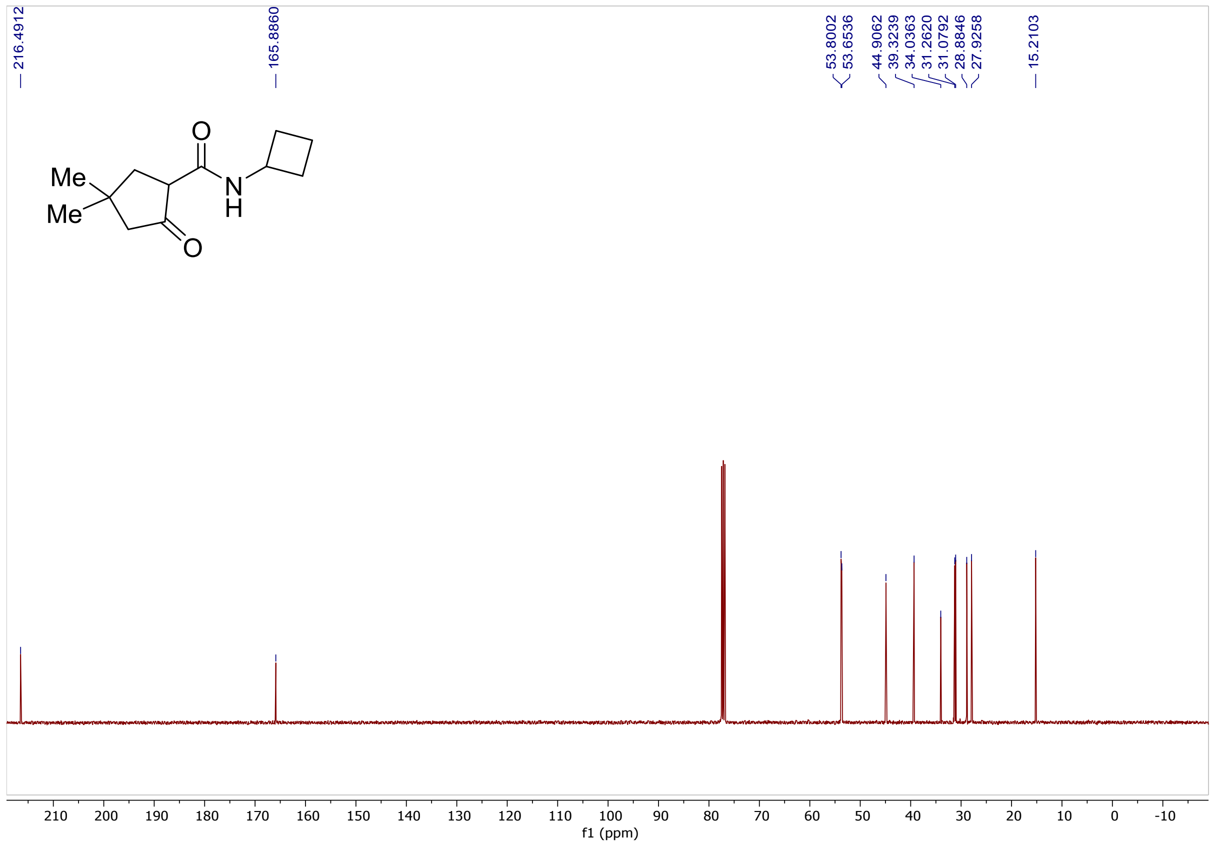

N-((3s,5s,7s)-adamantan-1-yl)-4,4-dimethyl-2-oxocyclopentanecarboxamide (4i)

^{1}H NMR (400 MHz, $CDCl_3$)

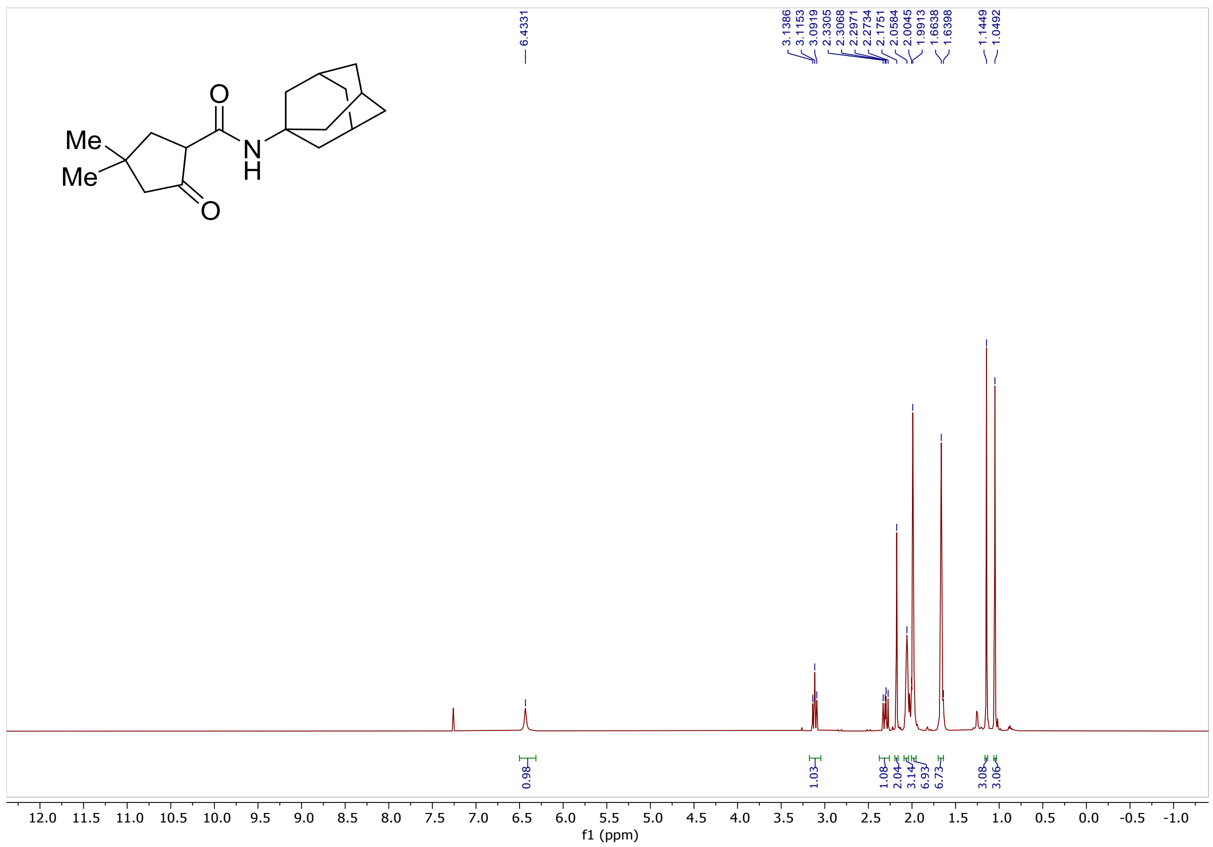

13C{1H} NMR (100 MHz, CDCl3)

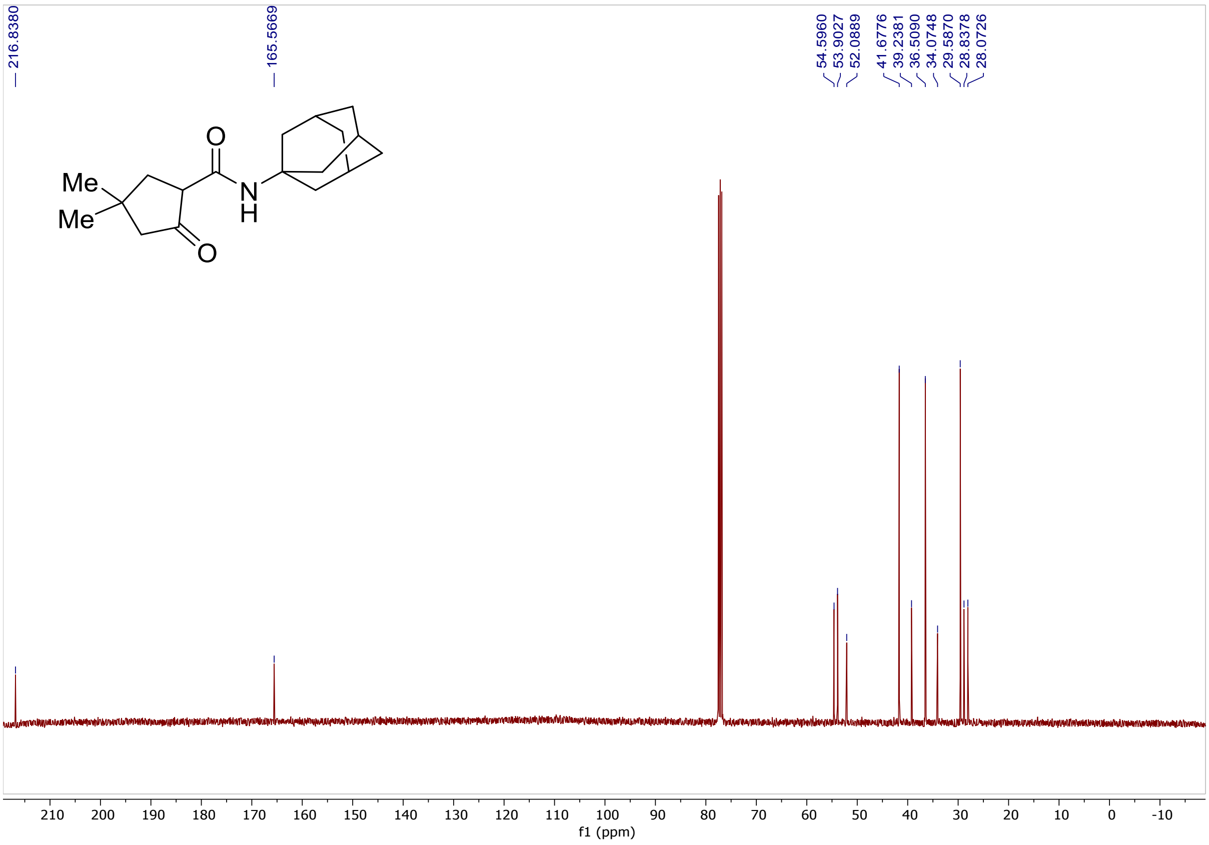

3-ethyl-6,6-dimethyl-6,7-dihydrocyclopenta[e][1,3]oxazine-2,4-(3H,5H)-dione (5a)

^{1}H NMR (500 MHz, $CDCl_3$)

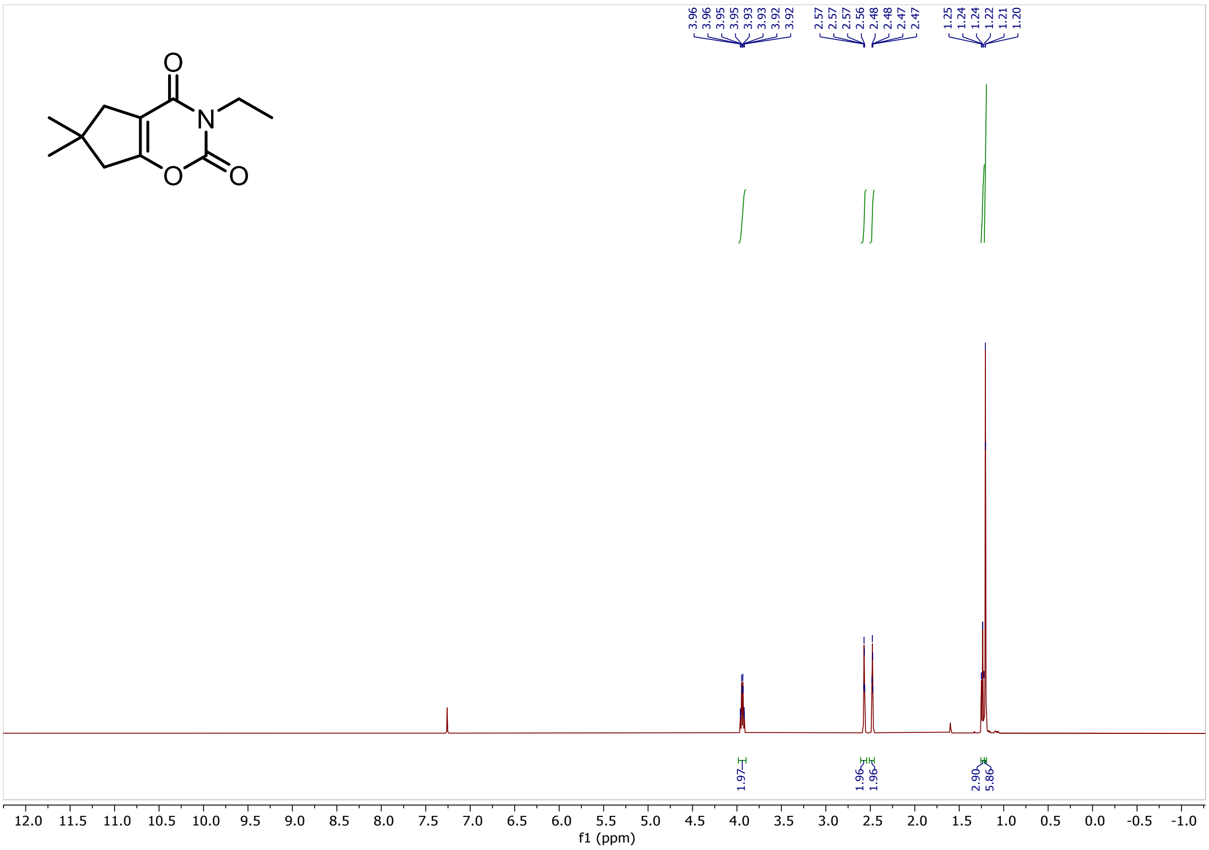

13C{1H} NMR (126 MHz, CDCl3)

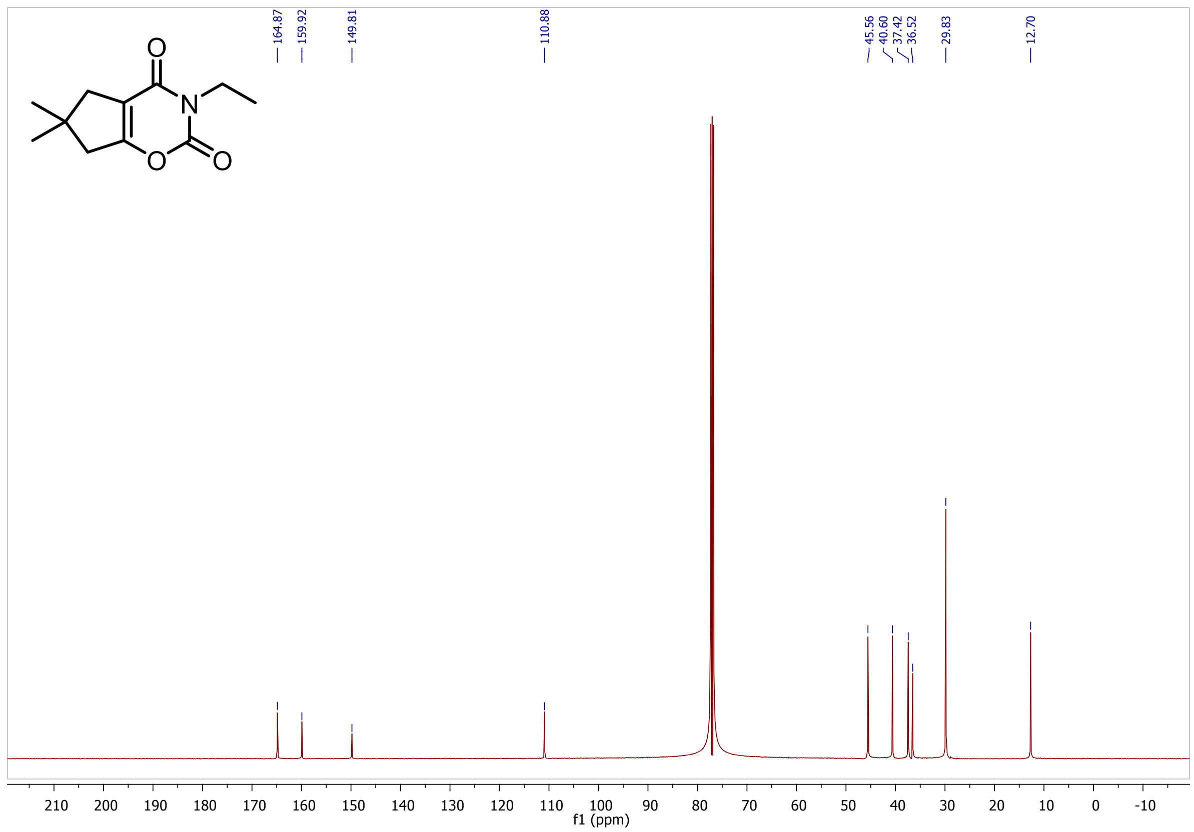

6,6-dimethyl-3-phenyl-6,7-dihydrocyclopenta[e][1,3]oxazine-2,4-(3H,5H)-dione (5b)

^{1}H NMR (500 MHz, $CDCl_3$)

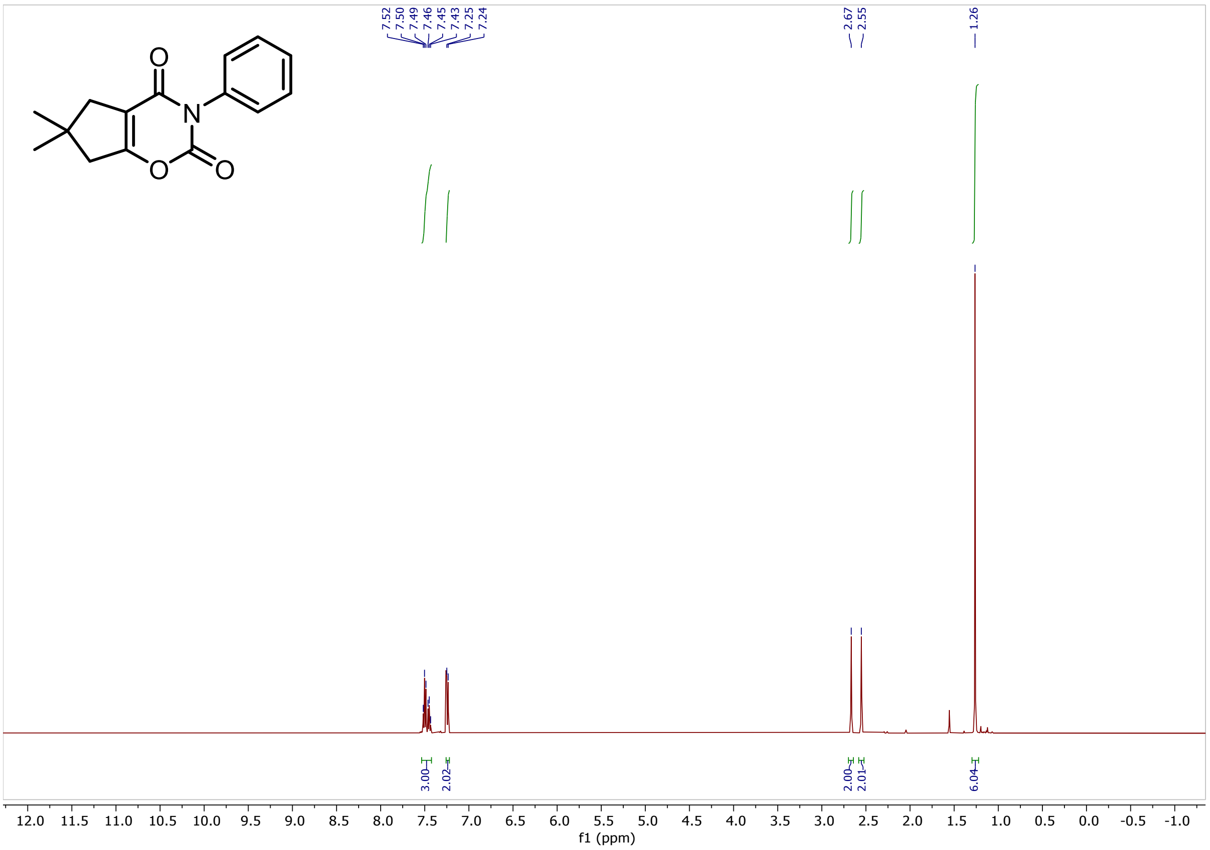

13C{1H} NMR (126 MHz, CDCl3)

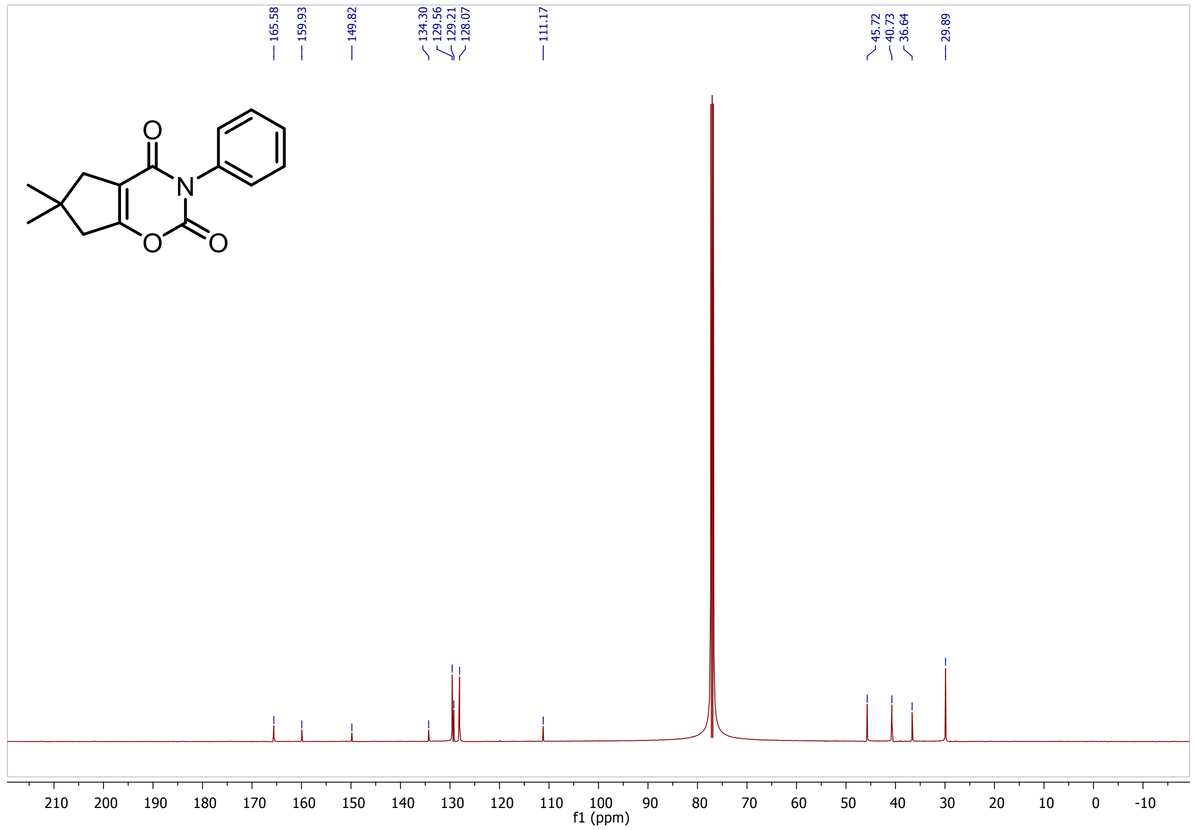

3-(3-acetylphenyl)-6,6-dimethyl-6,7-dihydrocyclopenta[e][1,3]oxazine-2,4-(3H,5H)-dione (5c)

^{1}H NMR (500 MHz, $CDCl_3$)

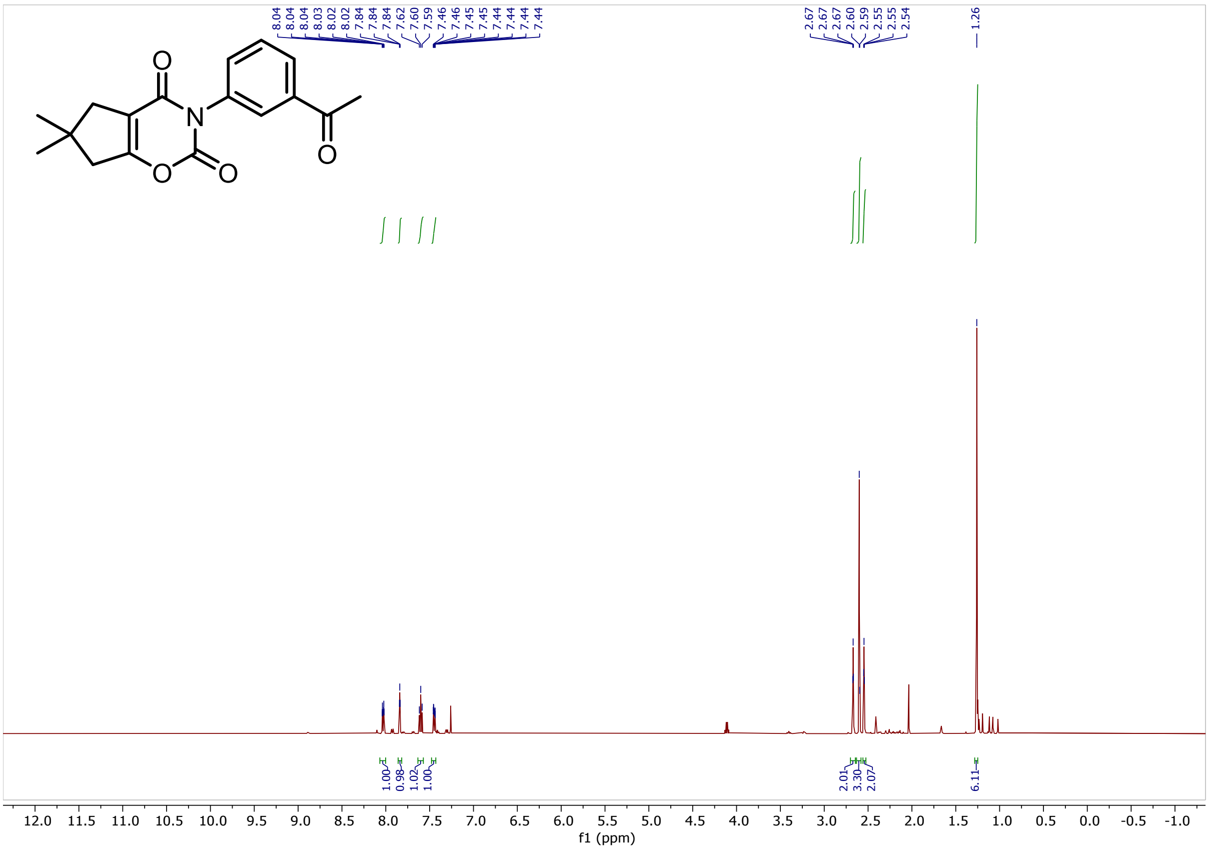

13C{1H} NMR (126 MHz, CDCl3)

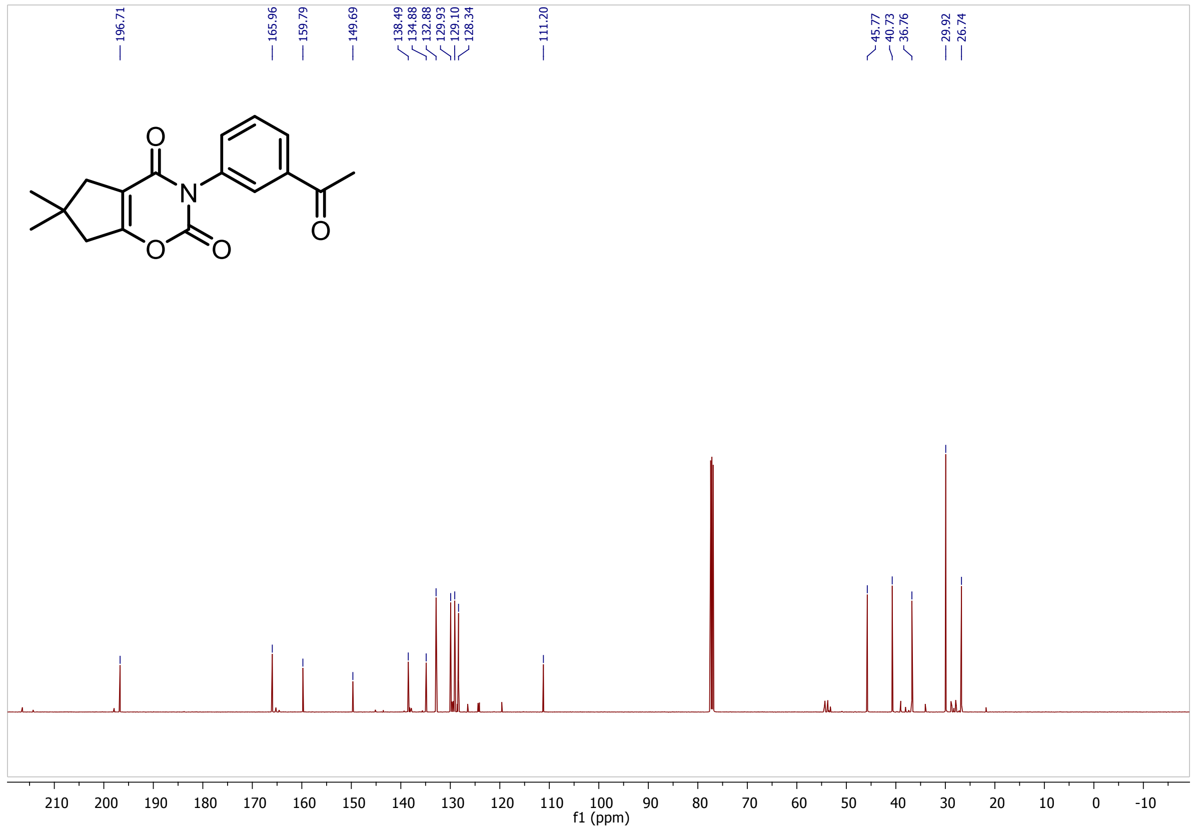

3-(4-chlorophenyl)-6,6-dimethyl-6,7-dihydrocyclopenta[e][1,3]oxazine-2,4-(3H,5H)-dione (5d)

^{1}H NMR (500 MHz, $CDCl_3$)

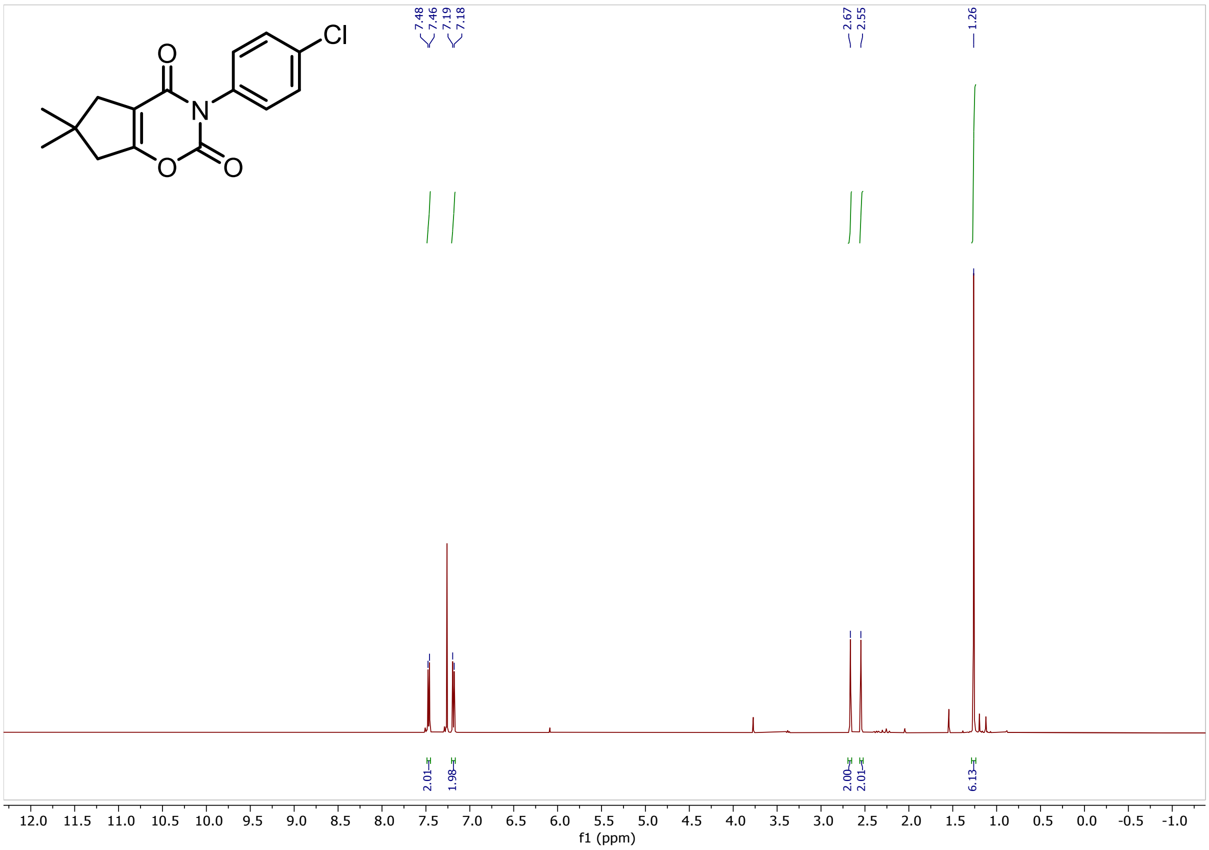

13C{1H} NMR (126 MHz, CDCl3)

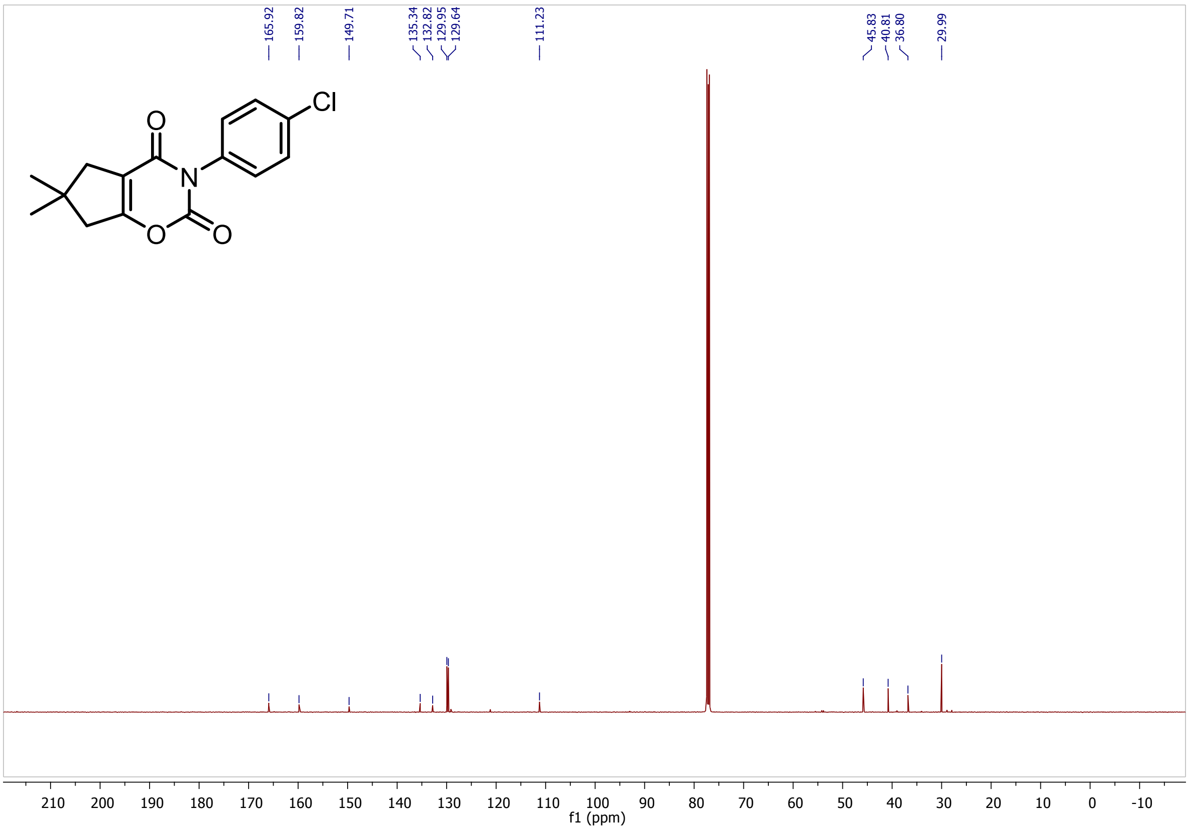

3-(4-methoxyphenyl)-6,6-dimethyl-6,7-dihydrocyclopenta[e][1,3]oxazine-2,4-(3H,5H)-dione (5e)

^{1}H NMR (500 MHz, $CDCl_3$)

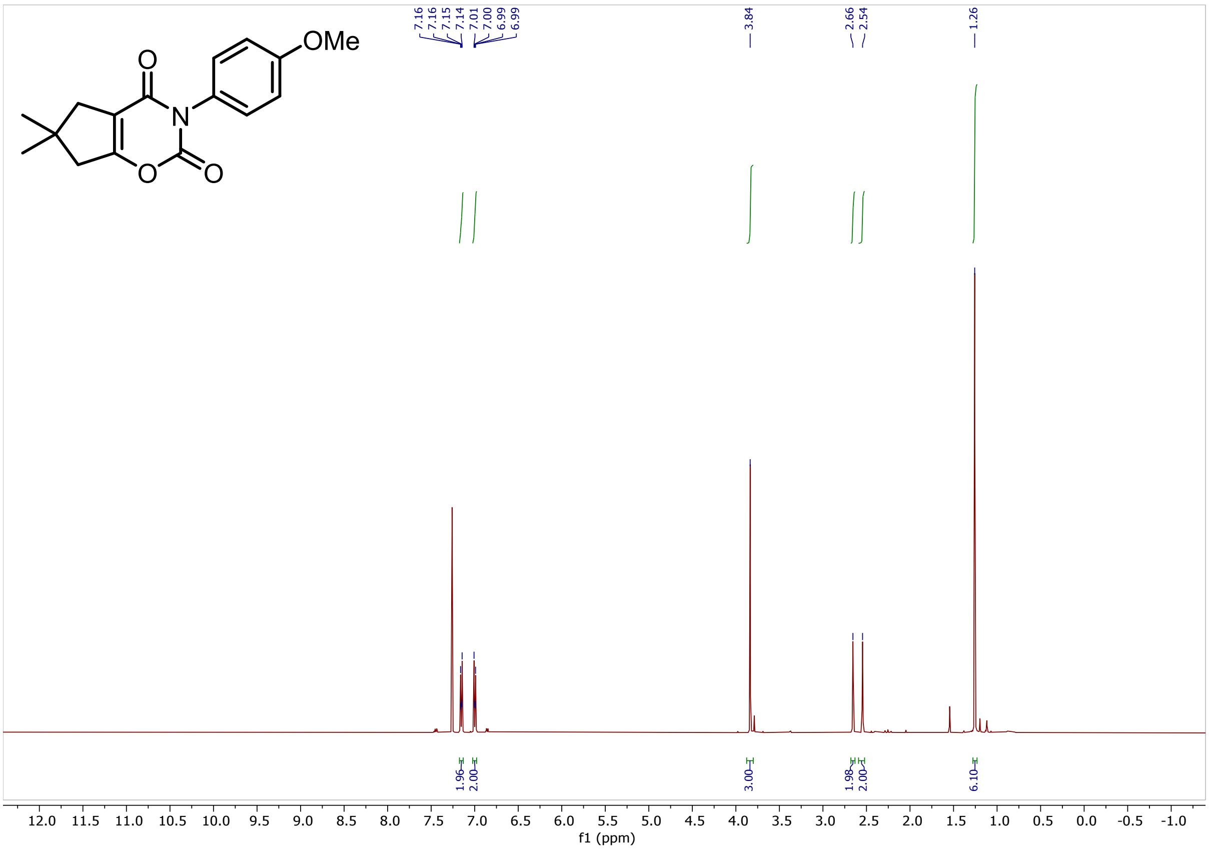

13C{1H} NMR (126 MHz, CDCl3)

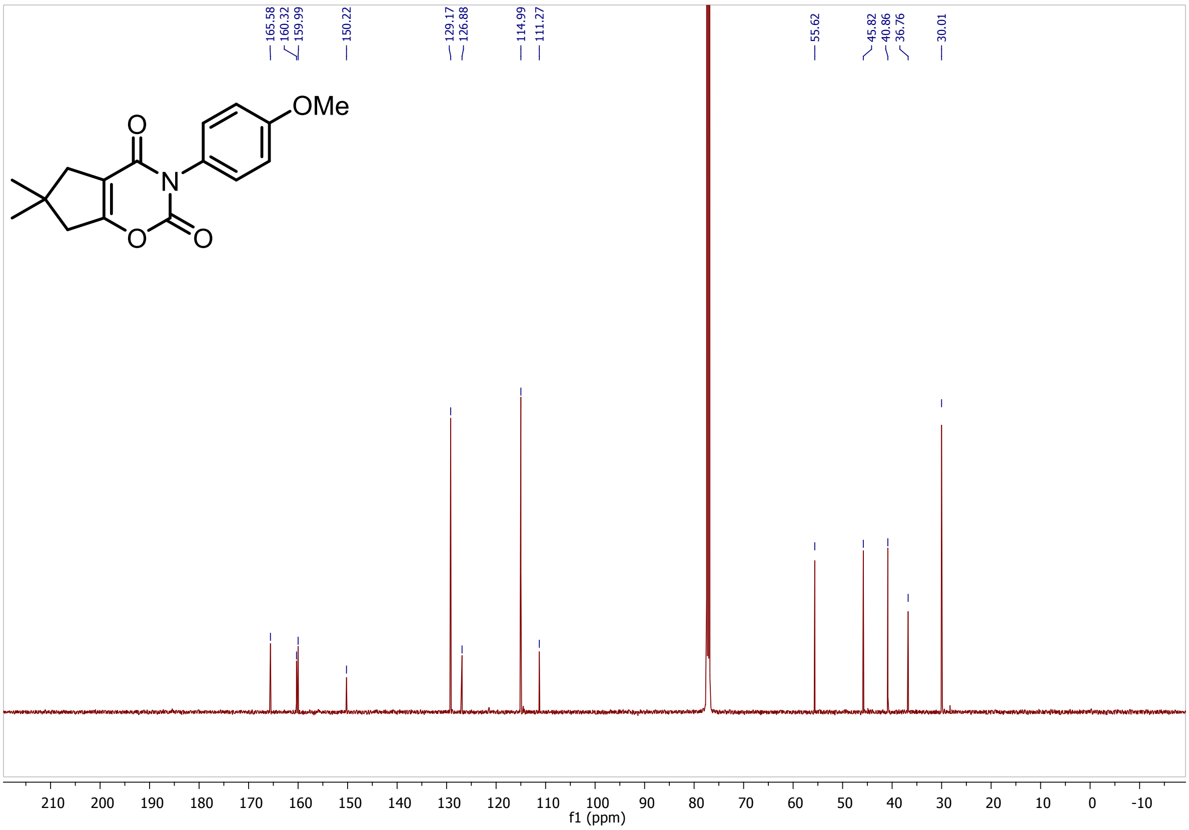
165.58
160.32
159.99
150.22
129.17
126.88
114.99
111.27
55.62
45.82
40.86
36.76
30.01
OMe
O
N
O
O
210
200
190
180
170
160
150
140
130
120
110
100
90
80
70
60
50
40
30
20
10
0
-10
f1 (ppm)
